# Modeling of Chemical Reaction Systems with Detailed Balance Using Gradient Structures

**DOI:** 10.1007/s10955-020-02663-4

**Published:** 2020-11-06

**Authors:** Jan Maas, Alexander Mielke

**Affiliations:** 1 Institute of Science and Technology Austria (IST Austria), Am Campus 1, 3400 Klosterneuburg, Austria; 2 WIAS Berlin, Mohrenstraße 39, 10117 Berlin, Germany; 3 Institut für Mathematik, Humboldt-Universität zu Berlin, Unter den Linden 6, 10119 Berlin, Germany

**Keywords:** Chemical master equation, Reaction-rate equation, Gradient flow, Detailed balance, Hybrid models

## Abstract

We consider various modeling levels for spatially homogeneous chemical reaction systems, namely the chemical master equation, the chemical Langevin dynamics, and the reaction-rate equation. Throughout we restrict our study to the case where the microscopic system satisfies the detailed-balance condition. The latter allows us to enrich the systems with a gradient structure, i.e. the evolution is given by a gradient-flow equation. We present the arising links between the associated gradient structures that are driven by the relative entropy of the detailed-balance steady state. The limit of large volumes is studied in the sense of evolutionary $\Gamma$-convergence of gradient flows. Moreover, we use the gradient structures to derive hybrid models for coupling different modeling levels.

## Introduction

In this work we discuss different models for chemical reactions taking place in a container of volume *V*. Throughout we assume that the spatial extent of the container and the position of the chemical species are irrelevant, which means that we are looking at a well-stirred system. We assume that the system is composed of *I* different species named $X_1$ to $X_I$, which may represent different molecules, e.g., $X_1={\mathrm {H}}_2$, $X_2={\mathrm {O}}_2$, and $X_3={\mathrm {H}}_2{\mathrm {O}}$. We assume that these *I* species undergo *R* different reactions of mass-action type:1.1where the vectors ${\varvec{\alpha }}^r, {\varvec{\beta }}^r\in {\mathbb {N}}_0^I$ contain the stoichiometric coefficients, and $k^r_{\mathrm {fw}}, k^r_{\mathrm {bw}}> 0$ are the forward and backward reaction rates, see Sect. 2. The reaction  would lead to the vectors ${\varvec{\alpha }}=(2,1,0)$ and ${\varvec{\beta }}=(0,0,2)$.

Denoting by ${\varvec{c}}=(c_1,\ldots , c_I) \in {\varvec{C}}:={[0,\infty [}^I$ the vector of nonnegative densities, the simplest model is the macroscopic ***reaction-rate equation*** (RRE), which is a system of ODEs on the state space ${\varvec{C}}$:

RRE $$\begin{aligned} {\dot{{\varvec{c}}}} = -{\varvec{R}}({\varvec{c}}) \quad \text {with }{\varvec{R}}({\varvec{c}}):=\sum _{r=1}^R \big (k^r_{\mathrm {fw}}{\varvec{c}}^{{\varvec{\alpha }}^r} - k^r_{\mathrm {bw}}{\varvec{c}}^{{\varvec{\beta }}^r}\big )\big ( {\varvec{\alpha }}^r {-} {\varvec{\beta }}^r\big ). \end{aligned}$$

Here the monomials ${\varvec{c}}^{{\varvec{\alpha }}^r}:=\Pi _{i=1}^I c_I^{\alpha ^r_i}$ indicate that the probability for the right number of particles for the *r*th reaction to meet is given by a simple product of the corresponding densities, i.e., we assume that the positions of the particles are independent.

A truly microscopic model can be obtained as a stochastic process. Here we count the number of particles $N_i^V(t)$ for each species $X_i$ and consider the random vector ${\varvec{N}}^V(t)=(N_1^V(t),\ldots ,N_I^V(t)) \in {\mathcal {N}}:= {\mathbb {N}}_0^I$. A forward or backward reaction of type *r* is modeled as an instantaneous event where the particle numbers jump from ${\varvec{N}}^V(t) + {\varvec{\alpha }}^r$ to ${\varvec{N}}^V(t) + {\varvec{\beta }}^r$ or vice versa. The corresponding jump rates in a volume of size $V > 0$ are given by $k^r_{\mathrm {fw}}\!\;{\mathbb {B}}_{V}^{{\varvec{\alpha }}^r}\!({\varvec{N}}^V(t))\!\;$ and $k^r_{\mathrm {bw}}\!\;{\mathbb {B}}_{V}^{{\varvec{\beta }}^r}\!({\varvec{N}}^V(t))\!\;$ respectively; see (3.1) for the definition of $\;{\mathbb {B}}_{V}^{{\varvec{\alpha }}}\!({\varvec{n}})\!\;$.

Here we study the vector of probabilities

$$\begin{aligned} {\varvec{u}}(t) \in {\mathscr {P}}({\mathcal {N}}) := \big \{\, {\varvec{v}}=(v_{\varvec{n}})_{{\varvec{n}}\in {\mathcal {N}}} \, \big | \, v_{\varvec{n}}\ge 0,\ \sum \nolimits _{{\varvec{n}}\in {\mathcal {N}}} v_n=1 \,\big \} \end{aligned}$$

that describes the probability distribution of the random variable ${\varvec{N}}^V(t)$. The time evolution of ${\varvec{u}}(t)$ is given by the ***chemical master equation*** (CME), i.e., the Kolmogorov forward equation associated with the continuous time Markov chain above. This is a countable linear system of ODEs:

CME $$\begin{aligned} {\dot{{\varvec{u}}}}(t) = {\mathcal {B}}_V {\varvec{u}}(t), \qquad {\varvec{u}}(0) = {\varvec{u}}_0, \end{aligned}$$

where ${\mathcal {B}}_V$ is an (unbounded) linear operator on $\ell ^1({\mathcal {N}})$, see Sect. 3, where also existence and uniqueness of solutions is discussed. We refer to [39] for a short introduction to the CME and to [23] for a justification.

The basis of this work is the observation from [40] that (RRE) can be interpreted as a gradient flow if the reaction system satisfies the detailed-balance condition, i.e., there exists a positive equilibrium ${\varvec{c}}_*=(c_i^*)_{i=1,\ldots ,I} \in {]0,\infty [}^I$ such that

$$\begin{aligned} \kappa _*^r := k^r_{\mathrm {fw}}{\varvec{c}}_*^{{\varvec{\alpha }}^r} = k^r_{\mathrm {bw}}{\varvec{c}}_*^{{\varvec{\beta }}^r} \ \text { for } r = 1, \ldots , R. \end{aligned}$$

Defining the Boltzmann entropy *E* and the Onsager operator ${\mathbb {K}}$ via

$$\begin{aligned}&E({\varvec{c}}) = \sum _{i=1}^I \lambda _{\mathrm {B}}\big (\frac{c_i}{c_i^*} \big )c_i^* \qquad \text {with }\lambda _{\mathrm {B}}(z)=z \log z - z +1 \text { and}\\&{\mathbb {K}}({\varvec{c}})= \sum _{r=1}^R \kappa _*^r \,\Lambda \big ( \frac{{\varvec{c}}^{{\varvec{\alpha }}^r}}{{\varvec{c}}_*^{{\varvec{\alpha }}^r}}, \frac{{\varvec{c}}^{{\varvec{\beta }}^r}}{{\varvec{c}}_*^{{\varvec{\beta }}^r}}\big ) \, \big ({\varvec{\alpha }}^r{-}{\varvec{\beta }}^r\big ) {\otimes }\big ({\varvec{\alpha }}^r{-}{\varvec{\beta }}^r\big ) \ \in {\mathbb {R}}^{I{\times }I}_{\text {sym},\ge 0} \end{aligned}$$

where $\Lambda (a,b)=\int _0^1 a^s b^{1-s}\;\!\mathrm {d}s$ is the logarithmic mean, we see that (RRE) is generated by the gradient system $({\varvec{C}},E,{\mathbb {K}})$, namely ${\dot{{\varvec{c}}}} =-{\varvec{R}}({\varvec{c}})=- {\mathbb {K}}({\varvec{c}}){\mathrm {D}}E({\varvec{c}})$. In Sect. 2.5 we also discuss further gradient structures, e.g. those used in [43, 44, 46, 47].

If (RRE) satisfies the detailed-balance condition, then (CME) does so with an equilibrium distribution ${\varvec{w}}^V \in {\mathscr {P}}({\mathcal {N}})$ that is explicitly given as a product of one-dimensional Poisson distributions with mean $c_i^* V$, namely (cf. Theorem 3.1),

$$\begin{aligned} w_{\varvec{n}}^V = \prod _{i=1}^I {\mathrm {e}}^{-c_i^*V} \frac{(c_i^*V)^{n_i}}{n_i!} \ \text { for all } {\varvec{n}}= (n_1,\ldots ,n_I) \in {\mathcal {N}}. \end{aligned}$$

Consequently, we are also able to interpret (CME) as a gradient flow induced by a gradient system $({\mathscr {P}}({\mathcal {N}}), {\mathcal {E}}_V,{\mathcal {K}}_V)$, see (3.7). Here ${\mathcal {E}}_V({\varvec{u}})$ is again the Boltzmann entropy with respect to ${\varvec{w}}^V$, but now divided by the volume *V*:

1.2 $$\begin{aligned} {\mathcal {E}}_V({\varvec{u}})=\frac{1}{V} \sum _{n\in {\mathcal {N}}} \lambda _{\mathrm {B}}\bigg (\frac{u_{\varvec{n}}}{w^V_{\varvec{n}}}\bigg )w^V_{\varvec{n}}= \frac{1}{V} \sum _{n\in {\mathcal {N}}} u_{\varvec{n}}\log u_{\varvec{n}}+ \sum _{{\varvec{n}}\in {\mathcal {N}}} u_{\varvec{n}}\frac{1}{V} \log \frac{1}{w^V_{\varvec{n}}}. \end{aligned}$$

### Large-volume approximations using gradient structures

A major challenge in modeling chemical reactions is the question of understanding the transition from small-volume effects to the macroscopic behavior in large volumes. The first breakthrough was obtained in [30–33] by connecting the particle numbers ${\varvec{N}}(t)\in {\mathcal {N}}$ to the concentrations ${\varvec{c}}\in {\varvec{C}}$ and showing that

1.3 $$\begin{aligned} \frac{1}{V} {\varvec{N}}^V(0)\rightarrow {\varvec{c}}_0 \text { almost surely implies } \frac{1}{V} {\varvec{N}}^V(t)\rightarrow {\varvec{c}}(t) \text { almost surely for all } t>0,\nonumber \\ \end{aligned}$$

where $t \mapsto {\varvec{c}}(t)$ is the solution of (RRE) with ${\varvec{c}}(0)={\varvec{c}}_0$. This result may be interpreted as a justification for the RRE in terms of the Markovian model. In [45, 46] a dynamic large deviation principle is applied to $\frac{1}{V} {\varvec{N}}^V(\cdot )$, which leads to a rate functional that generates a gradient structure $({\varvec{C}},{\mathcal {E}},\Psi _{\cosh })$; see Sect. 2.5. Recent large deviation results for chemical reaction networks can be found in [1, 2].

In this paper we study the limit $V\rightarrow \infty$ for the gradient system $({\mathscr {P}}({\mathcal {N}}), {\mathcal {E}}_V,{\mathcal {K}}_V)$, and hence for (CME), in the sense of evolutionary $\Gamma$-convergence for gradient systems, as introduced in [54, 58] and further developed in [13, 41]. For this purpose we use a suitable embedding $\iota _V: {\mathscr {P}}({\mathcal {N}}) \rightarrow {\mathscr {P}}({\varvec{C}})$ (Sect. 4) and obtain the coarse grained gradient system $({\mathscr {P}}({\varvec{C}}),{\varvec{E}},{\varvec{K}})$ with

$$\begin{aligned} {\varvec{E}}(\varrho )=\int _{\varvec{C}}E({\varvec{c}})\varrho ({\mathrm {d}}{\varvec{c}}) \quad \text {and} \quad \big ({\varvec{K}}(\varrho )\xi \big )({\varvec{c}}) = - {\mathrm {div}}_{{\varvec{c}}}\big (\varrho ({\varvec{c}}) {\mathbb {K}}({\varvec{c}}) \nabla _{{\varvec{c}}} \xi ({\varvec{c}}) \big ). \end{aligned}$$

In particular, the coarse grained gradient flow equation is the ***Liouville equation***

Lio $$\begin{aligned} {\dot{\varrho }}(t,{\varvec{c}}) = {\mathrm {div}}_{\varvec{c}}\big (\varrho (t,{\varvec{c}}) {\varvec{R}}({\varvec{c}})\big ),\qquad \varrho _{t = 0} = \varrho _0, \end{aligned}$$

associated with (RRE); here we used that $\xi = {\mathrm {D}}_\varrho {\varvec{E}}= E$ and ${\varvec{R}}=-{\mathbb {K}}{\mathrm {D}}_{\varvec{c}}E$. Thus, in this scaling a pure transport equation remains, while all diffusion disappears, as can be seen in the factor 1/*V* before the middle sum in (1.2). In particular, our result is consistent with Kurtz’ result (1.3): by assuming $\varrho (0) = \delta _{{\varvec{c}}_0} \in {\mathscr {P}}({\varvec{C}})$ we obtain $\varrho (t) = \delta _{{\varvec{c}}(t)}$. While Kurtz works directly on the Markovian random variables, we work at the level of their distributions: 
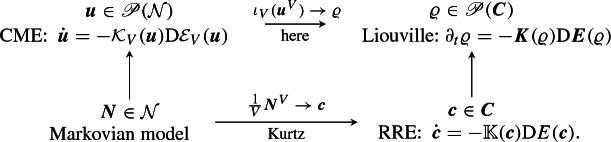


Our convergence result for the gradient systems $({\mathscr {P}}({\mathcal {N}}), {\mathcal {E}}_V,{\mathcal {K}}_V)$ to the limiting gradient system $({\mathscr {P}}({\varvec{C}}),{\varvec{E}},{\varvec{K}})$ can be seen as a concrete example of the *EDP convergence* of gradient systems as discussed in [13, 35]. Another example treating the convergence of “Markovian discretizations” towards a Fokker–Planck equation is studied in [11]; see also [16, 18, 56] for applications to interacting particle systems.

In addition to the extreme cases *V* finite and $V \rightarrow \infty$ it is also important to study the case of intermediate *V*, where $\frac{1}{V} {\varvec{N}}^V(t)$ already behaves continuously but still shows some fluctuations of standard deviation $1/\sqrt{V}$, see [61] for a numerical approach to treat the hierarchy via a suitable hybrid method. In [34] it is shown that the random vector $t\mapsto {\varvec{X}}^V(t)\in {\varvec{C}}$ obtained by solving the stochastic differential equation

1.4 $$\begin{aligned}&{\mathrm {d}}{\varvec{X}}^V(t) = -{\varvec{R}}({\varvec{X}}^V(t))\;\!\mathrm {d}t + \frac{1}{\sqrt{V}} \Big (\Sigma ^{\mathrm {fw}}({\varvec{X}}^V(t))\;\!\mathrm {d}{\mathfrak {B}}^{\mathrm {fw}}(t) + \Sigma ^{\mathrm {bw}}({\varvec{X}}^V(t))\;\!\mathrm {d}{\mathfrak {B}}^{\mathrm {bw}}(t)\Big ) \nonumber \\&\quad \text {with independent Brownian vectors }{\mathfrak {B}}^{\mathrm {fw}}(t),\, {\mathfrak {B}}^{\mathrm {bw}}(t) \in {\mathbb {R}}^R, \text { and } \nonumber \\&\quad \Sigma ^{\mathrm {fw}}({\varvec{X}})= \Big ( \big (\tfrac{\kappa ^r{\varvec{X}}^{{\varvec{\alpha }}^r}}{{\varvec{c}}_*^{{\varvec{\alpha }}^r}} \big )^{1/2} ({\varvec{\alpha }}^r{-}{\varvec{\beta }}^r) \Big )_r, \; \ \ \Sigma ^{\mathrm {bw}}({\varvec{X}})= \Big (\big ( \tfrac{\kappa ^r{\varvec{X}}^{{\varvec{\beta }}^r}}{{\varvec{c}}_*^{{\varvec{\beta }}^r}}\big )^{1/2} ({\varvec{\beta }}^r{-}{\varvec{\alpha }}^r) \Big )_r \in {\mathbb {R}}^{I{\times }R}, \end{aligned}$$

(see [34, Eq.  (1.7)]) yields an improved approximation because $\frac{1}{V} {\varvec{N}}^V(t)= {\varvec{X}}^V(t)+ O\big (({\log V})/V\big )$, while $\frac{1}{V} {\varvec{N}}^V(t)= {\varvec{c}}(t)+O\big (1/\sqrt{V}\big )$. This model is a so-called diffusion approximation, which in the reaction context also is termed ‘chemical Langevin dynamics’. In [24, Eq.  (23)] and [61, Eq.  (7)] the stochastic differential Eq. (1.4) is called ***chemical Langevin equation*** (CLE).

The associated Kolmogorov forward equation takes the form

1.5 $$\begin{aligned} {\dot{\rho }} = \frac{1}{V} \sum _{i,j=1}^I \partial _{ij}^2 \big ( \rho \, \widehat{\mathbb {K}}_{\mathrm {CLE}}({\varvec{c}})_{ij} \big ) + {\mathrm {div}}\big ( \rho {\varvec{R}}({\varvec{c}})\big ) \text { with } \widehat{\mathbb {K}}_\mathrm {CLE}= \frac{1}{2}\big (\Sigma ^{\mathrm {fw}}(\Sigma ^{\mathrm {fw}})^\mathsf {T}{+} \Sigma ^{\mathrm {bw}}(\Sigma ^{\mathrm {bw}})^\mathsf {T}\big ). \end{aligned}$$

Here the diffusion matrix $\widehat{\mathbb {K}}_\mathrm {CLE}$ can be written in the explicit form

1.6 $$\begin{aligned} \widehat{\mathbb {K}}_\mathrm {CLE}({\varvec{c}})=\sum _{r=1}^R{\kappa ^r}\, \frac{1}{2} \Big ( \frac{{\varvec{c}}^{{\varvec{\alpha }}^r}}{{\varvec{c}}_*^{{\varvec{\alpha }}^r}} {+} \frac{{\varvec{c}}^{{\varvec{\beta }}^r}}{{\varvec{c}}_*^{{\varvec{\beta }}^r} } \Big )\, \big ({\varvec{\alpha }}^r{-}{\varvec{\beta }}^r\big ){\otimes }\big ({\varvec{\alpha }}^r{-}{\varvec{\beta }}^r\big ) \end{aligned}$$

that is different from ${\mathbb {K}}({\varvec{c}})$, because in the former the arithmetic mean while in the latter the logarithmic mean is taken.

One drawback of the chemical Langevin Eq. (1.5) is that it cannot be written as gradient flow of the relative entropy, as the Einstein relation for the drift flux and the diffusion flux is not satisfied. Therefore we propose other approximations that stay inside the theory of gradient flows and seem to work sufficiently well if the concentrations are not too large or small. Our simplest approximation is given by the gradient system $({\mathscr {P}}({\varvec{C}}), \widetilde{\varvec{E}}_V, {\varvec{K}})$ with

$$\begin{aligned} \widetilde{\varvec{E}}_V(\varrho ) = \int _{\varvec{C}}\Big ( \frac{1}{V} \rho \log \rho \,+\, \rho E \Big ) \;\!\mathrm {d}{\varvec{c}}, \ \text { where } \varrho = \rho \;\!\mathrm {d}{\varvec{c}}, \end{aligned}$$

which leads to the linear ***Fokker–Planck equation***

FPE $$\begin{aligned} {\dot{\rho }} = {\mathrm {div}}\Big ( \frac{1}{V} {\mathbb {K}}({\varvec{c}}) \nabla \rho + \rho {\varvec{R}}\Big ). \end{aligned}$$

In Sect. 5 we show that by systematically deriving higher-order corrections to $\widetilde{\varvec{E}}_V$ and ${\varvec{K}}$ we can recover the asymptotically correct diffusion matrix $\widehat{\mathbb {K}}_\text {CLE}$ while keeping the gradient structure, but have to accept several additional terms, or switch over to the notion of *asymptotic gradient flow structures* in the sense of [6].

### Hybrid modeling using gradient structures

A major advantage of the gradient flow description is that the different structures can be combined to obtain hybrid models, in which the set of chemical species is divided into subclasses which may be treated differently depending on the desired or needed accuracy. Our approach is based on the idea of model reduction for gradient structures. The idea is to approximate a complicated gradient structure $({\mathsf {X}},{\mathsf {E}}_X,{\mathsf {K}}_X)$ by a simpler one $({\mathsf {Y}},{\mathsf {E}}_Y,{\mathsf {K}}_Y)$ via an embedding mapping ${\mathsf {x}}= \Phi ({\mathsf {y}})$. Staying within the class of gradient systems has the advantage that the most important features of the original system can be preserved. In particular, decay of the driving functional along the approximate flow holds automatically. By contrast, such crucial features could get lost in a direct approach based on the evolution equation itself.

In Sect. 6 we shall deal with three examples for hybrid models where it is essential to keep *V* as a large but finite parameter. First, we shall consider a hybrid model in which an RRE is coupled to a Fokker–Planck equation. Here the set of species is divided into two classes: ${\varvec{C}}= {\varvec{C}}_{\mathrm {s}}{\times }{\varvec{C}}_{\mathrm {m}}$. Some of them will be described *stochastically* (s), while others are described *macroscopically* (m). This leads to a gradient flow structure on the hybrid state space ${\mathsf {Y}}={\mathscr {P}}({\varvec{C}}_{\mathrm {s}}){\times }{\varvec{C}}_{\mathrm {m}}$. The resulting gradient flow equation turns out to be a mean-field equation, in which the density of the component ${\varvec{c}}_{\mathrm {s}}$ satisfies a linear equation which is nonlinearly coupled to an ODE for the component ${\varvec{c}}_{\mathrm {m}}$.

We also study the coupling of an RRE for macroscopic variables to a CME for *n* microscopic variables. This leads to a hybrid system on ${\mathscr {P}}({\mathbb {N}}_0^n){\times }{{\varvec{C}}_{\mathrm {m}}}$. Finally we analyze a mixed CME / Fokker–Planck model with state space ${\mathscr {P}}({\mathfrak {N}})$, in which the underlying space ${\mathfrak {N}}:=\{0,1,\ldots ,N{-}1\}\cup {[N/V,\infty [}$ contains a mixture of discrete and continuous components.

The present work concentrates solely on the analytical underpinnings of hybrid modeling for CME; for numerical approaches to CME and to spatio-temporal CME we refer to [4, 12, 15, 27–29, 48, 61]. Geodesic convexity properties for the gradient structures appearing in this paper are studied in [38].

**Notational conventions** Throughout the paper we will consistently use the following notation to distinguish the different modeling levels.

- **Reaction-rate equation** The RRE is denoted by $({\varvec{C}},E,{\mathbb {K}})$:
- state and state space ${\varvec{c}}\in {\varvec{C}}:={[0,\infty [}^I$, steady state ${\varvec{c}}^*={\varvec{c}}_*$, dual variable ${\varvec{\zeta }}$
- energy functional $E({\varvec{c}})$, Onsager operator ${\mathbb {K}}({\varvec{c}})$
- conserved quantities ${\mathbb {Q}}{\varvec{c}}= {\varvec{q}}$, stoichiometric subsets ${\varvec{I}}({\varvec{q}})= \{\, {\varvec{c}}\in {\varvec{C}} \, | \, {\mathbb {Q}}{\varvec{c}}= {\varvec{q}} \,\}$.
- **Chemical master equation** The CME is denoted by $({\mathscr {P}}({\mathcal {N}}),{\mathcal {E}}_V,{\mathcal {K}}_V)$:
- state and state space ${\varvec{u}}=(u_{\varvec{n}})_{{\varvec{n}}\in {\mathcal {N}}} \in {\mathscr {P}}({\mathcal {N}})\subset$, steady state ${\varvec{w}}^V$, dual variable ${\varvec{\mu }}$
- energy functional ${\mathcal {E}}_V({\varvec{u}})$, Onsager operator ${\mathcal {K}}_V({\varvec{u}})$
- invariant subsets $${\mathcal {I}}(\overline{{\varvec{n}}})= \{\, {\varvec{n}}\in {\mathcal {N}} \, | \, {\mathbb {Q}}{\varvec{n}}= {\mathbb {Q}}\overline{{\varvec{n}}} \,\} $$.
- **Liouville equation** The LE is denoted by $({\mathscr {P}}({\varvec{C}}),{\varvec{E}},{\varvec{K}})$:
- state and state space $\varrho = \rho \;\!\mathrm {d}{\varvec{c}}\in {\mathscr {P}}({\varvec{C}})$, steady state $\delta _{{\varvec{c}}_*}$, dual variable $\xi$
- energy functional ${\varvec{E}}(\varrho ) = \int _{\varvec{C}}E({\varvec{c}}) \;\!\mathrm {d}\varrho ({\varvec{c}})$, Onsager operator ${\varvec{K}}(\varrho )=-{\mathrm {div}}\big ( \varrho (\cdot ) {\mathbb {K}}(\cdot )\nabla \Box \big )$
- **Fokker–Planck equation** The FPE is denoted by $({\mathscr {P}}({\varvec{C}}), \widetilde{\varvec{E}}_V ,{\varvec{K}})$:
- state and state space $\varrho \in {\mathcal {P}}({\varvec{C}}):={\mathscr {P}}({\varvec{C}})$, steady state $W_V$, dual variable $\xi$
- energy functional $\widetilde{\varvec{E}}_V(\varrho ) = \int _{\varvec{C}}\big (\frac{1}{V} \rho ({\varvec{c}})\log \rho ({\varvec{c}}) {+}\rho ({\varvec{c}}) E({\varvec{c}}) \big )\;\!\mathrm {d}{\varvec{c}}$, Onsager operator ${\varvec{K}}$.
- **Hybrid systems** are denoted by “mathfrak” letters:
- $({\mathscr {P}}({\varvec{C}}_{\mathrm {s}}){\times }{\varvec{C}}_{\mathrm {m}}, {\mathfrak {E}}^\text {FP-RR}_V, {\mathfrak {K}}^\text {FP-RR}_V)$ for coupling FPE and RRE
- $({\mathscr {P}}({\mathbb {N}}_0^{J}){\times }{\varvec{C}}_{\mathrm {m}}, , {\mathfrak {E}}^\text {CM-RR}_V, {\mathfrak {K}}^\text {CM-RR}_V)$ for coupling CME and RRE
- $({\mathscr {P}}({\mathfrak {N}}_{V,N}), {\mathfrak {E}}_{V,N},{\mathfrak {K}}_{V,N})$ for merging discrete and continuous modeling for one species.

The space of all signed Borel measures of bounded variation on ${\varvec{C}}$ is denoted by ${\mathscr {M}}({\varvec{C}})$.

## Reaction Rate Equations

We denote by ${\varvec{c}}=(c_1,\ldots ,c_I)\in {\varvec{C}}:= [ 0, \infty [^I$ the concentrations of *I* different chemical species $X_1,\ldots , X_I$ reacting according to the mass action law, i.e., the reactions2.1for $r=1,\ldots ,R$, where *R* is the number of possible reactions, ${\varvec{\alpha }}^r,{\varvec{\beta }}^r\in {\mathbb {N}}_0^I$ are the vectors of the stoichiometric coefficients, and $k^r_{\mathrm {fw}}, k^r_{\mathrm {bw}}> 0$ are the forward and backward reaction-rates. In general these rates may depend on ${\varvec{c}}$, but for simplicity we keep them as constants in this work. A typical example is the splitting of water into hydrogen and oxygen, namely .

The corresponding *reaction-rate equations* (RRE) are given via the ODE system

2.2 $$\begin{aligned} {\dot{{\varvec{c}}}} = -{\varvec{R}}({\varvec{c}}) \quad \text {with }{\varvec{R}}({\varvec{c}}):=\sum _{r=1}^R \big (k^r_{\mathrm {fw}}{\varvec{c}}^{{\varvec{\alpha }}^r} - k^r_{\mathrm {bw}}{\varvec{c}}^{{\varvec{\beta }}^r}\big )\,\big ( {\varvec{\alpha }}^r {-} {\varvec{\beta }}^r\big ), \end{aligned}$$

where $${\varvec{c}}^{\varvec{\alpha }}=c_1^{\alpha _1}\cdots c_I^{\alpha _I}$$, see [17, 19, 26].

### Stoichiometry, Conservation, and Decomposition of the State Space

The stoichiometric subspace ${\mathbb {S}}\subset {\mathbb {R}}^I$ and its orthogonal complement ${\mathbb {S}}^\perp$ are defined via

2.3 $$\begin{aligned} {\mathbb {S}}:=\mathrm {span} \{\, {\varvec{\alpha }}^r - {\varvec{\beta }}^r \, | \, r=1,\ldots ,R \,\} , \ \ \ {\mathbb {S}}^\perp := \{\, {\varvec{\xi }}\in {\mathbb {R}}^I \, | \, {\varvec{\xi }}{\cdot }{\varvec{\mu }}=0\text { for all }{\varvec{\mu }}\in {\mathbb {S}} \,\} . \end{aligned}$$

For each ${\varvec{\xi }}\in {\mathbb {S}}^\perp$ the function $C_{\varvec{\xi }}({\varvec{c}})={\varvec{\xi }}\cdot {\varvec{c}}$ defines a first integral, which easily follows from ${\varvec{\xi }}\cdot {\varvec{R}}({\varvec{c}})\equiv 0$. These conservation laws often go under the name *conservation of atomic species*, see [17]. Suppose now that ${\mathbb {S}}^\perp$ is a non-trivial subspace of ${\mathbb {R}}^I$. We shall argue that the RRE induces a decomposition of the state space ${\varvec{C}}={[0,\infty [}^I$ into affine invariant subsets. (If ${\mathbb {S}}^\perp = \{ \mathbf{0}\}$, the only invariant set is ${\varvec{C}}$ itself.)

Choosing a basis $\{\, {\varvec{m}}_k \in {\mathbb {R}}^I \, | \, k=1, \ldots ,m_{\mathbb {W}} \,\}$ of ${\mathbb {S}}^\bot$ we define the matrix ${\mathbb {Q}}\in {\mathbb {R}}^{m_{\mathbb {W}}{\times }I}$, which has the rows ${\varvec{m}}_k \in {\mathbb {R}}^I$. By construction we have ${\mathbb {Q}}[{\mathbb {S}}]=\{{\mathbf{0}}\}$, and we conclude that the solutions ${\varvec{c}}$ of (2.2) conserve ${\mathbb {Q}}{\varvec{c}}$ as follows:

2.4 $$\begin{aligned} {\dot{{\varvec{c}}}}= - {\varvec{R}}({\varvec{c}}) \quad \Longrightarrow \quad {\mathbb {Q}}{\varvec{c}}(t) = {\mathbb {Q}}{\varvec{c}}(0) \text { for }t>0. \end{aligned}$$

By construction every affine conserved quantity is of the form ${\varvec{\xi }}\cdot {\varvec{c}}+ q$ for some ${\varvec{\xi }}\in {\mathbb {S}}^\perp$ and $q \in {\mathbb {R}}$. This allows us to decompose the full state space ${\varvec{C}}={[0,\infty [}^I$ into the invariant, affine subsets $({\varvec{c}}_0{+}{\mathbb {S}})\cap {\varvec{C}}$ for ${\varvec{c}}_0 \in {\varvec{C}}$. Using the notation

$$\begin{aligned} {\mathfrak {Q}}:= \{\, {\mathbb {Q}}{\varvec{c}}\in {\mathbb {R}}^{m_{\mathbb {W}}} \, | \, {\varvec{c}}\in {\varvec{C}} \,\} \end{aligned}$$

we define, for all ${\varvec{q}}\in {\mathfrak {Q}}$, the sets

2.5 $$\begin{aligned} {\varvec{I}}({\varvec{q}}):= \{\, {\varvec{c}}\in {\varvec{C}} \, | \, {\mathbb {Q}}{\varvec{c}}={\varvec{q}} \,\} . \end{aligned}$$

Then, ${\varvec{q}}_1\ne {\varvec{q}}_2$ implies ${\varvec{I}}({\varvec{q}}_1)\cap {\varvec{I}}({\varvec{q}}_2)=\emptyset$, and we have ${\varvec{C}}=\bigcup _{{\varvec{q}}\in {\mathfrak {Q}}} {\varvec{I}}({\varvec{q}})$. Let us note that this decomposition does not depend on the choice of the orthonormal basis which determines the matrix ${\mathbb {Q}}$, although the set ${\varvec{I}}({\varvec{q}})$ does depend on ${\mathbb {Q}}$. Note also that we can always write ${\varvec{I}}({\varvec{q}}) = ({\varvec{c}}{+}{\mathbb {S}})\cap {\varvec{C}}$ for some arbitrary ${\varvec{c}}\in {\varvec{C}}$ satisfying ${\mathbb {Q}}{\varvec{c}}= {\varvec{q}}$.

### Detailed Balance and the Wegscheider Matrix

We say that the above reaction system fulfills the *condition of detailed balance* if there exists a positive equilibrium density vector ${\varvec{c}}_* \in ]0,\infty [^I$ such that all reactions are simultaneously in equilibrium, i.e.,

2.6 $$\begin{aligned} \kappa ^r_*:=k^r_{\mathrm {fw}}{\varvec{c}}_*^{{\varvec{\alpha }}^r} = k_{\mathrm {bw}}^r {\varvec{c}}_*^{{\varvec{\beta }}^r} \ \text { for } \ r=1,\dots ,R. \end{aligned}$$

This condition implies that ${\varvec{R}}({\varvec{c}}_*)=0$, but we emphasize that this condition is stronger in general cases. The condition of detailed balance is also called the condition of *microscopic reversibility*, see [17, p. 45] or [10] for a general discussion of these concepts.

We are looking for a characterization of detailed balance. Let ${\mathbb {W}}\in {\mathbb {Z}}^{R\times I}$ be the matrix which has the row vectors ${\varvec{\gamma }}^r:={\varvec{\alpha }}^r {-} {{\varvec{\beta }}^r} \in {\mathbb {Z}}^I$, $r=1, \dots , R$. We call ${\mathbb {W}}$ the *Wegscheider matrix* because of the pioneering work in [60]. We then have

$$\begin{aligned} {\mathbb {S}}= {{\,\mathrm{Ran}\,}}{\mathbb {W}}^\mathsf {T}\quad \text {and} \quad {\mathbb {S}}^\bot = {{\,\mathrm{Ker}\,}}{\mathbb {W}}, \end{aligned}$$

which explains the abbreviation $m_{\mathbb {W}}:= \dim {\mathbb {S}}^\bot =\dim {{\,\mathrm{Ker}\,}}{\mathbb {W}}$. Since ${\varvec{c}}_*$ is strictly positive, we can take the logarithm of the polynomial conditions (2.6) and find the equivalent linear system

2.7 $$\begin{aligned} {\mathbb {W}}\,{\mathbf {log}}\,{\varvec{c}}_* = \big (\log (k_{\mathrm {bw}}^r/k^r_{\mathrm {fw}})\big )_{r=1,\dots ,R}, \quad \text {where } {\mathbf {log}}\,{\varvec{c}}= \big ( \log c_i\big )_{i=1,\ldots ,I}. \end{aligned}$$

By Fredholm’s alternative, (2.7) is solvable if and only if

2.8 $$\begin{aligned} {\varvec{y}}\cdot \big (\log (k_{\mathrm {bw}}^r/k^r_{\mathrm {fw}})\big )_{r=1,\dots ,R} =0\quad \text {for all }{\varvec{y}}\in {{\,\mathrm{Ker}\,}}{\mathbb {W}}^\mathsf {T}. \end{aligned}$$

These conditions on the reaction coefficients $k^r_{\mathrm {fw}}$ and $k^r_{\mathrm {bw}}$ are called *Wegscheider conditions* (see, e.g., [25, 57, 59, 60]). By choosing a basis of ${{\,\mathrm{Ker}\,}}{\mathbb {W}}^\mathsf {T}$ and exponentiation they can be rewritten as polynomial conditions without referring to the equilibrium state ${\varvec{c}}_*$.

Let $n_{\mathbb {W}}:= \dim ({{\,\mathrm{Ker}\,}}{\mathbb {W}}^\mathsf {T}) \in {\mathbb {N}}_0$ denote the number of Wegscheider conditions. Then the following assertions hold:

(i) If the stoichiometric vectors ${\varvec{\alpha }}^r-{\varvec{\beta }}^r$, $r=1,\dots ,R$, are linearly independent, then ${{\,\mathrm{Ker}\,}}{\mathbb {W}}^\mathsf {T}= \{ \mathbf{0} \}$, hence there is no Wegscheider condition.
(ii) If ${\varvec{\alpha }}^r-{\varvec{\beta }}^r$, $r=1,\dots ,R$, are linearly dependent, then $\dim ({{\,\mathrm{Ker}\,}}{\mathbb {W}}^\mathsf {T})>0$ and non-trivial Wegscheider conditions appear.

Since $\dim ({{\,\mathrm{Ran}\,}}{\mathbb {W}}) = \dim ({{\,\mathrm{Ran}\,}}{\mathbb {W}}^\mathsf {T}) = \dim {\mathbb {S}}$ by standard linear algebra, the number of Wegscheider conditions can be expressed as

$$\begin{aligned} n_{\mathbb {W}}= R - \dim {\mathbb {S}}= R - I + \dim ({{\,\mathrm{Ker}\,}}{\mathbb {W}}) = R - I + m_{\mathbb {W}}. \end{aligned}$$

Hence, if the number *R* of reactions is smaller than the number *I* of species, the Wegscheider conditions can usually be satisfied easily.

#### Remark 2.1

(*Wellposedness of RRE*) We conclude this subsection with a statement concerning the well-posedness of the RRE given as in Theorem 2.2 below. For all ${\varvec{c}}(0)\in {\varvec{C}}={[0,\infty [}^I$ there exists a unique global solution ${\varvec{c}}: {[0,\infty [} \rightarrow {\varvec{C}}$. Local existence for solutions starting in the interior of ${\varvec{C}}$ is trivial, as ${\varvec{R}}$ is a polynomial vector field. Since the relative entropy *E* is a coercive Liapunov functional, the solutions cannot blow up and stay inside a region $B_R(0) \cap {\varvec{C}}$ for some $R>0$.

Moreover, solutions cannot leave this region via the boundary $\partial {\varvec{C}}$, since the vector field is either tangential to $\partial {\varvec{C}}$ or points inwards. Indeed, if $c_j(t_0)=0$ for some *j*, then

$$\begin{aligned} {\dot{c}}_j(t_0)=- R_j({\varvec{c}}(t_0)) = -\sum _{r=1}^R \kappa _*^r \big (\tfrac{{\varvec{c}}^{{\varvec{\alpha }}^r}(t_0)}{{\varvec{c}}_*^{{\varvec{\alpha }}^r}} - \tfrac{{\varvec{c}}^{{\varvec{\beta }}^r}(t_0)}{{\varvec{c}}^{{\varvec{\beta }}^r}_*} \big ) \big (\alpha ^r_j {-} \beta ^r_j\big )\ge 0, \end{aligned}$$

because each term in the sum is nonpositive: If $\alpha ^r_j = \beta ^r_j$ or $\min \{\alpha ^r_j ,\beta ^r_j \} >0$, then the term is 0. Thus, we are left with the cases $(\alpha ^r_j,\beta ^r_j) \in \{ (n,0),(0,n)\}$ for some positive *n*. In the first case $c_j(t_0)=0$ implies ${\varvec{c}}^{{\varvec{\alpha }}^r}(t_0)=0$ and the result follows, and the second case is similar.

### The Reaction-Rate Equations as a Gradient System

We show that a RRE satisfying the detailed-balance condition can be generated by a gradient system $({\varvec{C}},E,{\mathbb {K}})$. Here, the state space ${\varvec{C}}:= {[0,\infty [}^I$ contains all possible concentration vectors ${\varvec{c}}$. The driving functional is the relative entropy $E:{\varvec{C}}\rightarrow {[0,\infty [}$ and the Onsager matrix ${\mathbb {K}}$ is chosen suitably (recall that $\lambda _{\mathrm {B}}(z)=z \log z - z +1 \ge 0$):

2.9 $$\begin{aligned} E({\varvec{c}}) := \sum _{i=1}^I \lambda _{\mathrm {B}}\bigg (\frac{c_i}{c^*_i}\bigg ) c^*_i \ \text { and } \ {\mathbb {K}}({\varvec{c}})= \sum _{r=1}^R \kappa ^r_* \Lambda \bigg (\frac{{\varvec{c}}^{{\varvec{\alpha }}^r}}{{\varvec{c}}_*^{{\varvec{\alpha }}^r}}, \frac{{\varvec{c}}^{{\varvec{\beta }}^r}}{{\varvec{c}}^{{\varvec{\beta }}^r}_*}\bigg )\, \big ({\varvec{\alpha }}^r {-} {\varvec{\beta }}^r\big ) \otimes \big ({\varvec{\alpha }}^r {-} {\varvec{\beta }}^r\big ), \end{aligned}$$

where the logarithmic-mean function $\Lambda$ is given via

2.10 $$\begin{aligned} \Lambda (a,b)= \int _0^1 a^s b^{1-s} \;\!\mathrm {d}s = \frac{a - b}{\log a - \log b}. \end{aligned}$$

The following result shows that a RRE (2.2) satisfying the detailed-balance condition (2.6) is indeed generated by the gradient system $({\varvec{C}},E,{\mathbb {K}})$. This was first established in [62, Sect. VII] to derive entropy bounds for hyperbolic conservation laws in reactive flows and was rederived in [40] in the context of reaction diffusion systems including electric charge-interactions. It is interesting to note that for continuous time Markov chains (CTMC), which form a special subclass of RRE with linear reactions, there are several distinct gradient structures, see [37, Prop. 4.2] and [42, Thm. 3.1] and Sect. 2.4. However, in the case of nonlinear reactions according to the mass-action law, only the gradient structure with the Boltzmann entropy remains. The key fact is the logarithm identity $({\varvec{\alpha }}- {\varvec{\beta }}) \cdot {\mathbf {log}}\,{\varvec{c}}= \log ({\varvec{c}}^{{\varvec{\alpha }}- {\varvec{\beta }}})$.

#### Theorem 2.2

(Gradient structure for RRE) If the RRE (2.2) satisfies the detailed-balance condition (2.6) for a positive steady state ${\varvec{c}}_*=(c^*_i)_{i=1,\ldots ,I}$, then it has the gradient structure $({\varvec{C}},E,{\mathbb {K}})$ defined in (2.9), namely ${\dot{{\varvec{c}}}}= - {\varvec{R}}({\varvec{c}})=- {\mathbb {K}}({\varvec{c}}) {\mathrm {D}}E({\varvec{c}})$.

#### Proof

Multiplying ${\mathrm {D}}E({\varvec{c}})= (\log (c_i/c^*_i))_{i=1,\ldots ,I}$ by ${\varvec{\alpha }}^r{-}{\varvec{\beta }}^r \in {\mathbb {R}}^I$ we obtain

2.11 $$\begin{aligned} \begin{aligned} (\log (c_i/c^*_i))_{i=1,\ldots ,I} \cdot \big ({\varvec{\alpha }}^r {-} {\varvec{\beta }}^r\big )&= \sum _{i=1}^I \Big ( \alpha _i^r \log (c_i/c^*_i) - \beta _i^r \log (c_i/c^*_i)\Big ) \\&= \log \bigg (\tfrac{{\varvec{c}}^{{\varvec{\alpha }}^r}}{{\varvec{c}}_*^{{\varvec{\alpha }}^r}}\bigg ) - \log \bigg ( \tfrac{{\varvec{c}}^{{\varvec{\beta }}^r}}{{\varvec{c}}^{{\varvec{\beta }}^r}_*} \bigg ) , \end{aligned} \end{aligned}$$

which is the denominator of $\Lambda \bigg (\tfrac{{\varvec{c}}^{{\varvec{\alpha }}^r}}{{\varvec{c}}_*^{{\varvec{\alpha }}^r}}, \tfrac{{\varvec{c}}^{{\varvec{\beta }}^r}}{{\varvec{c}}^{{\varvec{\beta }}^r}_*}\bigg )$. Hence, using $\Lambda (a,b)(\log a {-}\log b) = a{-}b$ gives

$$\begin{aligned} {\mathbb {K}}({\varvec{c}}){\mathrm {D}}E({\varvec{c}})=\sum _{r=1}^R \kappa _*^r \bigg (\tfrac{{\varvec{c}}^{{\varvec{\alpha }}^r}}{{\varvec{c}}_*^{{\varvec{\alpha }}^r}} - \tfrac{{\varvec{c}}^{{\varvec{\beta }}^r}}{{\varvec{c}}^{{\varvec{\beta }}^r}_*} \bigg ) \big ({\varvec{\alpha }}^r {-} {\varvec{\beta }}^r\big ) \overset{{\mathrm {D}}{\mathrm {B}}}{=}\sum _{r=1}^R \big (k^r_{\mathrm {fw}}{\varvec{c}}^{{\varvec{\alpha }}^r} {-} k^r_{\mathrm {bw}}{\varvec{c}}^{{\varvec{\beta }}^r}\big )\, \big ( {\varvec{\alpha }}^r {-} {\varvec{\beta }}^r\big ) = {\varvec{R}}({\varvec{c}}), \end{aligned}$$

where we used the detailed-balance condition (2.6) in $\overset{{\mathrm {D}}{\mathrm {B}}}{=}$. Thus, the assertion is established. $\square$

Summarizing the above derivations, we have rewritten the RRE in thermodynamic form

2.12 $$\begin{aligned} {\dot{{\varvec{c}}}} = - {\varvec{R}}({\varvec{c}})= - {\mathbb {K}}({\varvec{c}}) {\varvec{\mu }}\quad \text {with }\ {\varvec{\mu }}= {\mathrm {D}}E({\varvec{c}}), \end{aligned}$$

which is also called the Onsager principle [51, 52]. The latter states that the rate (flux) of a macroscopic variable is given as the product of a symmetric positive definite matrix ${\mathbb {K}}$ and the thermodynamic driving force $-{\varvec{\mu }}$, see e.g. [10, Ch. X, § 4]. The symmetry ${\mathbb {K}}= {\mathbb {K}}^\top$ is related to microscopic reversibility, i.e., detailed balance, see also [44, 45]. Subsequently, we refer to ${\mathbb {K}}$ as the Onsager operator or matrix.

Here we clearly see the advantage of using the Onsager operator ${\mathbb {K}}$ to write the RRE as a gradient system,as opposed to working with the Riemannian tensor: we do not have to take care of the fact that ${\mathbb {K}}$ is not invertible except if ${\mathbb {S}}={\mathbb {R}}^I$.

### Continuous Time Markov Chains as a Gradient System

The forward equation for a reversible CTMC on a discrete space $\{1,2,\ldots ,I\}$ is a special case of the RRE considered above. In this case all reactions are of the formand the reaction rates $k^{ij}_{\mathrm {fw}}$ (resp. $k^{ij}_{\mathrm {bw}}$) are interpreted as the transition rates from *i* to *j* (resp. from *j* to *i*). The reaction-rate equation is the linear system of ODEs

2.13 $$\begin{aligned} {\dot{{\varvec{c}}}} = - {\varvec{R}}({\varvec{c}}) = {\mathcal {A}}{\varvec{c}}\quad \text {with } {\mathcal {A}}{\varvec{c}}= -\sum _{i<j} \big (k^{ij}_{\mathrm {fw}}c_i - k^{ij}_{\mathrm {bw}}c_j\big ) ({\varvec{e}}_i{-}{\varvec{e}}_j), \end{aligned}$$

and the detailed-balance condition for the equilibrium state ${\varvec{c}}_*$ takes the form

2.14 $$\begin{aligned} \kappa _*^{ij} := c_i^* k^{ij}_{\mathrm {fw}}= c_j^* k^{ij}_{\mathrm {bw}}\ \text { for } \ 1 \le i < j \le I. \end{aligned}$$

Using this condition, the RRE can be written coordinate-wise as

$$\begin{aligned} \frac{{\dot{c}}_i}{c_i^*} = \sum _{j < i} k^{ji}_{\mathrm {bw}}\Big (\frac{c_j}{c_j^*} - \frac{c_i}{c_i^*}\Big ) + \sum _{j > i} k^{ij}_{\mathrm {fw}}\Big (\frac{c_j}{c_j^*} - \frac{c_i}{c_i^*}\Big ), \ \text { or equiv., } \ {{\dot{c}}_i} = \sum _{j \ne i} \kappa _*^{ij} \Big (\frac{c_j}{c_j^*} - \frac{c_i}{c_i^*}\Big ). \end{aligned}$$

Here we used the notational convention that $\kappa _*^{ij} := \kappa _*^{ji}$ for $j < i$. The relative entropy *E* is as above and the Onsager matrix takes the form

2.15 $$\begin{aligned} {\mathbb {K}}_{\mathrm {M}}({\varvec{c}})= \sum _{i<j} \kappa _*^{ij} \Lambda \Big (\frac{c_i}{c_i^*} , \frac{c_j}{c_j^*}\Big ) ({\varvec{e}}_i{-}{\varvec{e}}_j) \otimes ({\varvec{e}}_i{-}{\varvec{e}}_j), \end{aligned}$$

where ${\varvec{e}}_i\in {\mathbb {R}}^I$ denotes the *i*-th unit vector. We then have the gradient structure $({\varvec{C}},E, {\mathbb {K}}_{\mathrm{M}})$, namely

$$\begin{aligned} {\dot{{\varvec{c}}}} = {\mathcal {A}}{\varvec{c}}=- {\mathbb {K}}_{\mathrm{M}}({\varvec{c}}) {\mathrm {D}}E({\varvec{c}}). \end{aligned}$$

This gradient flow structure has been found in the independent works [37] (which deals with Markov chains exclusively) and [40] (in the setting of reaction-diffusion systems, in which Markov chains are implicitly contained). The related work [7] deals with discretizations of Fokker–Plank equations.

In fact, for the construction of gradient structures for Markov chains ${\dot{{\varvec{c}}}} = {\mathcal {A}}{\varvec{c}}$ we do not need the summation rule for logarithms. Hence, following [37, 42] there are more general gradient structures. Choosing a strictly convex function $\phi :[0,\infty [ \rightarrow {\mathbb {R}}$ that is smooth on $]0,\infty [$ we set

2.16 $$\begin{aligned} E^\phi ({\varvec{c}}) := \sum _{i=1}^n c_{i}^* \,\phi \Big ( \frac{c_i}{c_i^*}\Big ), \quad {\mathbb {K}}_{\mathrm{M}}^\phi ({\varvec{c}}) = \sum _{j=2}^n \sum _{i=1}^{j-1} \kappa _*^{ij} \,\Phi \Big ( \frac{c_i}{c_i^*}\,,\, \frac{c_j}{c_j^*}\Big ) \, ({\varvec{e}}_i{-}{\varvec{e}}_j) \otimes ({\varvec{e}}_i{-}{\varvec{e}}_j), \end{aligned}$$

where $\Phi ( a,b)=(a-b)/(\phi '(a){-}\phi '(b))$ for $0<a\ne b$ and $\Phi (a,a)=1/\phi ''(a)$. The gradient flow structure $({\varvec{C}},E, {\mathbb {K}}_{\mathrm{M}})$ corresponds to the case where $\phi = \lambda _{\mathrm{B}}: z \mapsto z \log z - z +1$.

#### Proposition 2.3

(Gradient structure for CTMC) If the CTMC (2.13) satisfies the detailed-balance condition (2.14) for a positive steady state ${\varvec{c}}_*=(c^*_i)_{i=1,\ldots ,I}$, then it has the gradient structures $({\varvec{C}},E^\phi , K_{\mathrm {M}}^\phi )$, namely ${\dot{{\varvec{c}}}}= {\mathcal {A}}{\varvec{c}}=- K_{\mathrm {M}}^\phi ({\varvec{c}}) {\mathrm {D}}E^\phi ({\varvec{c}})$.

#### Remark 2.4

The construction in Proposition 2.3 does not extend to general RRE. There one would need to replace the quantity $\Lambda \bigg (\tfrac{{\varvec{c}}^{{\varvec{\alpha }}^r}}{{\varvec{c}}_*^{{\varvec{\alpha }}^r}}, \tfrac{{\varvec{c}}^{{\varvec{\beta }}^r}}{{\varvec{c}}^{{\varvec{\beta }}^r}_*}\bigg )$ in (2.9) by $\bigg (\tfrac{{\varvec{c}}^{{\varvec{\alpha }}^r}}{{\varvec{c}}_*^{{\varvec{\alpha }}^r}} - \tfrac{{\varvec{c}}^{{\varvec{\beta }}^r}}{{\varvec{c}}^{{\varvec{\beta }}^r}_*}\bigg )/\big ( ({\varvec{\alpha }}^r - {\varvec{\beta }}^r) \cdot {\varvec{\phi }}'\big (\frac{{\varvec{c}}}{{\varvec{c}}_{*}}\big )\big )$, but this quantity can be negative in general. As a consequence, the corresponding Onsager matrix would not be positive definite. This cannot happen for Markov chains (i.e., when ${\varvec{\alpha }}= {\varvec{e}}_i$ and ${\varvec{\beta }}= {\varvec{e}}_j$), by virtue of the convexity of $\phi$.

In the following we will mainly concentrate on the gradient structure $({\varvec{C}},E,{\mathbb {K}}_{\mathrm {M}})$ with the logarithmic entropy, as it is the only one that connects with the RRE.

### Generalized Gradient Structures

For Markov chains and RRE there are several families of *generalized gradient structures* $({\varvec{C}},E,\Psi ^*)$ where the quadratic function $\Psi ^*({\varvec{c}},{\varvec{\zeta }}) = \frac{1}{2}\langle {\varvec{\zeta }}, {\mathbb {K}}({\varvec{c}}){\varvec{\zeta }}\rangle$ is replaced by a general *dual dissipation potential* $\Psi ^*({\varvec{c}},\,\cdot \,): {\mathbb {R}}^I \rightarrow [ 0, \infty [$ that is continuous and convex and satisfies $\Psi ^*({\varvec{c}},0)=0$.

In the case of RRE, the monomial terms ${\varvec{c}}^{\varvec{\alpha }}$ can only be generated by the logarithmic summation rule $\sum _{i=1}^I\log (b_i) = \log \big ( \Pi _{i=1}^I b_i\big )$. Hence, we stick to the relative entropy *E* defined in (2.9), i.e., $\phi (z) = \lambda _{\mathrm {B}}(z)$. However, we may replace the linear Onsager principle ${\dot{{\varvec{c}}}} = - {\mathbb {K}}({\varvec{c}}) {\mathrm {D}}E({\varvec{c}})$ by the more general nonlinear form ${\dot{{\varvec{c}}}}= \partial _{\varvec{\zeta }}\Psi ^*\big ({\varvec{c}}, -{\mathrm {D}}E({\varvec{c}})\big )$.

To define $\Psi ^*$ we choose an arbitrary family of smooth dissipation functionals $\psi _r:{\mathbb {R}}\rightarrow {[0,\infty [}$, i.e., $\psi _r(0)=\psi '_r(0)=0$ and $\psi ''_r >0$ and define the dissipation potential

2.17 $$\begin{aligned} \Psi ^*({\varvec{c}},{\varvec{\zeta }}) = \sum _{r=1}^R L_r({\varvec{c}})\, \psi _r\big ( ({\varvec{\alpha }}^r {-} {\varvec{\beta }}^r)\cdot {\varvec{\zeta }}\big ) \ \text { with } L_r ({\varvec{c}})= \kappa ^r_*\,\frac{ \tfrac{{\varvec{c}}^{{\varvec{\beta }}^r}}{{\varvec{c}}^{{\varvec{\beta }}^r}_*} -\frac{{\varvec{c}}^{{\varvec{\alpha }}^r}}{{\varvec{c}}_*^{{\varvec{\alpha }}^r}} }{\psi '_r\bigg ( \log \tfrac{{\varvec{c}}^{{\varvec{\beta }}^r}}{{\varvec{c}}^{{\varvec{\beta }}^r}_*} - \log \frac{{\varvec{c}}^{{\varvec{\alpha }}^r}}{{\varvec{c}}_*^{{\varvec{\alpha }}^r}} \bigg )}.\nonumber \\ \end{aligned}$$

Using (2.11) we easily obtain $-{\varvec{R}}({\varvec{c}}) = \partial _{\varvec{\zeta }}\Psi ^*({\varvec{c}},- {\mathrm {D}}E({\varvec{c}}))$, i.e., ${\dot{{\varvec{c}}}} = -{\varvec{R}}({\varvec{c}})$ is generated by the generalized gradient system $({\varvec{C}},E,\Psi ^*)$.

The case $\psi _r(\zeta )=\frac{1}{2}\zeta ^2$ leads to the quadratic dissipation potential in (2.9), i.e., the functions $L_r$ are given in terms of the logarithmic mean. In [3] the choices $\psi _r(\pm \zeta ) = {\mathrm {e}}^\zeta -1-\zeta$ is used. Based on a derivation via the large deviation principle (see [44–46]) a special role is played by the choice of a “cosh-type” function $\psi _r$:

2.18 $$\begin{aligned} \psi _r(\zeta )={\mathsf {C}}^*(\zeta ):=4 \cosh \big (\frac{1}{2}\zeta \big )- 4 \quad \text {giving} \quad L_r({\varvec{c}}) = \kappa ^r_* \Big (\frac{{\varvec{c}}^{{\varvec{\alpha }}^r}}{{\varvec{c}}_*^{{\varvec{\alpha }}^r}}\, \frac{{\varvec{c}}^{{\varvec{\beta }}^r}}{{\varvec{c}}_*^{{\varvec{\beta }}^r}} \Big )^{1/2}. \end{aligned}$$

Here ${\mathsf {C}}^*$ is normalized such that ${\mathsf {C}}^*(\zeta )=\frac{1}{2}\zeta ^2 + O(\zeta ^4)$. Hence, the dual dissipation potential takes the form

2.19 $$\begin{aligned} \Psi ^*_{\cosh }({\varvec{c}}, {\varvec{\zeta }}) := \sum _{r=1}^R \kappa ^r_* \,\Big (\frac{{\varvec{c}}^{{\varvec{\alpha }}^r}}{{\varvec{c}}_*^{{\varvec{\alpha }}^r}}\, \frac{{\varvec{c}}^{{\varvec{\beta }}^r}}{{\varvec{c}}_*^{{\varvec{\beta }}^r}} \Big )^{1/2} \, {\mathsf {C}}^*\big ( ({\varvec{\alpha }}^r{-} {\varvec{\beta }}^r)\varvec{\cdot }{\varvec{\zeta }}\big ). \end{aligned}$$

It is shown in [43, Prop. 4.1] that this generalized gradient structure is distinguished as the only tilt-invariant gradient structure for CTMCs. The tilt-invariance even extends to nonlinear RRE, see [47].

## The Chemical Master Equation

### Modeling Discrete Particle Numbers via CME

The chemical master equation (CME) is a CTMC that is defined on the set ${\mathcal {N}}={\mathbb {N}}_0^I$ where ${\varvec{n}}=(n_1,\ldots ,n_I) \in {\mathcal {N}}$ is the vector of particle numbers, see [39] for an introduction. This means that $n_i\in {\mathbb {N}}_0$ denotes the number of particles of species $X_i$ in a sufficiently big volume, whose size is denoted by $V>0$. The modeling assumes that all particles move randomly in this big volume (well-stirred tank reactor) so that they can meet independently. The dynamics is formulated in terms of the probabilities

$$\begin{aligned} u_{\varvec{n}}(t) = \text {probability that at time }t\text { there are }n_i\text { particles of species }X_i\text { for }i=1,\ldots ,I. \end{aligned}$$

All the *R* reaction pairs may happen independently of each other according to the number of the available atoms needed for the reactions and the reaction coefficients $k^r_{\mathrm {fw}}\ge 0$ and $k^r_{\mathrm {bw}}\ge 0$, respectively. Moreover, the jump intensities

$$\begin{aligned} k^r_{\mathrm {fw}}\!\;{\mathbb {B}}_{V}^{{\varvec{\alpha }}^r}\!({\varvec{n}})\!\; \text { from }{\varvec{n}}+ {\varvec{\alpha }}^r \text { to }{\varvec{n}}+ {\varvec{\beta }}^r \quad \text { and } \quad k^r_{\mathrm {bw}}\!\;{\mathbb {B}}_{V}^{{\varvec{\beta }}^r}\!({\varvec{n}})\!\; \text { from }{\varvec{n}}+ {\varvec{\beta }}^r\text { to }{\varvec{n}}+ {\varvec{\alpha }}^r \end{aligned}$$

also depend on the volume *V*, as $n_i$ denotes the absolute particle number, while for the reaction the densities $c_i=n_i/V$ matter. The specific form of $\;{\mathbb {B}}_{V}^{{\varvec{\alpha }}}\!({\varvec{n}})\!\;$ (cf. [32, 39]) reads

3.1 $$\begin{aligned} \!\;{\mathbb {B}}_{V}^{{\varvec{\alpha }}}\!({\varvec{n}})\!\; = \left\{ \begin{array}{cl} \displaystyle \frac{V ({\varvec{n}}+{\varvec{\alpha }})!}{V^{|{\varvec{\alpha }}|}{\varvec{n}}!} &{} \text {for } {\varvec{n}}\in {\mathcal {N}}, \\ 0 &{} \text {for } {\varvec{n}}\not \in {\mathcal {N}}, \end{array}\right. \quad \text {where } {\varvec{n}}! = \prod _{i=1}^I n_i !\,. \end{aligned}$$

To avoid clumsy notation we defined $\;{\mathbb {B}}_{V}^{{\varvec{\alpha }}}\!({\varvec{n}})\!\;$ for all ${\varvec{n}}\in {\mathbb {Z}}^I$, but $\;{\mathbb {B}}_{V}^{{\varvec{\alpha }}}\!({\varvec{n}})\!\;= 0$ if ${\varvec{n}}\not \in {\mathcal {N}}$. We also see that $\;{\mathbb {B}}_{V}^{{\varvec{\alpha }}}\!({\varvec{n}})\!\;\approx V {\varvec{c}}^{\varvec{\alpha }}$ for ${\varvec{c}}= \frac{1}{V} {\varvec{n}}$, where the factor *V* indicates that the number of reactions is proportional to the volume of the container, if the densities are kept constant.

The CME associated with the RRE (2.2) is the Kolmogorov forward equation for the probability distributions ${\varvec{u}}=(u_{\varvec{n}})_{{\varvec{n}}\in {\mathcal {N}}} \in {\mathscr {P}}({\mathcal {N}})$, namely

3.2 $$\begin{aligned} \begin{aligned} {\dot{{\varvec{u}}}} = \sum _{r=1}^R \overline{{\mathcal {B}}}_V^r{\varvec{u}}\ \text {with } \big (\overline{{\mathcal {B}}}_V^r {\varvec{u}}\big )_{\varvec{n}}&= k^r_{\mathrm {fw}}\big ( \!\;{\mathbb {B}}_{V}^{{\varvec{\alpha }}^r}\!({\varvec{n}}{-}{\varvec{\beta }}^r)\!\; u_{{\varvec{n}}{+}{\varvec{\alpha }}^r{-}{\varvec{\beta }}^r} - \!\;{\mathbb {B}}_{V}^{{\varvec{\alpha }}^r}\!({\varvec{n}}{-}{\varvec{\alpha }}^r)\!\; u_{\varvec{n}}\big ) \\&\quad + k^r_{\mathrm {bw}}\big ( \!\;{\mathbb {B}}_{V}^{{\varvec{\beta }}^r}\!({\varvec{n}}{-}{\varvec{\alpha }}^r)\!\; u_{{\varvec{n}}{-}{\varvec{\alpha }}^r{+}{\varvec{\beta }}^r} - \!\;{\mathbb {B}}_{V}^{{\varvec{\beta }}^r}\!({\varvec{n}}{-}{\varvec{\beta }}^r)\!\; u_{\varvec{n}}\big ). \end{aligned} \end{aligned}$$

The *r*th forward reaction from ${\varvec{n}}{+}{\varvec{\alpha }}^r$ to ${\varvec{n}}{+}{\varvec{\beta }}^r$ can only happen (i.e., $\;{\mathbb {B}}_{V}^{{\varvec{\alpha }}^r}\!({\varvec{n}})\!\;>0$) if ${\varvec{n}}\ge \varvec{0}$. Hence any occurring $u_{\varvec{m}}$ with ${\varvec{m}}\not \in {\mathcal {N}}$ is multiplied by intensity 0, so in (3.2) we may set $u_{\varvec{m}}\equiv 0$ for all ${\varvec{m}}\not \in {\mathcal {N}}$. The operators $\overline{{\mathcal {B}}}_V^r$ are the adjoints of the Markov generators ${\mathcal {Q}}^r_V$ given by

3.3 $$\begin{aligned} ({\mathcal {Q}}_V^r {\varvec{\mu }})_{\varvec{n}}= k^r_{\mathrm {fw}}\!\;{\mathbb {B}}_{V}^{{\varvec{\alpha }}^r}\!({\varvec{n}}{-}{\varvec{\alpha }}^r)\!\; (\mu _{{\varvec{n}}-{\varvec{\alpha }}^r{+}{\varvec{\beta }}^r} - \mu _{\varvec{n}}) + k^r_{\mathrm {bw}}\!\;{\mathbb {B}}_{V}^{{\varvec{\beta }}^r}\!({\varvec{n}}{-}{\varvec{\beta }}^r)\!\; (\mu _{{\varvec{n}}+{\varvec{\alpha }}^r{-}{\varvec{\beta }}^r} - \mu _{\varvec{n}}) \end{aligned}$$

for $${\varvec{\mu }}=(\mu _{\varvec{n}})_{{\varvec{n}}\in {\mathcal {N}}}$$.

We emphasize that the RRE as well as the CME are uniquely specified if the reaction network (2.1), the reaction rates $k^r_{\mathrm {fw}}$ and $k^r_{\mathrm {bw}}$, and the volume $V>0$ are given. Hence, there are obviously close relations between both models, in particular for $V\gg 1$, see [4, 5, 23, 32, 61].

So far, we have not used the detailed-balance condition, i.e., we can even allow for $k^r_{\mathrm {bw}}= 0$ in the above considerations. In all cases, the Kolmogorov forward equation is an infinite-dimensional linear ODE as in Sect. 2.4. The following result shows that the detailed-balance condition is inherited from the RRE to the CME, and moreover a simple equilibrium ${\varvec{w}}^V$ can be given explicitly as a product distribution of individual Poisson distributions, namely $m\mapsto {\mathrm {e}}^{-V c_i^*} (V c_i^*)^m/m!$. This result can also be retrieved from [5] by combining Theorems 4.1 and 4.5 there, where it is shown that the weaker “complex-balance condition” is sufficient to guarantee that the Poisson distribution ${\varvec{w}}^V$ is an equilibrium for CME.

For completeness we give a short and independent proof of the fundamental result that for RRE with detailed balance the associated CME satisfies detailed balance again.

#### Theorem 3.1

(Detailed balance for CME) Let $\;{\mathbb {B}}_{V}^{{\varvec{\alpha }}}\!({\varvec{n}})\!\;$ be given in the form (3.1). Assume that (2.2) has the equilibrium ${\varvec{c}}_* \in ]0, \infty [^I$ satisfying the detailed-balance condition (2.6). Then the equilibrium ${\varvec{w}}^V:=(w^V_{\varvec{n}})_{{\varvec{n}}\in {\mathcal {N}}} \in {\mathscr {P}}({\mathcal {N}})$ given by

$$\begin{aligned} w^V_{\varvec{n}}=\frac{1}{Z^*_V} \, \frac{(V{\varvec{c}}_*)^{\varvec{n}}}{{\varvec{n}}!} \quad \text {with } Z^*_V:= \Pi _{i=1}^I {\mathrm {e}}^{V c_i^*} \end{aligned}$$

satisfies the detailed-balance condition for the CME (3.2), namely

$$\begin{aligned} \forall \, r=1,\ldots ,R\ \forall \, {\varvec{n}}\in {\mathcal {N}}: \ \ k^r_{\mathrm {fw}}\!\;{\mathbb {B}}_{V}^{{\varvec{\alpha }}^r}\!({\varvec{n}})\!\; w^V_{{\varvec{n}}+ {\varvec{\alpha }}^r} = k^r_{\mathrm {bw}}\!\;{\mathbb {B}}_{V}^{{\varvec{\beta }}^r}\!({\varvec{n}})\!\; w^V_{{\varvec{n}}+ {\varvec{\beta }}^r} = \kappa ^r_*V w^V_{\varvec{n}}=: {\widehat{\nu }}_V^{{\varvec{n}},r} . \end{aligned}$$

#### Proof

For each reaction we obtain the relation

$$\begin{aligned} k_{\mathrm {fw}}^r \!\;{\mathbb {B}}_{V}^{{\varvec{\alpha }}^r}\!({\varvec{n}})\!\; w^V_{{\varvec{n}}{+}{\varvec{\alpha }}^r} = k^r_{\mathrm {fw}}\frac{V({\varvec{n}}{+}{\varvec{\alpha }}^r)!}{V^{|{\varvec{\alpha }}^r|}{\varvec{n}}!} \frac{(V{\varvec{c}}_*)^{{\varvec{n}}{+}{\varvec{\alpha }}^r}}{Z^*_V ({\varvec{n}}{+}{\varvec{\alpha }}^r)!} = k^r_{\mathrm {fw}}V {\varvec{c}}_*^{{\varvec{\alpha }}^r} \frac{(V{\varvec{c}}_*)^{\varvec{n}}}{Z^*_V {\varvec{n}}!} = V \kappa ^r_* w_{\varvec{n}}^V. \end{aligned}$$

Analogously we obtain the same result for $k_{\mathrm {bw}}\!\;{\mathbb {B}}_{V}^{{\varvec{\beta }}^r}\!({\varvec{n}})\!\; w^V_{{\varvec{n}}+ {\varvec{\beta }}^r}$, where the detailed-balance condition (2.6) is used in the definition of $\kappa ^r_*$. $\square$

Using the detailed-balance coefficients ${\widehat{\nu }}_V^{{\varvec{n}},r}$ we can rewrite the operator $\overline{{\mathcal {B}}}^r_V$ from (3.2) in a symmetrically balanced form as

3.4 $$\begin{aligned} \overline{{\mathcal {B}}}^r_V{\varvec{u}}= \sum _{n\in {\mathcal {N}}} {\widehat{\nu }}^{{\varvec{n}},r}_V \Big (\frac{u_{{\varvec{n}}+{\varvec{\alpha }}^r}}{w^V_{{\varvec{n}}+{\varvec{\alpha }}^r}} - \frac{u_{{\varvec{n}}+{\varvec{\beta }}^r}}{w^V_{{\varvec{n}}+{\varvec{\beta }}^r}} \Big ) \big ({\varvec{e}}^{({\varvec{n}}+{\varvec{\beta }}^r)} {-} {\varvec{e}}^{({\varvec{n}}+{\varvec{\alpha }}^r)} \big ) , \end{aligned}$$

where ${\varvec{e}}^{({\varvec{m}})}$ is the unit vector, i.e., ${\varvec{e}}^{({\varvec{m}})}_{\varvec{n}}=\delta _{{\varvec{n}}{-}{\varvec{m}}}$.

It is important to realize that in general the steady state for the detailed-balance condition is highly non-unique, because of the discrete versions

$$\begin{aligned} {\mathcal {I}}(\overline{{\varvec{n}}}):= \big \{\, n\in {\mathcal {N}} \, \big | \, {\mathbb {Q}}{\varvec{n}}= {\mathbb {Q}}\overline{{\varvec{n}}} \,\big \} \ \subset \ {\mathcal {N}}\end{aligned}$$

of the invariant stoichiometric subspaces ${\varvec{I}}({\varvec{q}})= \{\, {\varvec{c}}\in {\varvec{C}} \, | \, {\mathbb {Q}}{\varvec{c}}={\varvec{q}} \,\} \subset {\varvec{C}}$. Indeed, choosing $\overline{n}$ arbitrary and defining $\overline{{\varvec{w}}}=(\overline{w}_{\varvec{n}}) \in {\mathscr {P}}({\mathcal {N}})$ via $\overline{w}_{\varvec{n}}= \frac{1}{Z} w_{\varvec{n}}^V$ for ${\varvec{n}}\in {\mathcal {I}}(\overline{{\varvec{n}}})$ and $\overline{w}_{\varvec{n}}=0$ elsewhere, we obtain another equilibrium for the CME (3.2). Defining convex combination we obtain a rich family of steady states.

The following counterexamples show that the above result, which is central to our work, cannot be expected for systems not satisfying the detailed-balance condition.

#### Example 3.2

(*Equation without detailed balance*) For $a, b \in {\mathbb {N}}$ we consider the RRE

3.5 $$\begin{aligned} {\dot{c}} = 2a - 4b\, c + 2\,(1{-}c^2), \end{aligned}$$

which consists of two individual reaction pairs, namely  and  with the individual steady states $c_{(1)}=a/(2b)$ and $c_{(2)}=1$. The joint steady state of (3.5) is $c_*=(1{+}a{+}b^2)^{1/2}-b$, and we have detailed balance if and only if $a=2b$.

Building the CME according to (3.2) based on the two reaction pairs we obtain

$$\begin{aligned} {\dot{u}}_n= & {} 2aVu_{n-1}-(2aV{+}4bn)u_n + 4b(n{+}1)u_{n+1} + V u_{n-2}\\&-\big (V+\tfrac{n(n{-}1)_+}{V} \big ) u_n + \tfrac{(n{+}2)(n{+}1)}{V} u_{n+2} . \end{aligned}$$

For the case $a=2$ and $b=1$, where the detailed-balance condition holds with $c_*=1=c_{(1)}=c_{(2)}$, we obtain

$$\begin{aligned} {\dot{u}}_n =V u_{n-2} + 4Vu_{n-1} -\big (5V+4n+\tfrac{n(n{-}1)_+}{V} \big ) u_n + 4(n{+}1)u_{n+1} +\tfrac{(n{+}2)(n{+}1)}{V} u_{n+2} , \end{aligned}$$

and it is easy to check that $\widetilde{\varvec{w}}^V=({\mathrm {e}}^{-V} V^n/n!)_{n\in {\mathbb {N}}_0}$ is a steady state.

However, for $a=7$ and $b=1$ the detailed-balance condition fails with $c_{(1)}=7/2> c_*=2> c_{(2)}=1$. The CME reads

$$\begin{aligned} {\dot{u}}_n = V u_{n-2}+ 14Vu_{n-1} -\big ( 15V{+}4n+\tfrac{n(n{-}1)_+}{V} \big ) u_n + 4(n{+}1)u_{n+1}+ \tfrac{(n{+}2)(n{+}1)}{V} u_{n+2} . \end{aligned}$$

An explicit calculation shows that the Poisson distribution $\widetilde{\varvec{w}}^V$ based on $c_*=2$, i.e., $\widetilde{w}^V_n={\mathrm {e}}^{-2V} (2V)^n/n!$, is not a steady state. Indeed, inserting $\widetilde{\varvec{w}}^V$ into the right-hand side of the last equation we find (for $n\ge 1$)

$$\begin{aligned} {{\dot{u}}_n|_{{\varvec{u}}= \widetilde{\varvec{w}}^V} = \frac{{\mathrm {e}}^{-2V}(2V)^{n-2}}{n!} \Big ( {-}12 V^3 + 12nV^2 -3n(n{-}1) V\Big ) \ne 0 \text { for general } n \in {\mathbb {N}}. } \end{aligned}$$

#### Example 3.3

(*Microscopic versus macroscopic detailed balance*) We may also consider a RRE that looks macroscopically as being in detailed balance, but is generated by a microscopic model that is not in detailed balance. The two reactions $\emptyset \overset{2}{\rightharpoonup } X$ and $2X \overset{1}{\rightharpoonup } \emptyset$ produce the RRE ${\dot{c}} = 2 (1 {-}c^2)$ that has the equilibrium $c_*=1$. However the CME reads

$$\begin{aligned} {\dot{u}}_n = 2V u_{n-1} - \big ( 2V + \frac{n(n{-}1)}{ V}\big ) u_n + \frac{(n{+}2)(n{+}1)}{V} u_{n+2} . \end{aligned}$$

Again, the Poisson distribution $\widetilde{\varvec{w}}^V$ with $w^V_n={\mathrm {e}}^{-V}V^n/n!$ is *not* the equilibrium:

$$\begin{aligned} {\dot{u}}_n|_{{\varvec{u}}=\widetilde{\varvec{w}}^V} = \frac{{\mathrm {e}}^{-V}V^{n-1}}{n!}\, \Big ( 2Vn - V^2 {-} n(n{-}1) \Big ) \ \ne \ 0. \end{aligned}$$

Note that the reversible reaction pair  yields the same RRE, and its associated CME satisfies the detailed-balance condition.

### Existence and Uniqueness of Solutions of CME

In this part we establish well-posedness for the CME. We do this by combining classical results from the theory of Markov chains with abstract semigroup theory.

For fixed ${\varvec{n}}_0 \in {\mathcal {N}}$ we construct a special Green’s function $p_t({\varvec{n}}_0,\cdot )$. General Markov chain theory (e.g., [36, Ch. 2]) implies that there exist a unique minimal solution $[0,\infty [ \times {\mathcal {N}}\ni (t, {\varvec{n}}) \mapsto p_t({\varvec{n}}_0, {\varvec{n}})$ to the backward equation

$$\begin{aligned} {\dot{p}}_t({\varvec{n}}_0, {\varvec{n}})&= \sum _{r = 1}^R\Big ( k^r_{\mathrm {fw}}\!\;{\mathbb {B}}_{V}^{{\varvec{\alpha }}^r}\!({\varvec{n}}_0{-}{\varvec{\alpha }}^r)\!\; \big ( p_t({\varvec{n}}_0 {-} {\varvec{\alpha }}^r {+} {\varvec{\beta }}^r , {\varvec{n}}) - p_t({\varvec{n}}_0, {\varvec{n}})\big ) \\&\quad + k^r_{\mathrm {bw}}\!\;{\mathbb {B}}_{V}^{{\varvec{\beta }}^r}\!({\varvec{n}}_0{-}{\varvec{\beta }}^r)\!\; \big ( p_t({\varvec{n}}_0 {+} {\varvec{\alpha }}^r {-} {\varvec{\beta }}^r, {\varvec{n}}) - p_t({\varvec{n}}_0, {\varvec{n}}) \big ) \Big ) \end{aligned}$$

associated with the CME with initial condition $p_0({\varvec{n}}_0, {\varvec{n}}) = \delta _{{\varvec{n}}_0}({\varvec{n}})$. This minimal solution is non-negative and satisfies $p_t({\varvec{n}}_0,{\varvec{n}})\ge 0$ and $\sum _{{\varvec{n}}\in {\mathcal {N}}} p_t({\varvec{n}}_0, {\varvec{n}}) \le 1$, but for general CTMC it can happen that the latter inequality is strict, which means that the corresponding Markov chain explodes in finite time. We will show that explosion does not happen for CME with detailed balance.

For the functional analytic existence and uniqueness result we use the sequence spaces

$$\begin{aligned} \ell ^p({\mathcal {N}}):= \big \{\, {\varvec{u}}=(u_{\varvec{n}})_{{\varvec{n}}\in {\mathcal {N}}} \, \big | \, \sum \nolimits _{{\varvec{n}}\in {\mathcal {N}}}|u_{\varvec{n}}|^p <\infty \,\big \} \end{aligned}$$

as well as the weighted spaces

$$\begin{aligned} {\mathrm {L}}^p({\mathcal {N}},{\varvec{w}}^V):= \big \{\, {\varvec{v}}=(v_{\varvec{n}})_{{\varvec{n}}\in {\mathcal {N}}} \, \big | \, \sum \nolimits _{{\varvec{n}}\in {\mathcal {N}}}\big |\frac{v_{\varvec{n}}}{w^V_{\varvec{n}}} \big |^p <\infty \,\big \} \end{aligned}$$

with the corresponding norms and the usual modification for $p=\infty$. Now, we consider the transition semigroup $({\mathcal {P}}_t)_{t \ge 0}$ defined by

$$\begin{aligned} ({\mathcal {P}}_t {\varvec{v}})_{\varvec{n}}:= \sum _{{\varvec{m}}\in {\mathcal {N}}} p_t({\varvec{n}},{\varvec{m}})v_{\varvec{m}},\quad {\varvec{v}}=(v_{\varvec{m}}) \in \ell ^\infty ({\mathcal {N}}), \end{aligned}$$

which we shall study by induction over the number *R* of reactions using the Trotter-Kato formula, where the detailed-balance condition guarantees that each subsystem is a contraction semigroup on ${\mathrm {L}}^2({\mathcal {N}},{\varvec{w}}^V)$.

#### Theorem 3.4

Assume that the detailed balance condition (2.6) holds. Then, the semigroup $({\mathcal {P}}_t)_{t \ge 0}$ extends to a ${\mathrm {C}}_0$-semigroup of contractions on ${\mathrm {L}}^p({\mathcal {N}}, {\varvec{w}}^V)$ for all $1 \le p < \infty$. Moreover, the semigroup is selfadjoint on ${\mathrm {L}}^2({\mathcal {N}}, {\varvec{w}}^V)$ and Markovian, i.e., ${\mathcal {P}}_t \mathbf{1} = \mathbf{1}$ for all $t \ge 0$.

A related existence result for the Markov semigroup of the CME was established in [22], which however does not apply to the case of reversible RRE, because of the restrictions on the growth of the transition rates.

#### Proof

All of the above statements follow from the general theory of continuous time Markov chains, except for the Markovianity. To show the latter, we first consider the case of a single reaction, thus $R = 1$. Each of the irreducible components of the state space ${\mathcal {N}}$ is then one-dimensional (see also [38]), and the Markov chain is a birth-death chain on a countable (possibly finite) set.

If there exist two components of ${\varvec{\alpha }}- {\varvec{\beta }}$ with opposite sign, then each of the irreducible components of the state space ${\mathcal {N}}$ is finite. Therefore it is clear that the Markov chain does not explode in finite time. Suppose now that all components of ${\varvec{\alpha }}- {\varvec{\beta }}$ have equal sign, say $\alpha _i - \beta _i \ge 0$ for all $i =1, \ldots , I$, and at least one component is strictly positive. Then each of the infinite irreducible components of ${\mathcal {N}}$ is of the form

$$\begin{aligned} \{\, {\varvec{n}}^{(k)} := {\varvec{n}}^{(0)} + k ({\varvec{\alpha }}- {\varvec{\beta }}) \, | \, k \in {\mathbb {N}}_0 \,\} \end{aligned}$$

for some ${\varvec{n}}^{(0)} \in {\mathcal {N}}$, and the restricted Markov process is a birth-death process with birth rate $\mathsf {b}_k$ and death rate $\mathsf {d}_k$ given by

$$\begin{aligned} \mathsf {b}_k&:= k_{\mathrm {bw}}\!\;{\mathbb {B}}_{V}^{{\varvec{\beta }}}\!({\varvec{n}}^{(k)}{-}{\varvec{\beta }})\!\; \ \text { from } {\varvec{n}}^{(k)}\text { to }{\varvec{n}}^{(k+1)} \quad \text {and} \quad \\ \mathsf {d}_k&:= k_{\mathrm {fw}}\!\;{\mathbb {B}}_{V}^{{\varvec{\alpha }}}\!({\varvec{n}}^{(k)}{-}{\varvec{\alpha }})\!\; \ \text { from }{\varvec{n}}^{(k)}\text { to }{\varvec{n}}^{(k-1)}. \end{aligned}$$

Reuter’s criterion [53, Thm. 11] gives a characterization of non-explosion for birth-death chains; it asserts that the chain is non-explosive if and only if

$$\begin{aligned} \sum _{k \ge j \ge 0} \mathsf {r}_{j,k} =\infty , \quad \text {where }\mathsf {r}_{j,k}:= \frac{\mathsf {d}_k \cdot \ldots \cdot \mathsf {d}_{j+1}}{\mathsf {b}_{k} \cdot \ldots \cdot \mathsf {b}_j} . \end{aligned}$$

In our setting we have $\frac{\mathsf {d}_{k+1}}{\mathsf {b}_k} = (V {\varvec{c}}^*)^{{\varvec{\beta }}- {\varvec{\alpha }}} \frac{{\varvec{n}}^{(k+1)}!}{{\varvec{n}}^{(k)}!}$, so that $\mathsf {r}_{0,k} = \frac{1}{\mathsf {b}_k}(V {\varvec{c}}^*)^{k({\varvec{\beta }}- {\varvec{\alpha }})} \frac{{\varvec{n}}^{(k)}!}{{\varvec{n}}^{(0)}!}$, and therefore

$$\begin{aligned} \sum _{k \ge j \ge 0} \mathsf {r}_{j,k} \ge \sum _{k \ge 0} \mathsf {r}_{0,k} \ge \frac{V^{|{\varvec{\beta }}|-1}}{k_{\mathrm {bw}}{\varvec{n}}^{(0)}!} \sum _{k \ge 0} \frac{({\varvec{n}}^{(k)} {-}{\varvec{\beta }})!}{(V {\varvec{c}}^*)^{k({\varvec{\alpha }}- {\varvec{\beta }})}}. \end{aligned}$$

Since the summands tend to $\infty$ as $k \rightarrow \infty$, we infer that the latter sum is infinite; hence the Markov chain is non-explosive, or equivalently ${\mathcal {P}}_t \mathbf{1} = \mathbf{1}$ (see [36, Thm. 2.33]).

The case of multiple reactions follows by induction on the number of reactions *R*. Indeed, for ${\mathcal {R}}\subseteq \{1, \ldots , R \}$, let $({\mathcal {P}}^{{\mathcal {R}}}_t)_{t \ge 0}$ denote the semigroup corresponding to the reactions $r \in {\mathcal {R}}$. Then the Trotter product formula for contraction semigroups on ${\mathrm {L}}^2({\mathcal {N}},{\varvec{w}}^V)$ (see e.g., [9]) asserts that

$$\begin{aligned} {\mathcal {P}}^{\{1, \ldots , R+1\}}_t = \lim _{n \rightarrow \infty } \Big ( {\mathcal {P}}^{\{1, \ldots , R\}}_{t/n} {\mathcal {P}}^{\{R+1\}}_{t/n} \Big )^n \end{aligned}$$

strongly in ${\mathrm {L}}^2({\mathcal {N}},{\varvec{w}}^V)$. Note that we can apply this formula, since the detailed-balance conditions hold for all reactions simultaneously, hence all of the semigroups are contractive on the same space ${\mathrm {L}}^2({\mathcal {N}},{\varvec{w}}^V)$. We also observe that the class of finitely supported functions is a core for each of the generators. The Markovianity of ${\mathcal {P}}^{\{1, \ldots , R+1\}}$ thus follows from the Trotter formula and the Markovianity of ${\mathcal {P}}^{\{1, \ldots , R\}}$ and ${\mathcal {P}}^{\{R+1\}}$. $\square$

#### Remark 3.5

The mere existence of a probability distribution satisfying the detailed-balance equations is not sufficient to guarantee non-explosion of a continuous time Markov chain. It might happen that the chain jumps infinitely often in a finite time interval, see [50, Sec. 3.5] for an example. The previous result shows that this phenomenon does not occur in CME satisfying the detailed-balance condition.

It remains to transfer the results from ${\mathrm {L}}^1({\mathcal {N}},{\varvec{w}}^V)$ to $\ell ^1({\mathcal {N}})$. Denoting by ${\mathcal {Q}}$ the generator of the $C_0$-semigroup $({\mathcal {P}}_t)_{t\ge 0}$ on ${\mathrm {L}}^1({\mathcal {N}},{\varvec{w}}^V)$, we define the operator ${\mathcal {B}}: \text {Dom}({\mathcal {B}}) \subseteq \ell ^1({\mathcal {N}}) \rightarrow \ell ^1({\mathcal {N}})$ by

$$\begin{aligned} {\mathcal {B}}{\varvec{u}}= {\varvec{w}}^V {\mathcal {Q}}({\varvec{u}}/ {\varvec{w}}^V), \quad \text {Dom}({\mathcal {B}}) = \{\, {\varvec{u}}\in \ell ^1({\mathcal {N}}) \, | \, {\varvec{u}}/ {\varvec{w}}^V \in \text {Dom}({\mathcal {Q}}) \,\} . \end{aligned}$$

This definition of ${\mathcal {B}}$ is consistent with the explicit formula for ${\mathcal {B}}$ given above. Since ${\mathcal {Q}}$ generates a $C_0$-semigroup of contractions on ${\mathrm {L}}^1({\mathcal {N}},{\varvec{w}}^V)$, it follows that ${\mathcal {B}}$ generates a $C_0$-semigroup $({\mathcal {P}}_t)_{t \ge 0}$ of contractions on $\ell ^1({\mathcal {N}})$. Furthermore, since ${\mathcal {P}}_t$ preserves positivity and ${\mathcal {P}}_t \mathbf{1} = \mathbf{1}$, it follows that ${\mathscr {P}}({\mathcal {N}})$ is invariant under the semigroup generated by ${\mathcal {B}}$.

As an immediate consequence we obtain global well-posedness for the CME in ${\mathscr {P}}({\mathcal {N}})$.

#### Theorem 3.6

(Global well-posedness of the CME) Let the detailed-balance condition (2.6) hold. Then, for all ${\varvec{u}}_0 \in {\mathscr {P}}({\mathcal {N}})$ there exists a unique mild solution ${\varvec{u}}: [0,\infty ) \rightarrow {\mathscr {P}}({\mathcal {N}})$ to the CME (3.2) satisfying ${\varvec{u}}(0) = {\varvec{u}}_0$.

### Gradient Structures for CME

Since the CME is the forward equation associated with a reversible CTMC, we can formulate it as a gradient flow in view of Proposition 2.3. Indeed, for a strictly convex function $\phi :[0,\infty [ \rightarrow {\mathbb {R}}$ that is smooth on $]0,\infty [$, let us write

3.6 $$\begin{aligned}&{\mathcal {E}}_V^\phi ({\varvec{u}}):= \sum _{{\varvec{n}}\in {\mathcal {N}}} w^V_{\varvec{n}}\,\phi \big ( \frac{u_{\varvec{n}}}{w^V_{\varvec{n}}}\big ), \nonumber \\&{\mathcal {K}}_V^\phi ({\varvec{u}}):= \sum _{{\varvec{n}}\in {\mathcal {N}}} \sum _{r=1}^R \widehat{\nu }_V^{{\varvec{n}},r} \,\Phi \Big ( \frac{u_{{\varvec{n}}+{\varvec{\alpha }}^r}}{w^V_{{\varvec{n}}+{\varvec{\alpha }}^r}}\, , \, \frac{u_{{\varvec{n}}+{\varvec{\beta }}^r}}{w^V_{{\varvec{n}}+{\varvec{\beta }}^r}} \Big ) \, \left( {\varvec{e}}^{({\varvec{n}}+{\varvec{\alpha }}^r)} {-} {\varvec{e}}^{({\varvec{n}}+ {\varvec{\beta }}^r)}\right) {\otimes }\left( {\varvec{e}}^{({\varvec{n}}+{\varvec{\alpha }}^r)} {-} {\varvec{e}}^{({\varvec{n}}+ {\varvec{\beta }}^r)} \right) , \end{aligned}$$

where $\Phi$ is defined after (2.16), $\widehat{\nu }^{{\varvec{n}},r}_V$ is given in Theorem 3.1, and ${\varvec{e}}^{({\varvec{m}})}$ denotes the ${\varvec{m}}$-th unit vector in $\ell ^1({\mathcal {N}})$. The following result is then a special case of Proposition 2.3.

#### Proposition 3.7

(Quadratic gradient structures for CME) If the RRE (2.2) satisfies the detailed-balance condition (2.6) for a positive steady state ${\varvec{c}}_*=(c^*_i)_{i=1,\ldots ,I}$, then the associated CME has the gradient structure $({\mathscr {P}}({\mathcal {N}}), {\mathcal {E}}_V^\phi , {\mathcal {K}}_V^\phi )$ defined in (3.6), namely

$$\begin{aligned} {\dot{{\varvec{u}}}} = {\mathcal {B}}_V{\varvec{u}}=- {\mathcal {K}}_V^\phi ({\varvec{u}}) {\mathrm {D}}{\mathcal {E}}_V^\phi ({\varvec{u}}). \end{aligned}$$

In the following we will mainly be concerned with the case that ${\mathcal {E}}_V^\phi$ is the logarithmic entropy, where $\phi$ is the Boltzmann function $\lambda _{\mathrm {B}}(z) = z\log z -z +1$. In that case we obtain

3.7 $$\begin{aligned}&{\mathcal {E}}_V({\varvec{u}}) := \frac{1}{V} \sum _{{\varvec{n}}\in {\mathcal {N}}} w_{\varvec{n}}^V \lambda _{\mathrm {B}}\big ( \frac{u_{\varvec{n}}}{w_{\varvec{n}}^V} \big ) = \frac{1}{V} \sum _{{\varvec{n}}\in {\mathcal {N}}}\big ( u_{\varvec{n}}\log u_{\varvec{n}}- u_{\varvec{n}}\log w^V_{\varvec{n}}\big ) ,\nonumber \\&{\mathcal {K}}_V({\varvec{u}}):= V \sum _{r=1}^R \sum _{{\varvec{n}}\in {\mathcal {N}}} \widehat{\nu }_V^{{\varvec{n}},r} \, \Lambda \Big ( \frac{u_{{\varvec{n}}+{\varvec{\alpha }}^r}}{w^V_{{\varvec{n}}+{\varvec{\alpha }}^r}} , \frac{u_{{\varvec{n}}+{\varvec{\beta }}^r}}{w^V_{{\varvec{n}}+{\varvec{\beta }}^r}} \Big ) \, ({\varvec{e}}^{({\varvec{n}}+{\varvec{\alpha }}^r)} {-} {\varvec{e}}^{({\varvec{n}}+ {\varvec{\beta }}^r)} ) {\otimes }({\varvec{e}}^{({\varvec{n}}+{\varvec{\alpha }}^r)} {-} {\varvec{e}}^{({\varvec{n}}+ {\varvec{\beta }}^r)} ), \end{aligned}$$

where the logarithmic mean $\Lambda (a,b)$ is defined in (2.10). The above definitions do not only restrict to the entropy function $\phi =\lambda _{\mathrm {B}}$, but also introduce a normalization with respect to the volume *V*. Hence, ${\mathcal {E}}_V$ can be seen as an entropy per unit volume. The corresponding scaling of ${\mathcal {K}}_V$ was chosen such that the evolution equation ${\dot{{\varvec{u}}}} = - {\mathcal {K}}_V({\varvec{u}}){\mathrm {D}}{\mathcal {E}}_V({\varvec{u}})$ is the same as ${\dot{{\varvec{u}}}} =- {\mathcal {K}}_V^\phi ({\varvec{u}}) {\mathrm {D}}{\mathcal {E}}_V^\phi ({\varvec{u}})$.

For later purposes we also provide the cosh-type gradient structure for CME, whose relevance and usefulness is discussed in [21, 43, 44, 46]. Recall the definition of ${\mathsf {C}}^*$ in (2.18) and note the special scaling via the volume *V* in (3.8) below, which is needed because ${\varvec{\Psi }}^*_{\cosh ,V}({\varvec{u}},\cdot )$ is not scaling invariant.

#### Proposition 3.8

(Cosh-type gradient structure for CME) If the RRE (2.2) satisfies the detailed-balance condition (2.6) for a positive steady state ${\varvec{c}}_*=(c^*_i)_{i=1,\ldots ,I}$, then the associated CME has the gradient structure $({\mathscr {P}}({\mathcal {N}}), {\mathcal {E}}_V, {\varvec{\Psi }}^*_{\cosh ,V})$ with ${\mathcal {E}}_V$ from (3.7) and

3.8 $$\begin{aligned} {\varvec{\Psi }}^*_{\cosh ,V}({\varvec{u}},{\varvec{\mu }}):= \frac{1}{V} \sum _{r=1}^R \sum _{n\in {\mathcal {N}}} \widehat{\nu }_V^{{\varvec{n}},r} \; \Big ( \frac{u_{{\varvec{n}}+{\varvec{\alpha }}^r}}{w^V_{{\varvec{n}}+{\varvec{\alpha }}^r}} \, \frac{u_{{\varvec{n}}+{\varvec{\beta }}^r}}{w^V_{{\varvec{n}}+{\varvec{\beta }}^r}} \Big )^{1/2}\, {\mathsf {C}}^*\Big ( V (\mu _{{\varvec{n}}+ {\varvec{\beta }}^r}{-} \mu _{{\varvec{n}}+{\varvec{\alpha }}^r})\Big ) . \end{aligned}$$

#### Proof

The desired formula $\sum _{r=1}^R \overline{{\mathcal {B}}}^r_V {\varvec{u}}= {\mathrm {D}}_\mu \Psi _{\cosh ,V}^*({\varvec{u}},-{\mathrm {D}}{\mathcal {E}}_V({\varvec{u}})\big )$ follows easily by recalling $\overline{{\mathcal {B}}}_V^r$ from (3.4) and by using $\sqrt{ab}\,({\mathsf {C}}^*)'\big (\log a - \log b\big )=a{-}b$ and ${\mathrm {D}}{\mathcal {E}}_V({\varvec{u}})=\frac{1}{V} \big ( \log (u_{\varvec{n}}/ w^V_{\varvec{n}})\big )_{{\varvec{n}}\in {\mathcal {N}}}$. $\square$

## Liouville and Fokker–Planck Equations

For general evolutionary equations one can define a measure-valued flow in the phase space that is given by transporting the measures according to the semiflow of the original equation. The evolution equation describing this measure-valued flow is the Liouville equation. For our RRE ${\dot{{\varvec{c}}}} = -{\varvec{R}}({\varvec{c}})$ in ${\varvec{C}}:={[0,\infty [}^I$ we assume that we have a global semiflow ${\varvec{c}}(t)=\Phi _t({\varvec{c}}(0))$ and consider probability measures $\varrho (t,\cdot ) \in {\mathscr {P}}({\varvec{C}})$ that are obtained by transporting $\varrho _0$ with $\Phi _t$, namely

$$\begin{aligned} \varrho (t,\cdot )=\Phi _t^\#\varrho _0, \quad \text {i.e., }\, \forall \,\psi \in {\mathrm {C}}_b({\varvec{C}}): \int _{\varvec{C}}\psi ({\varvec{c}}) \varrho (t,{\mathrm {d}}{\varvec{c}}) = \int _{\varvec{C}}\psi (\Phi _t({\varvec{c}})) \varrho _0({\mathrm {d}}{\varvec{c}}). \end{aligned}$$

In particular, if $\varrho _0 = \sum _{k=1}^m a_k \delta _{{\varvec{c}}^k_0}$, then $\varrho (t,\cdot )= \sum _{k=1}^m a_k \delta _{\Phi _t({\varvec{c}}^k_0)}(\cdot )$.

It is now easy to see that $t\mapsto \varrho _t \in {\mathscr {P}}({\varvec{C}})$ satisfies the Liouville equation

4.1 $$\begin{aligned} \partial _t\varrho (t,{\varvec{c}}) = {\mathrm {div}}\big (\varrho (t,{\varvec{c}}) {\varvec{R}}({\varvec{c}})\big ), \end{aligned}$$

in the sense of distributions. We will regard (4.1) as an evolution equation in the space ${\mathscr {P}}({\varvec{C}})$. We will not always notationally distinguish between an absolutely continuous probability measure and its density, but if we want to distinguish them we will write $\varrho ({\mathrm {d}}{\varvec{c}}) = \rho ({\varvec{c}}) \;\!\mathrm {d}{\varvec{c}}$ with $\rho \in {\mathrm {L}}^1({\varvec{C}})$.

The goal of this section is to give a rigorous connection between the CME for $V\rightarrow \infty$ and the Liouville equation in terms of the associated gradient structures.

### The Liouville Equation as a Gradient System

We show that the gradient structure ${\dot{{\varvec{c}}}} = -{\varvec{R}}({\varvec{c}})=- {\mathbb {K}}({\varvec{c}}) {\mathrm {D}}E({\varvec{c}})$ for the RRE, which was discussed in Sect. 2.3, induces a natural gradient structure for the Liouville equation. Consider the “Otto-Wasserstein-type” Onsager operator ${\varvec{K}}(\varrho )$ that acts on functions $\xi : {\varvec{C}}\rightarrow {\mathbb {R}}$ via

$$\begin{aligned} {\varvec{K}}(\varrho )\xi = -{\mathrm {div}}\big ( \varrho \, {\mathbb {K}}\, \nabla \xi \big ), \end{aligned}$$

where ${\mathrm {div}}$ and $\nabla$ are taken with respect to ${\varvec{c}}\in {\mathbb {R}}^I$. We also consider the affine potential energy functional ${\varvec{E}}: {\mathscr {P}}({\varvec{C}}) \rightarrow [0,+\infty ]$ defined by

4.2 $$\begin{aligned} {\varvec{E}}(\varrho ) = \int _{\varvec{C}}E({\varvec{c}}) \;\!\mathrm {d}\varrho ({\varvec{c}}). \end{aligned}$$

In the next result we identify the formal gradient structure for the Liouville equation.

#### Proposition 4.1

(Gradient structure for the Liouville equation) If the RRE (2.2) satisfies the detailed-balance condition (2.6) for a positive steady state ${\varvec{c}}_*=(c^*_i)_{i=1,\ldots ,I}$, then the associated Liouville equation has the gradient structure $({\mathscr {P}}({\varvec{C}}) , {\varvec{E}}, {\varvec{K}})$, namely

4.3 $$\begin{aligned} {\dot{\varrho }} = - {\varvec{K}}(\varrho ) {\mathrm {D}}{\varvec{E}}(\varrho )= {\mathrm {div}}\big ( \varrho {\mathbb {K}}\nabla E\big ) = {\mathrm {div}}(\varrho {\varvec{R}}) . \end{aligned}$$

#### Proof

Let $\varrho \in {\mathscr {P}}({\varvec{C}})$ and let $\sigma \in {\mathscr {M}}({\varvec{C}})$ be a signed measure of finite total variation such that $\sigma ({\varvec{C}}) = 0$ and $\varrho + h \sigma \in {\mathscr {P}}({\varvec{C}})$ for |*h*| sufficiently small. Then we have

$$\begin{aligned} \frac{{\varvec{E}}(\varrho + h \sigma ) - {\varvec{E}}(\varrho )}{h} = \int _{\varvec{C}}E({\varvec{c}}) \;\!\mathrm {d}\sigma ({\varvec{c}}), \end{aligned}$$

hence ${\mathrm {D}}{\varvec{E}}(\varrho ) = E$ for all $\varrho$. Therefore, $- {\varvec{K}}(\varrho ) {\mathrm {D}}{\varvec{E}}(\varrho ) = {\mathrm {div}}\big ( \varrho \, {\mathbb {K}}\, \nabla E\big ) = {\mathrm {div}}( \varrho {\varvec{R}}).$ The gradient flow equation ${\dot{\varrho }} = - {\varvec{K}}(\varrho ) {\mathrm {D}}{\varvec{E}}(\varrho )$ is thus given by the Liouville equation (4.1). $\square$

### Passing to the Limit from CME to Liouville

In this section we shall demonstrate that the gradient flow structure for the CME converges in a suitable sense to the gradient structure for the Liouville equation if $V \rightarrow \infty$.

More precisely, we will show that after a suitable *V*-dependent embedding of ${\mathscr {P}}({\mathcal {N}})$ into ${\mathscr {P}}({\varvec{C}})$ the proper scalings of the functionals ${\mathcal {E}}_V$ and $\Psi _V^*:({\varvec{u}},{\varvec{\mu }})\mapsto \frac{1}{2}{\varvec{\mu }}\cdot {\mathcal {K}}_V({\varvec{u}}){\varvec{\mu }}$ converge in the sense of $\Gamma$-convergence to the corresponding structures for the Liouville equation given by the gradient system $({\mathscr {P}}({\varvec{C}}),{\varvec{E}},{\varvec{K}})$, see Sect. 4.3 to 4.5. Following the approach in [41, 54, 58], and in particular [35], we are then able to establish the convergence for $V\rightarrow \infty$ of solutions ${\varvec{u}}^V:{[0,\infty [}\rightarrow {\mathscr {P}}({\mathcal {N}})$ of the CME ${\dot{{\varvec{u}}}}^V = - {\mathcal {K}}_V({\varvec{u}}^V) {\mathrm {D}}{\mathcal {E}}_V({\varvec{u}}^V)$ to the solution $\varrho :{[0,\infty [}\rightarrow {\mathscr {P}}({\varvec{C}})$ of the Liouville equation ${\dot{\varrho }} = - {\varvec{K}}(\varrho ) {\mathrm {D}}{\varvec{E}}(\varrho )$, thereby recovering Kurtz’ result (1.3), see Sect. 4.6.

The main tool for proving this evolutionary $\Gamma$-convergence for gradient systems is the so-called *energy–dissipation principle*, cf. [41, Sect. 3.3], which states that ${\varvec{u}}^V$ solves the CME if and only if for all $T>0$ the following energy-dissipation estimate holds:

4.4 $$\begin{aligned} {\mathcal {E}}_V({\varvec{u}}^V(T)) + \int _0^T \Big (\Psi _V({\varvec{u}}^V,{\dot{{\varvec{u}}}}^V) + \Psi _V^*\big ({\varvec{u}}^V, {-}{\mathrm {D}}{\mathcal {E}}_V({\varvec{u}}^V)\big )\Big ) \;\!\mathrm {d}t \le {\mathcal {E}}_V({\varvec{u}}^V(0)), \end{aligned}$$

where we use the quadratic dissipation potential $\Psi _V$ and its Legendre dual $\Psi ^*_V$ defined via $\Psi _V^* ({\varvec{u}},{\varvec{\mu }}) := \frac{1}{2}\langle {\varvec{\mu }},{\mathcal {K}}_V({\varvec{u}}){\varvec{\mu }}\rangle$ with ${\mathcal {K}}_V$ from (3.7), namely

4.5a $$\begin{aligned} \Psi _V^*({\varvec{u}},{\varvec{\mu }})&= \frac{V}{2} \sum _{{\varvec{n}}\in {\mathcal {N}}}\sum _{r=1}^R \widehat{\nu }^{{\varvec{n}},r}_V \Lambda \big ( \frac{u_{{\varvec{n}}+{\varvec{\alpha }}^r}}{w^V_{{\varvec{n}}+{\varvec{\alpha }}^r}} , \frac{u_{{\varvec{n}}+{\varvec{\beta }}^r}}{w^V_{{\varvec{n}}+{\varvec{\beta }}^r}} \big ) \big ( \mu _{{\varvec{n}}+{\varvec{\alpha }}^r} {-} \mu _{{\varvec{n}}+{\varvec{\beta }}^r}\big )^2 \end{aligned}$$

4.5b $$\begin{aligned}&= \frac{V}{2} \sum _{{\varvec{n}}\in {\mathcal {N}}}\sum _{r=1}^R \Lambda \big ( k^r_{\mathrm {fw}}\!\;{\mathbb {B}}_{V}^{{\varvec{\alpha }}^r}\!({\varvec{n}})\!\; u_{{\varvec{n}}+{\varvec{\alpha }}^r} , k^r_{\mathrm {bw}}\!\;{\mathbb {B}}_{V}^{{\varvec{\beta }}^r}\!({\varvec{n}})\!\; u_{{\varvec{n}}+{\varvec{\beta }}^r}\big ) \big ( \mu _{{\varvec{n}}+{\varvec{\alpha }}^r} {-} \mu _{{\varvec{n}}+{\varvec{\beta }}^r}\big )^2, \end{aligned}$$

where the second form uses Theorem 3.1 and is especially useful to perform the limit $V \rightarrow \infty$, see the proof of Proposition .

We refer to [8, 41] for this equivalence and general methods for proving such results on evolutionary $\Gamma$-convergence. In [11] a similar approach was used to establish the convergence of CTMC to a Fokker–Planck equation. However, there the convergence of a parabolic equation is established, where upper and lower bounds of the density can be used. Here, the importance is that our limit measures $\varrho (t)$ may not have densities; indeed, because we want to recover the Kurtz result (1.3) we are interested in the “deterministic case” $\varrho (t)=\delta _{{\varvec{c}}(t)}$. So our analysis has to be more careful in dealing with general limit measures. For this, we use the dualization approach introduced in [35] where $t\mapsto \Psi _V({\varvec{u}}^V,{\dot{{\varvec{u}}}}^V)$ is estimated from below by $\langle {\dot{{\varvec{u}}}}^V, {\varvec{\mu }}^V \rangle - \Psi ^*_V({\varvec{u}}^V,{\varvec{\mu }}^V)$ for suitably chosen recovery functions $t\mapsto {\varvec{\mu }}^V(t)$.

In order to compare probability measures on different spaces ${\mathcal {N}}$ and ${\varvec{C}}$, we consider a suitable embedding $\iota _V : {\mathscr {P}}({\mathcal {N}}) \rightarrow {\mathscr {P}}({\varvec{C}})$. Here $\iota _V({\varvec{u}})$ is simply obtained by assigning the mass of ${\varvec{u}}$ at ${\varvec{n}}\in {\mathcal {N}}$ uniformly to the cube

$$\begin{aligned} A_{\varvec{n}}^V := \big [\tfrac{n_1}{V}, \tfrac{n_1 + 1}{V} \big [ \times \cdots \times \big [\tfrac{n_I}{V}, \tfrac{n_I + 1}{V}\big [ \subseteq {\varvec{C}}. \end{aligned}$$

More explicitly, $\iota _V({\varvec{u}})$ is given by

4.6 $$\begin{aligned} \iota _V : {\mathscr {P}}({\mathcal {N}})\rightarrow {\mathscr {P}}({\varvec{C}}) ; \quad {\varvec{u}}\mapsto \iota _V({\varvec{u}})=\varrho =\rho \;\!\mathrm {d}{\varvec{c}}\text { with } \rho ({\varvec{c}}) := V^I \sum _{{\varvec{n}}\in {\mathcal {N}}} u_{\varvec{n}}1\!\!1_{A_{\varvec{n}}^V}({\varvec{c}}), \end{aligned}$$

where $1\!\!1_A$ denotes the indicator function with $1\!\!1_A(b)=1$ for $b\in A$ and 0 otherwise. The corresponding dual operation acting on functions $\xi \in {\mathrm {C}}_{\mathrm {b}}({\varvec{C}})$ is given by

4.7 $$\begin{aligned} \iota _V^* : {\mathrm {C}}_{\mathrm {b}}({\varvec{C}}) \rightarrow \ell ^\infty ({\mathcal {N}}); \quad (\iota _V^* \xi )({\varvec{n}}) = V^I \int _{{\varvec{c}}\in A^V_{\varvec{n}}} \xi ({\varvec{c}})\;\!\mathrm {d}{\varvec{c}}. \end{aligned}$$

The final convergence result will be formulated in Theorem 4.7, which will be a direct consequence of the following three estimates

$$\begin{aligned} \begin{array}{lclc} \hbox {Section }4.3&{} \iota _V({\varvec{u}}^V) \overset{*}{\rightharpoonup }\varrho &{} \Rightarrow &{} {\varvec{E}}(\varrho ) \le \liminf \limits _{V\rightarrow \infty } {\mathcal {E}}_V({\varvec{u}}^V);\\ \hbox {Section }4.4&{} \iota _V({\varvec{u}}^V) \overset{*}{\rightharpoonup }\varrho &{} \Rightarrow &{} \Psi ^*_\mathrm {Lio}(\varrho ,{\mathrm {D}}{\varvec{E}}(\varrho )) \le \liminf \limits _{V\rightarrow \infty } \Psi _V^*({\varvec{u}}^V,{\mathrm {D}}{\mathcal {E}}_V({\varvec{u}}^V)); \\ \hbox {Section }4.5&{} \iota _V({\varvec{u}}^V)\overset{*}{\rightharpoonup }\varrho ,\ \xi \in {\mathrm {C}}^1_{\mathrm {c}}({\varvec{C}}) &{} \Rightarrow &{} \Psi ^*_\mathrm {Lio}(\varrho ,\xi ) \ge \limsup \limits _{V\rightarrow \infty } \Psi ^*_V({\varvec{u}}^V,\iota _V^*\xi ); \end{array} \end{aligned}$$

where the dual dissipation potential $\Psi ^*_\mathrm {Lio}$ is defined via

$$\begin{aligned} \Psi ^*_\mathrm {Lio}(\varrho , \xi ) = \frac{1}{2}\int _{\varvec{C}}\nabla \xi ({\varvec{c}}) \cdot {\mathbb {K}}({\varvec{c}}) \nabla \xi ({\varvec{c}}) \;\!\mathrm {d}\varrho ({\varvec{c}}). \end{aligned}$$

We will see in Sect. 4.6 that the limsup estimate for the dual potential $\Psi ^*_V$ in Sect. 4.5 provides a weak form of a liminf estimate for the primal potential $\Psi _V$.

A fundamental fact of the chosen gradient structures of the underlying Markov processes is that all the three terms in the energy-dissipation principle define convex functionals, which is of considerable help in proving the desired liminf estimates. Note that the convergence $\iota _V({\varvec{u}}^V) \overset{*}{\rightharpoonup }\varrho$ is rather weak. However, we can use that the coefficients of the transition rates defining the CME are quite regular, so that the other parts in the integral converge in a much better sense. Moreover, the functionals $\varrho \mapsto {\varvec{E}}(\varrho )$ and $\varrho \mapsto \Psi ^*_\mathrm {Lio}(\varrho ,{\mathrm {D}}{\varvec{E}}(\varrho ))$ are in fact linear in $\varrho$.

### $\Gamma$-Limit of the Relative Entropies

We also define ${\varvec{X}}_V:=\iota _V({\mathscr {P}}({\mathcal {N}})) \subset {\mathscr {P}}({\varvec{C}})$ and $W_V = \iota _V({\varvec{w}}^V) \in {\varvec{X}}_V$ and consider the functionals

$$\begin{aligned} \widehat{\mathcal {E}}_V : {\mathscr {P}}({\varvec{C}}) \rightarrow [0,\infty ], \qquad \widehat{\mathcal {E}}_V(\varrho )=\left\{ \begin{array}{cl} \widetilde{\mathcal {E}}_V(\varrho )&{} \text { if }\varrho \in {\varvec{X}}_V,\\ \infty &{}\text { otherwise}, \end{array} \right. \end{aligned}$$

where $\widetilde{\mathcal {E}}_V : {\mathscr {P}}({\varvec{C}}) \rightarrow [0,\infty ]$ is defined via

$$\begin{aligned} \widetilde{\mathcal {E}}_V(\varrho ) = \frac{1}{V} \text {Ent}(\varrho | W_V{\mathrm {d}}{\varvec{c}}) = \left\{ \begin{array}{cl} \frac{1}{V} \int _{\varvec{C}}\lambda _{\mathrm {B}}(\rho /W_V) W_V \;\!\mathrm {d}{\varvec{c}}&{} \text {for }\varrho =\rho \;\!\mathrm {d}c, \\ \infty &{}\text {otherwise} . \end{array}\right. \end{aligned}$$

These definitions are chosen such that ${\mathcal {E}}_V({\varvec{u}})= \widetilde{\mathcal {E}}_V(\iota _V({\varvec{u}}))= \widehat{\mathcal {E}}_V(\iota _V({\varvec{u}}))$ for all ${\varvec{u}}\in {\mathscr {P}}({\mathcal {N}})$.

Finally we define a natural inverse of $\iota _V$, namely

4.8 $$\begin{aligned} \varkappa _V:\ {\mathscr {P}}({\varvec{C}})\rightarrow {\mathscr {P}}({\mathcal {N}});\ \varrho \mapsto \Big ( \varrho \big ( A_{\varvec{n}}^V \big ) \Big )_{{\varvec{n}}\in {\mathcal {N}}}, \end{aligned}$$

such that $P_V := \iota _V \circ \varkappa _V$ is a projection from ${\mathscr {P}}({\varvec{C}})$ onto ${\varvec{X}}_V \subset {\mathscr {P}}({\varvec{C}})$.

To understand the limit of $\widehat{\mathcal {E}}_V$ for $V\rightarrow \infty$ we will use the representation

4.9 $$\begin{aligned} \widetilde{\mathcal {E}}_V(\rho {\mathrm {d}}{\varvec{c}})&=\frac{1}{V}\int _{\varvec{C}}\lambda _{\mathrm {B}}(\rho /W_V) W_V \;\!\mathrm {d}{\varvec{c}}= \int _{\varvec{C}}\Big (\frac{1}{V} \rho \log \rho + \rho E_V({\varvec{c}}) \Big ) \;\!\mathrm {d}{\varvec{c}}\nonumber \\&\quad \text {with }\ \ E_V({\varvec{c}})= \frac{1}{V} \log \Big ( \frac{1}{W_V({\varvec{c}})}\Big ) = - I\,\frac{\log V}{V} - \frac{1}{V} \log {\varvec{w}}^V_{\varvec{n}}\ \ \text { for } {\varvec{c}}\in A^V_{\varvec{n}}. \end{aligned}$$

In Lemma 4.2 below we will show that $E_V$ converges pointwise to *E* as defined in (2.9). To quantify the latter convergence, we use the classical lower and upper bounds of [49] for Stirling’s formula:

4.10 $$\begin{aligned} \begin{aligned} \forall \, n\in {\mathbb {N}}_0 : \quad&n! = \sqrt{2\pi k_n} \Big (\frac{n}{{\mathrm {e}}}\Big )^n \quad \text {with }k_0=\frac{1}{2\pi }\\&\text {and } k_n=n+\frac{1}{6} +\frac{\gamma _n}{124/5+72 n} \text { with } \gamma _n\in [0.9,1] \text { for } n\ge 1. \end{aligned} \end{aligned}$$

Using this estimate and recalling *E* from (2.9) we obtain the following estimate.

#### Lemma 4.2

(Pointwise bound for $E_V$) For all ${\varvec{c}}^*>0$ there exist $K_*>0$ and $V_*>0$ such that for all $V\ge V_*$ the following bounds hold:

4.11 $$\begin{aligned} | E_V({\varvec{c}}) - E({\varvec{c}}) | \le \frac{K_*}{V} \big (\log V + E({\varvec{c}})\big ) \,\text { for all }{\varvec{c}}\in {\varvec{C}}. \end{aligned}$$

#### Proof

We decompose the error via

4.12 $$\begin{aligned} E_V({\varvec{c}})-E({\varvec{c}})=\bigg (E_V({\varvec{c}}){-} E\bigg (\frac{1}{V} {\varvec{n}}\bigg )\bigg ) + \bigg (E\bigg (\frac{1}{V} {\varvec{n}}\bigg ){-} E({\varvec{c}}) \bigg ) \end{aligned}$$

with ${\varvec{n}}$ defined by ${\varvec{c}}\in A_{\varvec{n}}^V$. For the second term we use the convexity of $\lambda _{\mathrm{B}}$ and the estimate $\log z \le 1+\lambda _{\mathrm {B}}(z)$. Hence, we have

$$\begin{aligned} \begin{aligned} c_i^* \Bigg [ \lambda _{\mathrm {B}}\bigg (\frac{c_i}{c_i^*}\bigg ) - \lambda _{\mathrm {B}}\bigg (\frac{n_i}{V c_i^*}\bigg ) \Bigg ]&\le \bigg (c_i {-} \frac{n_i}{V}\bigg ) \log \bigg (\frac{c_i}{c_i^*}\bigg ) \le \frac{1}{V}\Big (1+\lambda _{\mathrm {B}}\bigg (\frac{c_i}{c^*_i} \bigg ) \Big ) \\&\le \frac{\max \{I,1/c^*_i\}}{V} \Big (\frac{1}{I} +c^*_i\lambda _{\mathrm {B}}\bigg (\frac{c_i}{c^*_i} \bigg ) \Big ). \end{aligned} \end{aligned}$$

Summing this inequality over $i=1, \ldots ,I$ we obtain the upper bound

4.13 $$\begin{aligned} E({\varvec{c}}) - E\big (\tfrac{1}{V} {{\varvec{n}}}\big ) \le \frac{K_1}{V} \big (1 {+} E({\varvec{c}})\big ) \,\text { with }K_1=\max \{I, 1/c^*_1, ... ,1/c^*_I\}. \end{aligned}$$

For the opposite direction we use (a) that $\lambda _{\mathrm {B}}$ decreases on [0, 1] and the convexity of $\lambda _{\mathrm {B}}$ which implies (b) $\lambda _{\mathrm {B}}(z_1)-\lambda _{\mathrm {B}}(z_2)\le \lambda _{\mathrm {B}}(0)-\lambda _{\mathrm {B}}(z_2{-}z_1)$ for $0 \le z_1 \le z_2$. This yields

4.14 $$\begin{aligned} \begin{aligned} E\big (\tfrac{1}{V} {{\varvec{n}}}\big ) - E({\varvec{c}})&= \sum _{i=1}^I c_i^* \Bigg [\lambda _{\mathrm{B}}\bigg (\frac{n_i}{Vc_i^*}\bigg ) - \lambda _{\mathrm{B}}\bigg (\frac{c_i}{c_i^*}\bigg ) \Bigg ] \;\overset{\text {(b)}}{\le }\; \sum _{i=1}^I c_i^* \Bigg [\lambda _{\mathrm{B}}(0) - \lambda _{\mathrm{B}}\bigg (\frac{c_i}{c_i^*} {-} \frac{n_i}{Vc_i^*}\bigg ) \Bigg ] \\&\overset{\text {(a)}}{\le }\, \sum _{i=1}^I c_i^* \bigg [\lambda _{\mathrm{B}}(0) - \lambda _{\mathrm{B}} \bigg (\tfrac{1}{Vc_i^*}\bigg ) \bigg ] = \frac{1}{V} \sum _{i=1}^I \big ( 1 {+} \log (V c_i^*)\big ) \, \overset{\text {(c)}}{\le }\, 2I\, \frac{\log V}{V}, \end{aligned} \end{aligned}$$

if $V\ge V_1^*:= \max \big \{\, \max \{1/c^*_i, {\mathrm {e}}c^*_i \} \, \big | \, i=1,...,I \,\big \}$, where $Vc^*_i\ge 1$ and $V\ge {\mathrm {e}}c^*_i$ are needed in (a) and (c), respectively. Together with (4.13) this controls the second error term in (4.12), viz.

4.15 $$\begin{aligned} \big |E({\varvec{c}})- E\big ( \tfrac{1}{V} {\varvec{n}}) \big | \le \frac{K_2}{V} \big ( \log V + E({\varvec{c}})\big ) \ \text { for } V\ge V^*_2 = \max \{ {\mathrm {e}}, V^*_1\}, \end{aligned}$$

where $$K_2=\max \{2I, K_1 \}$$.

For controlling the first error term in (4.12) we use (4.9) and obtain the identity

4.16 $$\begin{aligned} E_V({\varvec{c}}) - E\big (\tfrac{1}{V}{ {\varvec{n}}}\big ) = - I \frac{\log V}{V} + \frac{1}{2V} \sum _{i=1}^I \log (2\pi k_{n_i}) \quad \text {for all } {\varvec{c}}\in A^V_{\varvec{n}}, \end{aligned}$$

with $k_n$ from (4.10). Because of $2\pi k_n\ge 1$ we obtain, for all $V\ge 1$, the lower bound

$$\begin{aligned} E_V({\varvec{c}}) - E\big (\tfrac{1}{V}{ {\varvec{n}}}\big ) \ge - I \frac{\log V}{V} \ge - \frac{I}{V} \big ( \log V +E({\varvec{c}})\big ). \end{aligned}$$

For the upper bound we use $2\pi k_0=1$ and $2\pi k_n \le 8 n$ for $n \ge 1$. Hence for $n_i \ge 1$ we obtain, using again the estimate $\log z \le 1+\lambda _{\mathrm {B}}(z)$,

$$\begin{aligned} \log (2\pi k_{n_i}) \le \log (8n_i) \le \log (8c^*_i V) + \log \big (\frac{c_i}{c^*_i}\big ) \le \log V + \log (8 {\mathrm {e}}c^*_i) + \frac{1}{c^*_i} c^*_i\lambda _{\mathrm {B}}\big ( \frac{c_i}{c^*_i}\big ). \end{aligned}$$

Summation over $i=1,\ldots ,I$ yields, for all ${\varvec{c}}\in A^V_{\varvec{n}}$ and $V \ge V_3^*:= 8{\mathrm {e}}\max \{c^*_1,\ldots ,c^*_I\}$, the upper bound

$$\begin{aligned} E_V({\varvec{c}}) - E\big (\frac{ {\varvec{n}}}{V}\big ) \le \frac{K_3}{V} E({\varvec{c}}) \,\text { with } K_3 = \max \Big \{ \frac{1}{2c^*_1},\ldots ,\frac{1}{2c^*_I} \Big \}. \end{aligned}$$

Together with the lower estimate we control the first error term in (4.12) via

$$\begin{aligned} \big | E_V({\varvec{c}}) - E(\tfrac{1}{V} {\varvec{n}})| \le \frac{K_4}{V} \big ( \log V + E({\varvec{c}})\big ) \ \text {for } V \ge V^*_4=\max \{1, V^*_3\}, \end{aligned}$$

where $$K_4=\max \{I,K_3\}$$.

Adding the estimates for first and the second error term (4.12) we obtain the desired estimate (4.11) with the choices $K_*=K_2+ K_4$ and $V_*=\max \{V_2^*,V_4^* \}$. $\square$

For consistency of notation we remark that $\widetilde{\mathcal {E}}_V(\varrho )$ can be rewritten as

$$\begin{aligned} \widetilde{\mathcal {E}}_V(\rho {\mathrm {d}}{\varvec{c}}) =\int _{\varvec{C}}\big (\frac{1}{V} \log \rho ({\varvec{c}}) + E_V({\varvec{c}}) \big ) \rho ({\varvec{c}}) \;\!\mathrm {d}{\varvec{c}}\;, \end{aligned}$$

provided that this integral exists. The limit functional ${\varvec{E}}$ is given by

4.17 $$\begin{aligned} {\varvec{E}}:{\mathscr {P}}({\varvec{C}})\rightarrow [0,\infty ]; \quad \varrho \mapsto \int _{\varvec{C}}E({\varvec{c}}) \;\!\mathrm {d}\varrho ({\varvec{c}}), \end{aligned}$$

where we use that *E* is a continuous and non-negative function, so that ${\varvec{E}}$ can be defined everywhere but attains the value $+\infty$ if $\varrho$ does not decay suitably at infinity. We will use the following semi-continuity result.

#### Lemma 4.3

(Lower semi-continuity of ${\varvec{E}}$) For sequences $(\varrho _k)_k \subset {\mathscr {P}}({\varvec{C}})$ with $\varrho _k \overset{*}{\rightharpoonup }\varrho _\infty$, we have ${\varvec{E}}(\varrho _\infty ) \le \liminf _{k\rightarrow \infty }{\varvec{E}}(\varrho _k)$.

#### Proof

For cut-off functions $\chi \in {\mathrm {C}}_{\mathrm {c}}({\varvec{C}})$ with $\chi ({\varvec{c}}) \in [0,1]$ we have ${\varvec{E}}(\chi \varrho _k)\rightarrow {\varvec{E}}(\chi \varrho _\infty )$ by weak* convergence and continuity of *E*. Using $\chi \le 1$ yields ${\varvec{E}}(\chi \varrho _\infty ) \le \liminf _{k\rightarrow \infty } {\varvec{E}}(1 \varrho _k)$. Choosing a non-decreasing sequence $\chi _n$ with $\chi _n({\varvec{c}})\rightarrow 1$ for all ${\varvec{c}}\in {\varvec{C}}$ we have ${\varvec{E}}(\chi _n \varrho _\infty ) \rightarrow {\varvec{E}}(1 \varrho _\infty )$ by Beppo Levi’s monotone convergence, and the assertion follows. $\square$

The following result gives the $\Gamma$-convergence of ${\mathcal {E}}_V$ to ${\varvec{E}}$ with respect to the sequential weak* convergence as well as the equi-coercivity.

#### Theorem 4.4

($\Gamma$-convergence of ${\mathcal {E}}_V$ to ${\varvec{E}}$) Let $\widehat{\mathcal {E}}_V$ and ${\varvec{E}}$ be defined on ${\mathscr {P}}({\varvec{C}})$ as above. Then we have the following properties:

- *Compactness / equi-coercivity:*
  4.18 $$\begin{aligned} \exists \, V_*,C, c>0\ \forall \, V\ge V_*\ \forall \, \varrho \in {\mathscr {P}}({\varvec{C}}): \quad \widehat{\mathcal {E}}_V(\varrho ) \ge -C + c {\varvec{E}}(\varrho ). \end{aligned}$$
- Weak* liminf estimate:
  4.19 $$\begin{aligned} \varrho _V \overset{*}{\rightharpoonup }\varrho \text { in } {\mathscr {P}}({\varvec{C}}) \quad \Longrightarrow \quad \liminf _{V\rightarrow \infty } \widehat{\mathcal {E}}_V(\varrho _V) \ge {\varvec{E}}(\varrho ). \end{aligned}$$
- *Limsup estimate / recovery sequence:*
  4.20 $$\begin{aligned} \forall \, \widehat{\varrho }\in {\mathscr {P}}({\varvec{C}}) \ \exists \, (\widehat{\varrho }_V)_{V\ge 1}: \quad \widehat{\mathcal {E}}_V(\widehat{\varrho }_V) \rightarrow {\varvec{E}}(\widehat{\varrho }) \text { and } \widehat{\varrho }_V \overset{*}{\rightharpoonup }\widehat{\varrho }, \end{aligned}$$
  where we may take $\widehat{\varrho }_V= P_V \widehat{\varrho }= \iota _V\big ( \varkappa _V(\widehat{\varrho })\big )$.

#### Proof

Obviously it is sufficient to show the lower bound (a) and the liminf estimate (b) for the smaller functional $\widetilde{\mathcal {E}}_V$, and for $\varrho = \rho \;\!\mathrm {d}{\varvec{c}}$ with $\rho \in {\mathrm {L}}^1({\varvec{C}})$ (resp. $\varrho _V = \rho _V \;\!\mathrm {d}{\varvec{c}}$ with $\rho _V \in {\mathrm {L}}^1({\varvec{C}})$). We use the elementary convexity estimate

$$\begin{aligned} \forall \, r\ge 0,\ a,w>0: \quad w\lambda _{\mathrm {B}}(r/w) = r\log (r/w) - r + w \ge r \log (a/w) -a + w. \end{aligned}$$

We choose $r({\varvec{c}})=\varrho ({\varvec{c}})$, $w({\varvec{c}})=W_V({\varvec{c}})$, and $a({\varvec{c}}) = {\mathrm {e}}^{-|{\varvec{c}}|_1} = \Pi _{i=1}^I {\mathrm {e}}^{-c_i} >0$. Note that $a\in {\mathrm {L}}^\infty ({\varvec{C}})\cap {\mathscr {P}}({\varvec{C}})$ and $W_V/a$ is bounded from above, for any fixed *V*. Hence, ${\varvec{c}}\mapsto \log (a({\varvec{c}}) / W_V({\varvec{c}}))= -|{\varvec{c}}|_1 + V E_V({\varvec{c}})$ is bounded from below, and we can integrate the above estimate to obtain the lower bound

$$\begin{aligned} \widetilde{\mathcal {E}}_V(\varrho ) \ge \frac{1}{V}\int _{\varvec{C}}\log \big ( a({\varvec{c}})/W_V({\varvec{c}})\big ) \;\!\mathrm {d}\varrho ({\varvec{c}}) = \int _{\varvec{C}}\Big (E_V({\varvec{c}}) - \frac{|{\varvec{c}}|_1}{V} \Big ) \;\!\mathrm {d}\varrho ({\varvec{c}}). \end{aligned}$$

Since there exists a constant $K_1 > 0$ such that $|{\varvec{c}}|_1\le K_1\big (1{+}E({\varvec{c}})\big )$ and since $E_V$ satisfies the lower bound in (4.11), we obtain the lower bound

$$\begin{aligned} \widetilde{\mathcal {E}}_V(\varrho )&\ge \int _{\varvec{C}}E({\varvec{c}}) \;\!\mathrm {d}\varrho ({\varvec{c}}) - \frac{K_*{+}K_1}{V} \int _{\varvec{C}}\big (\log V {+}E({\varvec{c}})\big )\;\!\mathrm {d}\varrho ({\varvec{c}}) \\&= {\varvec{E}}(\varrho ) - \frac{K_*{+}K_1}{V}\big ( \log V + {\varvec{E}}(\varrho )\big ). \end{aligned}$$

This immediately implies (4.18) in part (a) with $V_* / \log V_* =2(K_*{+}K_1)$. Moreover, if $\varrho _V \overset{*}{\rightharpoonup }\varrho$ then we have the lower bound $\widehat{\mathcal {E}}_V(\varrho _V) \ge {\varvec{E}}(\varrho _V) - \frac{K_*{+}K_1}{V} \big ( \log V + {\varvec{E}}(\varrho _V)\big )$ and the liminf estimate (4.19) follows from Lemma 4.3.

To show part (c) we use the indicated recovery sequence and the upper bounds for $E_V$ from (4.11). For a given $\widehat{\varrho }\in {\mathscr {P}}({\varvec{C}})$ we define $\widehat{\varrho }_V=\iota _V(\varkappa _V(\widehat{\varrho }))$. For an arbitrary continuous and bounded test function $\psi$ we define the piecewise constant approximation $\psi _V$ via averaging over $A_{\varvec{n}}^V$. We obtain

$$\begin{aligned} \int _{\varvec{C}}\psi ({\varvec{c}})\;\!\mathrm {d}\widehat{\varrho }_V ({\varvec{c}}) = \int _{\varvec{C}}\psi _V ({\varvec{c}})\;\!\mathrm {d}\widehat{\varrho }_V ({\varvec{c}}) = \int _{\varvec{C}}\psi _V ({\varvec{c}})\;\!\mathrm {d}\widehat{\varrho }({\varvec{c}}) \rightarrow \int _{\varvec{C}}\psi ({\varvec{c}}) \;\!\mathrm {d}\widehat{\varrho }({\varvec{c}}), \end{aligned}$$

where the convergence follows via Lebesgue’s dominated convergence from the pointwise convergence $\psi _V \rightarrow \psi$ and the uniform boundedness of $\psi _V$. Thus, we conclude $\widehat{\varrho }_V\overset{*}{\rightharpoonup }\widehat{\varrho }$.

To show convergence of $$\widehat{\mathcal {E}}_V(\widehat{\varrho }_V)$$ it suffices to prove the upper bound $$\limsup _{V\rightarrow \infty } \widehat{\mathcal {E}}_V(\widehat{\varrho }_V) $$$$\le {\varvec{E}}(\widehat{\varrho })$$. For this we use the bound $$\widehat{\rho }_V({\varvec{c}})\le V^I=1/\text {vol}(A^V_{\varvec{n}}) $$ and the fact that $$\widehat{\rho }_V$$ and $$E_V$$ are constant on the same cubes to obtain

$$\begin{aligned} \widehat{\mathcal {E}}_V(\widehat{\varrho }_V) = \int _{\varvec{C}}\left( \frac{\log \widehat{\rho }_V({\varvec{c}})}{V} + E_V({\varvec{c}})\right) \;\!\mathrm {d}\widehat{\rho }_V({\varvec{c}}) \le \frac{I\log V}{V} + \int _{\varvec{C}}E_V({\varvec{c}}) \;\!\mathrm {d}\widehat{\varrho }({\varvec{c}}), \end{aligned}$$

where now only the measure $\widehat{\varrho }$ is left. The first term tends to 0 for $V\rightarrow \infty$, and the second can be estimated from above using the upper estimate in (4.11), which yields

$$\begin{aligned} \textstyle \widehat{\mathcal {E}}_V(\widehat{\varrho }_V)&\le \frac{I\log V}{V} + \int _{\varvec{C}} E({\varvec{c}}) + \frac{K_*}{V} \big (\log V {+} E({\varvec{c}})\big ) \;\!\mathrm {d}\widehat{\varrho }({\varvec{c}}) \\&= \bigg (1 + \frac{K_*}{V}\bigg ) {\varvec{E}}(\widehat{\varrho }) + \frac{I{+} K_*}{V}\log V . \end{aligned}$$

This implies the desired upper bound for $V\rightarrow \infty$, and the proof is complete. $\square$

### A Liminf Estimate for the Dual Dissipation Functional

Here we provide the liminf estimate for the dual dissipation potential $\Psi _V^*({\varvec{u}}^V,{\mathrm {D}}{\mathcal {E}}_V({\varvec{u}}^V))$ based on the lower bound

4.21 $$\begin{aligned} \Psi ^*_\mathrm {Lio}(\varrho ,{\mathrm {D}}{\varvec{E}}(\varrho )) = \frac{1}{2} \int _{\varvec{C}}\nabla E({\varvec{c}})\cdot {\mathbb {K}}({\varvec{c}}) \nabla E({\varvec{c}}) \;\!\mathrm {d}\varrho ({\varvec{c}}). \end{aligned}$$

We observe that the latter term is linear in $\varrho$ while the former term is convex in ${\varvec{u}}^V$. Indeed, introducing the convex function $G(a,b)=(a{-}b)(\log a - \log b)$ for $a,b>0$ and noting the relation $\Lambda (a,b)(\log a - \log b)^2 = G(a,b)$ we have

4.22 $$\begin{aligned} \Psi _V^* \big ( {\varvec{u}}^V,{\mathrm {D}}{\mathcal {E}}_V({\varvec{u}}^V) \big ) = \frac{1}{ 2 V} \sum _{r=1}^R \sum _{n\in {\mathcal {N}}} \widehat{\nu }^{{\varvec{n}},r}_V G \Bigg ( \frac{u_{{\varvec{n}}+{\varvec{\alpha }}^r}^V}{w^V_{{\varvec{n}}+{\varvec{\alpha }}^r}}, \frac{u_{{\varvec{n}}+{\varvec{\beta }}^r}^V}{w^V_{{\varvec{n}}+{\varvec{\beta }}^r}} \Bigg ). \end{aligned}$$

To establish the linear lower bound we use the elementary, affine lower bound

4.23 $$\begin{aligned} \forall \, a,b >0, \omega \in {\mathbb {R}}: \quad G(a,b) \ge g(\omega ) \, a + g(-\omega )\, b, \quad \text {where } g(\omega ) := 1-{\mathrm {e}}^{-\omega } + \omega . \end{aligned}$$

This estimate follows easily by convexity, $G(a,b)\ge G({\mathrm {e}}^\omega ,1)+{\mathrm {D}}G({\mathrm {e}}^\omega ,1)\cdot (a{-}{\mathrm {e}}^\omega ,b{-}1)$, and 1-homogeneity giving $G({\mathrm {e}}^\omega ,1)={\mathrm {D}}G({\mathrm {e}}^\omega ,1)\cdot ({\mathrm {e}}^\omega ,1)$. Note that equality holds in (4.23) if $\omega = \log (a/b)$. Moreover, we have $g(\omega ) + g(-\omega ) = 2-{\mathrm {e}}^\omega -{\mathrm {e}}^{-\omega } \le 0$, so a careful choice of $\omega$ depending on ${\varvec{n}}$ will be necessary to obtain a good lower bound with a positive leading term.

#### Proposition 4.5

We have the liminf estimate

$$\begin{aligned} \iota _V({\varvec{u}}^V) \overset{*}{\rightharpoonup }\varrho \text { in } {\mathscr {P}}({\varvec{C}}) \quad \Longrightarrow \quad \Psi ^*_\mathrm {Lio}(\varrho ,{\mathrm {D}}{\varvec{E}}(\varrho )) \le \liminf _{V\rightarrow \infty } \Psi ^*_V({\varvec{u}}^V,{\mathrm {D}}{\mathcal {E}}_V({\varvec{u}}^V)). \end{aligned}$$

#### Proof

The special forms of ${\mathbb {K}}(c)$, $E({\varvec{c}})$, and $\Psi ^*_\mathrm {Lio}$ in (4.21) give the formula

4.24 $$\begin{aligned} \Psi ^*_\mathrm {Lio}(\varrho ,{\mathrm {D}}{\varvec{E}}(\varrho )) = \frac{1}{2} \int _{\varvec{C}}\sum _{r=1}^R\,\kappa _*^r \,G\Bigg ( \frac{{\varvec{c}}^{{\varvec{\alpha }}^r}}{{\varvec{c}}_*^{{\varvec{\alpha }}^r}} \, , \, \frac{{\varvec{c}}^{{\varvec{\beta }}^r}}{{\varvec{c}}_*^{{\varvec{\beta }}^r}} \Bigg ) \;\!\mathrm {d}\varrho ({\varvec{c}}) . \end{aligned}$$

Since $\Psi _V^*$ and $\Psi ^*_\mathrm {Lio}$ are defined as sums over $r=1,\ldots ,R$ of nonnegative terms, it suffices to show the result for each *r* separately, where we suppress the index *r*.

Inserting (4.23) into (4.22) yields, with $\omega _{\varvec{n}}\in {\mathbb {R}}$ to be fixed afterwards,

$$\begin{aligned} \Psi _V^*({\varvec{u}}^V,{\mathrm {D}}{\mathcal {E}}_V({\varvec{u}}^V))&\ge \frac{1}{2V} \sum _{{\varvec{n}}\in {\mathcal {N}}} \widehat{\nu }^{{\varvec{n}}}_V \,\Bigg (g(\omega _{\varvec{n}}) \frac{u_{{\varvec{n}}+{\varvec{\alpha }}}^V}{w^V_{{\varvec{n}}+{\varvec{\alpha }}}} +g(-\omega _{\varvec{n}}) \frac{u_{{\varvec{n}}+{\varvec{\beta }}}^V}{w^V_{{\varvec{n}}+{\varvec{\beta }}}} \Bigg ) \\&= \frac{\kappa _*}{2} \sum _{{\varvec{n}}\in {\mathcal {N}}} \Big (g(\omega _{\varvec{n}}) {{\mathbb {A}}}^{\varvec{\alpha }}_V({\varvec{n}}) u_{{\varvec{n}}+{\varvec{\alpha }}}^V + g(-\omega _{\varvec{n}}) {{\mathbb {A}}}^{\varvec{\beta }}_V({\varvec{n}}) u_{{\varvec{n}}+{\varvec{\beta }}}^V \Big ) \\&\quad \ \text { with } {{\mathbb {A}}}^{\varvec{\delta }}_V({\varvec{n}}) := \frac{w_{{\varvec{n}}}^V}{w_{{\varvec{n}}+{\varvec{\delta }}}^V} = \frac{({\varvec{n}}{+} {\varvec{\delta }})!}{ ({\varvec{c}}_* V)^{\varvec{\delta }}{\varvec{n}}!}, \end{aligned}$$

where we used the detailed-balance conditions from Theorem 3.1 for the last identity. Rearranging the sum and recalling that ${{\mathbb {A}}}^\delta ({\varvec{n}})=0$ for ${\varvec{n}}\not \in {\mathcal {N}}$ we find

$$\begin{aligned} \Psi _V^*({\varvec{u}}^V,{\mathrm {D}}{\mathcal {E}}_V({\varvec{u}}^V))\ge & {} \frac{\kappa _*}{2}\sum _{{\varvec{n}}\in {\mathcal {N}}} h^V_{\varvec{n}}u_{\varvec{n}}^V \quad \text {with } h^V_{\varvec{n}}:= g(\omega _{{\varvec{n}}-{\varvec{\alpha }}}) {{\mathbb {A}}}^{\varvec{\alpha }}_V({\varvec{n}}-{\varvec{\alpha }}) \\&+ g(-\omega _{{\varvec{n}}-{\varvec{\beta }}}) {{\mathbb {A}}}^{\varvec{\beta }}_V({\varvec{n}}-{\varvec{\beta }}). \end{aligned}$$

We now choose $\omega _{\varvec{n}}=\log \big ( {{\mathbb {A}}}^{\varvec{\alpha }}_V({\varvec{n}}) / {{\mathbb {A}}}^{\varvec{\beta }}_V({\varvec{n}}) \big )$ for ${\varvec{n}}\in {\mathcal {N}}$ and $\omega _{\varvec{n}}=0$ otherwise and find, for all ${\varvec{n}}$ with ${\varvec{n}}\ge {\varvec{\alpha }}$ or ${\varvec{n}}\ge {\varvec{\beta }}$, the relation

$$\begin{aligned} h^V_{\varvec{n}}&= G\big ({{\mathbb {A}}}^{\varvec{\alpha }}_V({\varvec{n}}{-} {\varvec{\alpha }}), {{\mathbb {A}}}^{\varvec{\beta }}_V({\varvec{n}}{-} {\varvec{\beta }}) \big ) + f^V_{\varvec{n}}\quad \text {with } \\ f^V_{\varvec{n}}&:= {{\mathbb {A}}}^{\varvec{\alpha }}_V({\varvec{n}}{-} {\varvec{\alpha }}) - {{\mathbb {A}}}^{\varvec{\alpha }}_V({\varvec{n}}{-} {\varvec{\beta }}) + {{\mathbb {A}}}^{\varvec{\beta }}_V({\varvec{n}}{-} {\varvec{\beta }}) - {{\mathbb {A}}}^{\varvec{\beta }}_V({\varvec{n}}{-} {\varvec{\alpha }}) \\&\quad + {{\mathbb {A}}}^{\varvec{\alpha }}_V({\varvec{n}}{-} {\varvec{\alpha }}) \log \bigg ( \frac{{{\mathbb {A}}}^{\varvec{\beta }}_V({\varvec{n}}{-} {\varvec{\beta }})}{{{\mathbb {A}}}^{\varvec{\beta }}_V({\varvec{n}}{-} {\varvec{\alpha }})} \bigg ) + {{\mathbb {A}}}^{\varvec{\beta }}_V({\varvec{n}}{-} {\varvec{\beta }}) \log \bigg ( \frac{{{\mathbb {A}}}^{\varvec{\alpha }}_V({\varvec{n}}{-} {\varvec{\alpha }})}{{{\mathbb {A}}}^{\varvec{\alpha }}_V({\varvec{n}}{-} {\varvec{\beta }})} \bigg ) \end{aligned}$$

The idea is now that as $\frac{1}{V} {\varvec{n}}\rightarrow {\varvec{c}}> \varvec{0}$ we have the convergences

$$\begin{aligned} {{\mathbb {A}}}^{\varvec{\delta }}_V({\varvec{n}}{-} {\varvec{\alpha }}) \rightarrow {\varvec{c}}^{\varvec{\delta }}/{\varvec{c}}_*^{\varvec{\delta }}\quad \text {and} \quad {{\mathbb {A}}}^{\varvec{\delta }}_V({\varvec{n}}{-} {\varvec{\beta }}) \rightarrow {\varvec{c}}^{\varvec{\delta }}/{\varvec{c}}_*^{\varvec{\delta }}, \end{aligned}$$

which yields $f^V_{\varvec{n}}\rightarrow 0$ and $h^V_{\varvec{n}}\rightarrow G({\varvec{c}}^{\varvec{\alpha }}/ {\varvec{c}}_*^{\varvec{\alpha }}, {\varvec{c}}^{\varvec{\beta }}/{\varvec{c}}_*^{\varvec{\beta }})$ as desired. To be more precise we define, for all $\varepsilon \in {]0,1[}$, the functions

$$\begin{aligned} G_\varepsilon (a,b)= -\varepsilon + \min \{ (1{-}\varepsilon )G(a,b), 1/\varepsilon \}, \end{aligned}$$

which converge monotonely to *G*(*a*, *b*) for $\varepsilon \searrow 0$. A lengthy calculation using the explicit structure of ${{\mathbb {A}}}^{\varvec{\delta }}({\varvec{n}})$ shows that for all $\varepsilon >0$ there exists $V_\varepsilon \gg 1$ such that $h^V_{\varvec{n}}\ge G_\varepsilon ({{\mathbb {A}}}^{\varvec{\alpha }}_V({\varvec{n}}),{{\mathbb {A}}}^{\varvec{\beta }}_V({\varvec{n}}))$ for all $V \ge V_\varepsilon$ and all ${\varvec{n}}$. Even more, if we define the functions $H_V:{\varvec{C}}\rightarrow {\mathbb {R}}; \ {\varvec{c}}\mapsto \sum _{{\varvec{n}}\in {\mathcal {N}}} h_{\varvec{n}}^V \,1\!\!1_{A^V_{\varvec{n}}}({\varvec{c}})$, then, for all $\varepsilon >0$ there exists $\widetilde{V}_\varepsilon \gg 1$ such that

$$\begin{aligned} \forall \,V\ge \widetilde{V}_\varepsilon \ \forall \, {\varvec{c}}\in {\varvec{C}}:\ H_V({\varvec{c}})\ge {\mathfrak {H}}_\varepsilon ({\varvec{c}}):= G_\varepsilon \Big ( \frac{{\varvec{c}}^{\varvec{\alpha }}}{{\varvec{c}}_*^{\varvec{\alpha }}}, \frac{{\varvec{c}}^{\varvec{\beta }}}{{\varvec{c}}_*^{\varvec{\beta }}} \Big ). \end{aligned}$$

Hence, using the definition of $\iota _V$ we find the lower bound

$$\begin{aligned} \Psi ^*_V({\varvec{u}}^V,{\mathrm {D}}{\mathcal {E}}_V({\varvec{u}}^V))\ge & {} \frac{\kappa _*}{2}\sum _{\varvec{n}}h^V_{\varvec{n}}u_{\varvec{n}}^V = \frac{\kappa _*}{2}\int _{\varvec{C}}H_V({\varvec{c}}) \;\!\mathrm {d}\iota _V({\varvec{u}}^V)({\varvec{c}}) \\\ge & {} \frac{\kappa _*}{2}\int _{\varvec{C}}{\mathfrak {H}}_\varepsilon ({\varvec{c}}) \;\!\mathrm {d}\iota _V({\varvec{u}}^V)({\varvec{c}}) . \end{aligned}$$

Since ${\mathfrak {H}}_\varepsilon$ is lower semi-continuous and bounded, this implies the liminf estimate

$$\begin{aligned} \iota _V({\varvec{u}}^V) \overset{*}{\rightharpoonup }\varrho \quad \Longrightarrow \quad \liminf _{V\rightarrow \infty } \Psi ^*_V\big ({\varvec{u}}^V,{\mathrm {D}}{\mathcal {E}}_V({\varvec{u}}^V)\big ) \ge \frac{\kappa _*}{2} \int _{\varvec{C}}{\mathfrak {H}}_\varepsilon ({\varvec{c}}) \;\!\mathrm {d}\varrho ({\varvec{c}}). \end{aligned}$$

Because $\varepsilon >0$ was arbitrary we can use the monotone convergence ${\mathfrak {H}}_\varepsilon ({\varvec{c}}) \nearrow G\big ( \tfrac{{\varvec{c}}^{\varvec{\alpha }}}{{\varvec{c}}_*^{\varvec{\alpha }}}, \tfrac{{\varvec{c}}^{\varvec{\beta }}}{{\varvec{c}}_*^{\varvec{\beta }}} \big )$ to conclude the desired result for each of the *R* reactions

$$\begin{aligned} \liminf _{V\rightarrow \infty } \Psi ^{r,*}_V({\varvec{u}}^V,{\mathrm {D}}{\mathcal {E}}_V({\varvec{u}}^V))&\ge \frac{\kappa _*}{2} \int _{\varvec{C}}G\big ( \tfrac{{\varvec{c}}^{{\varvec{\alpha }}^r}}{{\varvec{c}}_*^{{\varvec{\alpha }}^r}}, \tfrac{{\varvec{c}}^{{\varvec{\beta }}^r}}{{\varvec{c}}_*^{{\varvec{\beta }}^r}} \big ) \;\!\mathrm {d}\varrho ({\varvec{c}}) \\&= \frac{\kappa _*}{2} \int _{\varvec{C}}\Lambda \big ( \tfrac{{\varvec{c}}^{{\varvec{\alpha }}^r}}{{\varvec{c}}_*^{{\varvec{\alpha }}^r}}, \tfrac{{\varvec{c}}^{{\varvec{\beta }}^r}}{{\varvec{c}}_*^{{\varvec{\beta }}^r}} \big ) \big ( \nabla E({\varvec{c}}){\cdot } ({\varvec{\alpha }}^r-{\varvec{\beta }}^r)\big ) ^2 \;\!\mathrm {d}\varrho ({\varvec{c}}). \end{aligned}$$

Summation over $r=1,\ldots ,R$ yields the full result for $\Psi ^*_V$. $\square$

### A Liminf Estimate for the Dissipation Functional

In the evolutionary $\Gamma$-convergence method of [41, 54, 58] it is standard to provide a liminf estimate for the *primal dissipation potential* $\Psi _V$ which in our case is defined via the Legendre transform

$$\begin{aligned} \Psi _V({\varvec{u}},{\varvec{v}}) = \sup \Big \{\, {\textstyle \sum \limits _{{\varvec{n}}\in {\mathcal {N}}}} u_{\varvec{n}}\xi _{\varvec{n}}- \Psi ^*_V({\varvec{u}},{\varvec{\xi }}) \; \Big | \; {\varvec{\xi }}=(\xi _{\varvec{n}})_{{\varvec{n}}\in {\mathcal {N}}} \,\Big \} . \end{aligned}$$

However, as our theory relies on the dualization $\Psi _V({\varvec{u}},{\varvec{v}}) \ge \sum _{{\varvec{n}}\in {\mathcal {N}}} u_{\varvec{n}}\xi _{\varvec{n}}- \Psi _V^*({\varvec{u}},{\varvec{\xi }})$ it will be sufficient to have the following limsup estimate for $\Psi _V^*$, which crucially relies on the concavity of the map $(a,b)\mapsto \Lambda (a,b)$.

#### Proposition 4.6

Consider any pair $(\varrho ,\xi )\in {\mathscr {P}}({\varvec{C}}) {\times }{\mathrm {C}}^1_{\mathrm {c}}({\varvec{C}})$ and set ${\varvec{\xi }}^V=\iota _V^* \xi : {\mathcal {N}}\rightarrow {\mathbb {R}}$ with $\iota _V^*$ defined in (4.7). Then, for every family $({\varvec{u}}^V)_{V>1}$ we have the limsup estimate

4.25 $$\begin{aligned} \iota _V({\varvec{u}}^V)\overset{*}{\rightharpoonup }\varrho \quad \Longrightarrow \quad \limsup _{V\rightarrow \infty } \Psi _V^*({\varvec{u}}^V,{\varvec{\xi }}^V) \le \Psi _\mathrm {Lio}^*(\varrho ,\xi )=\frac{1}{2}\int _{\varvec{C}}\nabla \xi \cdot {\mathbb {K}}\nabla \xi \;\!\mathrm {d}\varrho ({\varvec{c}}). \end{aligned}$$

#### Proof

As in the proof of Proposition 4.5 we can exploit that $\Psi _V^*$ is a sum of non-negative terms over $r=1,\ldots , R$. Hence, it is sufficient to show the desired limsup estimate for each reaction individually. For notational simplicity we drop the reaction index *r*.

Defining $\varrho ^V=\rho ^V{\mathrm {d}}{\varvec{c}}=\iota _V({\varvec{u}}^V)$, relation (4.5b) leads us to the integral representation

$$\begin{aligned} \Psi _V^*({\varvec{u}}^V,{\varvec{\xi }}^V) = \frac{\kappa _*}{2}\int _{{\varvec{c}}\in {\varvec{C}}} \Lambda \big ( \,\rho ^{V,a}({\varvec{c}}) \,, \rho ^{V,b}({\varvec{c}}) \big ) M_V^\xi ({\varvec{c}}) \;\!\mathrm {d}{\varvec{c}}, \end{aligned}$$

where

$$\begin{aligned} \rho ^{V,a}({\varvec{c}}) = a_V({\varvec{c}}) \rho ^V({\varvec{c}}{+}\tfrac{1}{V}{\varvec{\alpha }}), \quad \rho ^{V,b}({\varvec{c}})= b_V({\varvec{c}}) \rho ^V({\varvec{c}}{+}\tfrac{1}{V}{\varvec{\beta }}), \end{aligned}$$

and the functions $a_V$, $b_V$, and $M_V^\xi$ are given

$$\begin{aligned}&a_V({\varvec{c}}) = \frac{\!\;{\mathbb {B}}_{V}^{{\varvec{\alpha }}}\!({\varvec{n}})\!\; }{V{\varvec{c}}_*^{\varvec{\alpha }}} ,\quad b_V({\varvec{c}}) = \frac{\!\;{\mathbb {B}}_{V}^{{\varvec{\beta }}}\!({\varvec{n}})\!\;}{V{\varvec{c}}_*^{\varvec{\beta }}}, \quad M_V^\xi ({\varvec{c}}) = V^2 \big ({\varvec{\xi }}^V_{{\varvec{n}}+ {\varvec{\alpha }}} {-}{\varvec{\xi }}^V_{{\varvec{n}}+ {\varvec{\beta }}} \big )^2 \quad \text {for } {\varvec{c}}\in A^V_{\varvec{n}}. \end{aligned}$$

Using $\xi \in {\mathrm {C}}_{\mathrm {c}}^1({\varvec{C}})$ there exists $R>0$ such that $\mathop {\mathrm {sppt}}M_V^\xi \subset {\varvec{C}}_R:=B_R(0)\cap {\varvec{C}}$, and we have uniform convergence

$$\begin{aligned} \Vert a_V{-} a_\infty \Vert _{\mathrm{L^\infty ({\varvec{C}}_R)}} + \Vert b_V{-} b_\infty \Vert _{\mathrm{L^\infty ({\varvec{C}}_R)}} + \Vert M_V^\xi - (\nabla \xi \cdot {\varvec{\gamma }})^2\Vert _{\mathrm{L^\infty ({\varvec{C}}_R)}} \rightarrow 0 \ \text { as }V\rightarrow \infty , \end{aligned}$$

where $a_\infty ({\varvec{c}})={\varvec{c}}^{\varvec{\alpha }}/{\varvec{c}}_*^{\varvec{\alpha }}$, $b_\infty ({\varvec{c}})={\varvec{c}}^{\varvec{\beta }}/{\varvec{c}}_*^{\varvec{\beta }}$, and ${\varvec{\gamma }}= {\varvec{\alpha }}{-}{\varvec{\beta }}$. Using $\Lambda (r,t)\le \frac{1}{2}(r{+}t)$, the uniform boundedness of $a_V$ and $b_V$ on ${\varvec{C}}_R$, and that $\varrho _V$ is a probability measure, we see that in the limsup of $\Psi _V^*({\varvec{u}}^V,{\varvec{\xi }}^V)$ we can replace $M_V^\xi$ by $\big (({\varvec{\alpha }}{-}{\varvec{\beta }})\cdot \nabla \xi \big )^2$ without changing the limsup in the left-hand side of (4.25).

Next we consider the functionals $F : {\mathscr {M}}({\varvec{C}}_R) \times {\mathscr {M}}({\varvec{C}}_R) \rightarrow [0, + \infty ]$ given by

$$\begin{aligned} F(\varrho _1,\varrho _2)&= \int _{{\varvec{C}}_R} f(\rho _1({\varvec{c}}), \rho _2({\varvec{c}})) \big ({\varvec{\gamma }}\cdot \nabla \xi ({\varvec{c}})\big )^2 \;\!\mathrm {d}{\varvec{c}}\ \text { with } f(r,t) \\&= \left\{ \begin{array}{cl} r{+}t{-}\Lambda (r,t), &{} \text {for }r,t \ge 0,\\ + \infty , &{} \text {else}.\end{array} \right. \end{aligned}$$

Note that $f(r,t) \ge \frac{1}{2}(r{+}t)$. Moreover, *f* is convex and positively homogeneous of degree 1. Thus, *F* is weak* lower semi-continuous on ${\mathscr {M}}({\varvec{C}}_R){\times }{\mathscr {M}}({\varvec{C}}_R)$, cf. [20, Thm. 6.57]. Now using the convergences

$$\begin{aligned} \varrho ^{V,a} \overset{*}{\rightharpoonup }a_\infty \varrho \big |_{{\varvec{C}}_R} \quad \text { and } \quad \varrho ^{V,b} \overset{*}{\rightharpoonup }b_\infty \varrho \big |_{{\varvec{C}}_R} \quad \text {as } V\rightarrow \infty , \end{aligned}$$

we obtain the liminf estimate $\liminf _{V\rightarrow \infty } F(\varrho ^{V,a},\varrho ^{V,b}) \ge F(a_\infty \varrho ,b_\infty \varrho )$.

Thus, in the view of the identity

$$\begin{aligned}&\int _{{\varvec{C}}_R} \Lambda (\rho ^{V,a},\rho ^{V,b}) ({\varvec{\gamma }}\cdot \nabla \xi ({\varvec{c}}))^2 \;\!\mathrm {d}{\varvec{c}}= \int _{{\varvec{C}}_R} (\rho ^{V,a}{+} \rho ^{V,b}) \big ({\varvec{\gamma }}\cdot \nabla \xi ({\varvec{c}})\big )^2 \;\!\mathrm {d}{\varvec{c}}- F(\varrho ^{V,a},\varrho ^{V,b}), \end{aligned}$$

and observing that the first term on the right-hand side is weak$^*$ continuous, the limsup for $V\rightarrow \infty$ gives

$$\begin{aligned} \limsup _{V\rightarrow \infty } \Psi _V^*({\varvec{u}}^V,{\varvec{\xi }}^V)&= \frac{\kappa _*}{2} \limsup _{V \rightarrow \infty } \int _{{\varvec{C}}_R} \Lambda (\rho ^{V,a},\rho ^{V,b}) ({\varvec{\gamma }}\cdot \nabla \xi ({\varvec{c}}))^2 \;\!\mathrm {d}{\varvec{c}}\\&\le \frac{\kappa _*}{2} \int _{{\varvec{C}}_R} (a_\infty {+}b_\infty ) \big ({\varvec{\gamma }}\cdot \nabla \xi )^2 \;\!\mathrm {d}\varrho - \frac{\kappa _*}{2} F(a_\infty \varrho ,b_\infty \varrho ) \\&= \frac{\kappa _*}{2} \int _{{\varvec{C}}_R} \Lambda (a_\infty ,b_\infty ) \big ({\varvec{\gamma }}{\cdot } \nabla \xi ({\varvec{c}})\big )^2 \;\!\mathrm {d}\varrho ({\varvec{c}}) =\Psi _\mathrm {Lio}^*(\varrho ,\xi ). \end{aligned}$$

This is the desired result for one reaction, and the full result follows by summation over $r=1,\ldots ,R$ and the definition of ${\mathbb {K}}$, namely $\nabla \xi \cdot {\mathbb {K}}\nabla \xi = \sum _{r=1}^R \kappa _*^r \Lambda \big ( \frac{{\varvec{c}}^{{\varvec{\alpha }}^r}}{{\varvec{c}}_*^{{\varvec{\alpha }}^r}}, \frac{{\varvec{c}}^{{\varvec{\beta }}^r}}{{\varvec{c}}_*^{{\varvec{\beta }}^r}} \big )({\varvec{\gamma }}^r{\cdot } \nabla \xi )^2$. $\square$

### Convergence of Solutions

Here we provide the general convergence result as $V\rightarrow \infty$ for the appropriately embedded solutions ${\varvec{u}}^V:{[0,\infty [} \rightarrow {\mathscr {P}}({\mathcal {N}})$ of the CME to the solutions $\varrho :{[0,\infty [} \rightarrow {\mathscr {P}}({\varvec{C}})$ of the Liouville equation, which is a simple transport along the solutions of the RRE ${\dot{{\varvec{c}}}} = -{\varvec{R}}({\varvec{c}}) = -{\mathbb {K}}({\varvec{c}}) {\mathrm {D}}E({\varvec{c}})$. Our approach follows the strategy of evolutionary $\Gamma$-convergence as initiated in [54, 58] with the new idea of dualization as introduced in [35].

#### Theorem 4.7

(Evolutionary $\Gamma$-convergence of CME to Liouville) For all $V>1$ consider a solution ${\varvec{u}}^V:{[0,\infty [} \rightarrow {\mathscr {P}}({\mathcal {N}})$ of the CME (3.2). Assume that the initial conditions are well-prepared in the sense that

$$\begin{aligned} \iota _V({\varvec{u}}^V(0)) \overset{*}{\rightharpoonup }\varrho ^0 \text { in }{\mathscr {P}}({\varvec{C}}) \quad \text {and} \quad {\mathcal {E}}_V({\varvec{u}}^V(0)) \rightarrow {\varvec{E}}(\varrho ^0). \end{aligned}$$

Then, for all $t > 0$, we have the convergence

$$\begin{aligned} \iota _V({\varvec{u}}^V(t)) \overset{*}{\rightharpoonup }\varrho (t) \text { in }{\mathscr {P}}({\varvec{C}}) \quad \text {and} \quad {\mathcal {E}}_V({\varvec{u}}^V(t)) \rightarrow {\varvec{E}}(\varrho (t)), \end{aligned}$$

where $\varrho :{[0,\infty [} \rightarrow {\mathscr {P}}({\varvec{C}})$ is the unique solution of the Liouville equation (4.3) starting at $\varrho (0)=\varrho ^0$, i.e., for all $\varphi \in {\mathrm {C}}^1_{\mathrm {c}}([0,T]{\times }{\varvec{C}})$ with $\varphi (T,\cdot ) = 0$ we have

4.26 $$\begin{aligned} \int _{\varvec{C}}\varphi (0,{\varvec{c}}) \varrho ^0({\mathrm {d}}{\varvec{c}}) + \int _0^T\int _{\varvec{C}}\Big (\partial _t \varphi (t,{\varvec{c}}) - \nabla \varphi (t,{\varvec{c}}){\cdot } {\mathbb {K}}({\varvec{c}}) \nabla E({\varvec{c}}) \Big ) \varrho (t,{\mathrm {d}}{\varvec{c}}) \;\!\mathrm {d}t =0. \end{aligned}$$

Moreover, for all $r,s\in [0,T]$ with $r<s$ we have the energy identity

4.27 $$\begin{aligned} {\varvec{E}}(\varrho (s)) + 2 \int _r^s \Psi _\mathrm {Lio}^*\big (\varrho (t), - {\mathrm {D}}{\varvec{E}}(\varrho (t))\big ) \;\!\mathrm {d}t = {\varvec{E}}(\varrho (r)). \end{aligned}$$

For the proof we use the energy-dissipation principle for $V \ge 1$ and pass to the limit in each of the terms. If ${\varvec{u}}^V$ is a solution of the CME, then for all $T>0$ we have

4.28 $$\begin{aligned} {\mathcal {E}}_V({\varvec{u}}^V(T)) + \int _0^T \Psi _V({\varvec{u}}^V,{\dot{{\varvec{u}}}}^V) + \Psi _V^*\big ({\varvec{u}}^V,{-}{\mathrm {D}}{\mathcal {E}}_V({\varvec{u}}^V)\big ) \;\!\mathrm {d}t = {\mathcal {E}}_V({\varvec{u}}^V(0)). \end{aligned}$$

Following the ideas in [11] for the passage from a Markov chain to the Fokker–Planck equation or the general methods in evolutionary $\Gamma$-convergence, we want to pass to the limit in each of the four terms. As a general fact, it will be sufficient to obtain liminf estimates on the left-hand side, since by a chain-rule argument an estimate with “$\le$” instead of equality can be turned back into an equality. Moreover, by the assumptions of the theorem we see that the right-hand side converges to the desired limit.

However, it is rather delicate to pass to the limit in the integral $\int _0^T \Psi _V({\varvec{u}}^V,{\dot{{\varvec{u}}}}^V) \;\!\mathrm {d}t$, because the potential $\Psi _V$ is only implicitly defined and we expect the limit to be given in terms of the Benamou-Brenier formula for the Wasserstein distance induced by the metric on $({\varvec{C}},{\mathbb {K}})$. A major difficulty is even to obtain a suitable equi-continuity for the solutions ${\varvec{u}}^V$ to be able to extract a subsequence converging at all times. In particular, it is unclear how to pass to the limit in $\iota _V({\dot{{\varvec{u}}}}^V(t))$ by a direct argument.

Hence, following [35], we estimate the primal dissipation potential $\Psi _V$ from below using the definition in terms of the Legendre transform of $\Psi _V^*$. Using additionally an integration by parts we have

$$\begin{aligned}&\int _0^T \Psi _V({\varvec{u}}^V,{\dot{{\varvec{u}}}}^V)\;\!\mathrm {d}t \ge {\mathfrak {J}}_V ({\varvec{u}}^V,{\varvec{\eta }}) \quad \text { for all } {\varvec{\eta }}\in {\mathrm {C}}^1([0,T];\ell ^\infty ({\mathcal {N}})) \text { with}\\&{\mathfrak {J}}_V({\varvec{u}},{\varvec{\eta }}):= \langle {\varvec{u}}(T), {\varvec{\eta }}(T)\rangle - \langle {\varvec{u}}(0), {\varvec{\eta }}(0)\rangle - \int _0^T \langle {\varvec{u}}(t),{\dot{{\varvec{\eta }}}}(t) \rangle + \Psi _V^*({\varvec{u}}(t),{\varvec{\eta }}(t)) \;\!\mathrm {d}t, \end{aligned}$$

where $\langle {\varvec{u}},{\varvec{\eta }}\rangle := \sum _{n\in {\mathcal {N}}} u_{\varvec{n}}\eta _{\varvec{n}}$. With this argument we can replace the energy-dissipation principle (4.28) by the estimate

4.29 $$\begin{aligned} {\mathcal {E}}_V({\varvec{u}}^V(T)) + \mathfrak J_V({\varvec{u}}^V, {\varvec{\eta }}) + \int _0^T \Psi _V^*\big ({\varvec{u}}^V, {-}{\mathrm {D}}{\mathcal {E}}_V({\varvec{u}}^V)\big ) \;\!\mathrm {d}t \le {\mathcal {E}}_V({\varvec{u}}^V(0)), \end{aligned}$$

which holds for all differentiable ${\varvec{\eta }}$. In this equation we are then able to pass to the limit $V\rightarrow \infty$, when choosing ${\varvec{\eta }}= {\varvec{\eta }}^V = \iota ^*_V(\xi )$ for a smooth function $\xi$.

At the end we are then able to calculate the supremum over all $\xi$ by using the especially simple quadratic structure in $\xi$, which mirrors the fact that the Liouville equation is a simple transport equation.

#### Proof of Theorem 4.7

**Step 1: Embedding and uniform a priori bounds** We now consider the family ${\varvec{u}}^V:[0,T] \rightarrow {\mathscr {P}}({\mathcal {N}})$ and embed it into ${\mathscr {P}}({\varvec{C}})$ via $\iota _V$ from (4.6). As in [11] we show an equi-continuity in a 1-Wasserstein distance, but introduce an additional weight accounting for our unbounded domain ${\varvec{C}}$. We define the maximal order *p* of all reactions via

$$\begin{aligned} p:= \max \{\, |{\varvec{\alpha }}^r|_1,|{\varvec{\beta }}^r|_1 \, | \, r=1,\ldots , R \,\} . \end{aligned}$$

For $\mu \in {\mathscr {M}}({\varvec{C}})$ and for $\varrho _0, \varrho _1 \in {\mathscr {P}}({\varvec{C}})$ we set

$$\begin{aligned} \Vert \mu \Vert _{1{\mathrm {W}}} := \sup \bigg \{\, \int _{\varvec{C}}f({\varvec{c}}) \;\!\mathrm {d}\mu ({\varvec{c}}) \; \bigg | \; f \in {\mathbb {F}} \,\bigg \} \quad \text {and} \quad d_{1{\mathrm {W}}}(\varrho _0,\varrho _1) = \Vert \varrho _0 - \varrho _1 \Vert _{1{\mathrm {W}}}, \end{aligned}$$

where $${\mathbb {F}}:= \{\, f\in {\mathrm {C}}^1({\varvec{C}}) \, | \, \sup _{\varvec{C}}(1{+}|{\varvec{c}}|^p) |\nabla f({\varvec{c}})| \le 1 \,\} $$.

Using the definition of the Markov generators ${\mathcal {Q}}^r_V$ in terms of the coefficients $\;{\mathbb {B}}_{V}^{{\varvec{\delta }}^r}\!({\varvec{n}})\!\;$, see (3.3), it is easy to derive the uniform estimate $\Vert \iota _V({\dot{{\varvec{u}}}}^V(t))\Vert _{1{\mathrm {W}}}\le C_{1{\mathrm {W}}}$ independently of the initial conditions and $V\ge 1$ (one simply needs $\sum u^V_{\varvec{n}}\equiv 1$). Hence, we obtain the uniform Lipschitz bound

$$\begin{aligned} d_{1{\mathrm {W}}}\big (\iota _V({\varvec{u}}^V(t)),\iota _V({\varvec{u}}^V(s))\big )\le C_{1{\mathrm {W}}}|t{-}s|\quad \text { for all }s,t\in [0,T] \text { and all }V\ge 1. \end{aligned}$$

Moreover, as ${\mathcal {E}}_V({\varvec{u}}^V(t)) \le {\mathcal {E}}_V({\varvec{u}}^V(0)) \le {\varvec{E}}(\varrho ^0)+o(1)_{V\rightarrow \infty }$ by well-preparedness, the equi-coercivity of ${\mathcal {E}}_V$ established in (4.18) yields the uniform bound

4.30 $$\begin{aligned} \exists \, V_* \ge 1, C_{\mathrm {B}}< \infty \ \forall \, t>0,\ V\ge V_*: \int _{\varvec{C}}(1{+}|{\varvec{c}}|) \iota _V({\varvec{u}}^V(t)) \;\!\mathrm {d}{\varvec{c}}\le C_{\mathrm {B}}. \end{aligned}$$

**Step 2: Extraction of a subsequence** The subset of ${\mathscr {P}}({\varvec{C}})$ defined by the boundedness of the above first moment is a compact subset of the metric space $({\mathscr {P}}({\varvec{C}}),d_{1{\mathrm {W}}})$. Indeed, using Prokhorov’s theorem one finds that this set is weak$^*$ sequentially compact. Since $d_{1{\mathrm {W}}}$ is dominated by the bounded Lipschitz metric (which metrizes weak$^*$ convergence), the compactness of $({\mathscr {P}}({\varvec{C}}),d_{1{\mathrm {W}}})$ follows.

Hence, we can apply the abstract Arzelà-Ascoli theorem in $({\mathscr {P}}({\varvec{C}}),d_{1{\mathrm {W}}})$ to extract a subsequence $V_k\rightarrow \infty$ and a limit function $\varrho :[0,T]\rightarrow {\mathscr {P}}({\varvec{C}})$ such that

4.31a $$\begin{aligned}&\forall \, t\in [0,T]:&\iota _V({\varvec{u}}^V(t)) \overset{*}{\rightharpoonup }\varrho (t) \text { in } {\mathscr {P}}({\varvec{C}}), \end{aligned}$$

4.31b $$\begin{aligned}&\forall \, s,t\in [0,T]:&d_{1{\mathrm {W}}}(\varrho (t),\varrho (s)) \le C_{1{\mathrm {W}}}|t{-}s|,\end{aligned}$$

4.31c $$\begin{aligned}&\forall \, t\in [0,T]:&{\varvec{E}}(\varrho (t)) \le {\varvec{E}}(\varrho ^0),\end{aligned}$$

4.31d $$\begin{aligned}&\text {the mapping } t \mapsto \varrho (t) \text { is weak* continuous}. \end{aligned}$$

At first, in place of (4.31a) one obtains $d_{1{\mathrm {W}}}\big (\iota _V({\varvec{u}}^V(t)),\varrho (t)\big )\rightarrow 0$. To derive (4.31a), we use the bound (4.30) together with the fact that any bounded continuous function can be uniformly approximated on compact sets by (multiples of) functions in ${\mathbb {F}}$. Similarly, (4.31d) follows from (4.31b). In particular, combining (4.31d) and the assumption $\iota _V({\varvec{u}}^V(0)) \overset{*}{\rightharpoonup }\varrho ^0$ we conclude $\varrho (0)=\varrho ^0$. Finally, (4.31c) follows via (4.31a) from Theorem 4.4:

$$\begin{aligned} {\varvec{E}}(\varrho (t)) \le \liminf _{V\rightarrow \infty } {\mathcal {E}}_V\big ({\varvec{u}}^V(t)\big ) \le \liminf _{V\rightarrow \infty } {\mathcal {E}}_V\big ({\varvec{u}}^V(0)\big ) = {\varvec{E}}(\varrho ^0). \end{aligned}$$

**Step 3: Limit passage in** (4.29) Combining (4.31a) for $t=T$ and Theorem 4.4 (cf. (4.19)), the first term satisfies the liminf estimate $\liminf _{V\rightarrow \infty } {\mathcal {E}}_V({\varvec{u}}^V(T)) \ge {\varvec{E}}(\varrho (T))$. For the last term we use the assumption ${\mathcal {E}}_V({\varvec{u}}^V(0)) \rightarrow {\varvec{E}}(\varrho ^0)={\varvec{E}}(\varrho (0))$.

For the third term we employ Proposition 4.5 for each $t\in [0,T]$ based on (4.31a). Using Fatou’s lemma we conclude the liminf estimate

$$\begin{aligned} \liminf _{V\rightarrow \infty } \int _0^T \Psi _V^*\big ({\varvec{u}}^V(t), {-}{\mathrm {D}}{\mathcal {E}}_V({\varvec{u}}^V(t))\big ) \;\!\mathrm {d}t&\ge \int _0^T \liminf _{V\rightarrow \infty }\Psi _V^*\big ({\varvec{u}}^V(t),{-}{\mathrm {D}}{\mathcal {E}}_V({\varvec{u}}^V(t))\big ) \;\!\mathrm {d}t \\&\ge \int _0^T \Psi ^*_\mathrm {Lio}\big (\varrho (t),{-}{\mathrm {D}}{\varvec{E}}(\varrho (t))\big )\;\!\mathrm {d}t. \end{aligned}$$

Thus, it remains to pass to the limit in $\mathfrak J_V({\varvec{u}}^V,\eta )$. For this we choose an arbitrary $\xi \in {\mathrm {C}}^1_{\mathrm {c}}([0,T]{\times }{\varvec{C}})$ and define ${\varvec{\xi }}^V(t)=\iota _V^*(\xi (t))$, cf. (4.7). With this choice we can apply Proposition for all $t\in [0,T]$ based on (4.31a). Now, Fatou’s lemma yields

$$\begin{aligned}&\liminf _{V\rightarrow \infty } {\mathfrak {J}}_V({\varvec{u}}^V ,{\varvec{\xi }}^V) \ge {\mathfrak {J}}_\mathrm {Lio}(\varrho ,\xi ) \quad \text {where} \\&{\mathfrak {J}}_\mathrm {Lio}(\varrho ,\xi ) := \int _{{\varvec{C}}} \xi (T,{\varvec{c}}) \varrho (T,{\mathrm {d}}{\varvec{c}}) - \int _{\varvec{C}}\xi (0,{\varvec{c}})\varrho (0,{\mathrm {d}}{\varvec{c}})\\&\quad - \int _0^T\int _{\varvec{C}}\Big ( \partial _t\xi (t,{\varvec{c}})+ \frac{1}{2}\nabla \xi (t,{\varvec{c}})\cdot {\mathbb {K}}({\varvec{c}})\nabla \xi (t,{\varvec{c}}) \Big ) \varrho (t,{\mathrm {d}}{\varvec{c}}) \;\!\mathrm {d}t. \end{aligned}$$

In summary, we conclude that the limit function $\varrho :[0,T]\rightarrow {\mathscr {P}}({\varvec{C}})$ satisfies

4.32 $$\begin{aligned} {\varvec{E}}(\varrho (T)) + {\mathfrak {J}}_\mathrm {Lio}(\varrho ,\xi ) + \int _0^T \Psi ^*_\mathrm {Lio}\big (\varrho (t),{-}{\mathrm {D}}{\varvec{E}}(\varrho (t))\big ) \;\!\mathrm {d}t \le {\varvec{E}}(\varrho (0)) \end{aligned}$$

for all $$\xi \in {\mathrm {C}}^1_{\mathrm {c}}([0,T]{\times }{\varvec{C}})$$.

**Step 4: Energy balance** By inserting $\xi \equiv 0$ in (4.32) we obtain the upper bound

$$\begin{aligned} {\mathcal {D}}(\varrho ;0,T):= \int _0^T\int _{\varvec{C}}\nabla E({\varvec{c}}){\cdot } {\mathbb {K}}({\varvec{c}}) \nabla E ({\varvec{c}}) \varrho (t,{\mathrm {d}}{\varvec{c}}) \;\!\mathrm {d}t \le 2\big ( {\varvec{E}}(\varrho (0)) - {\varvec{E}}(\varrho (T))\big ) . \end{aligned}$$

We want to show energy balance, i.e., equality when the factor 2 is omitted. For this purpose, we observe that the measures $\varrho (t,\cdot )\in {\mathscr {P}}({\varvec{C}})$ decay at infinity such that (4.31c) holds. Hence, we may also use $\xi (t,{\varvec{c}})=\lambda E({\varvec{c}})$ as testfunctions in (4.32). Writing shortly $e(t):={\varvec{E}}(\varrho (t))$ we find ${\mathfrak {J}}_\mathrm {Lio}(\varrho ,\lambda E )= \lambda \big (e(T) - e(0)\big ) - \frac{\lambda ^2}{2} {\mathcal {D}}(\varrho ;0,T)$ and obtain

$$\begin{aligned} -\lambda \big (e(0)- e(T)\big ) - \frac{\lambda ^2}{2} {\mathcal {D}}(\varrho ;0,T)= & {} {\mathfrak {J}}_\mathrm {Lio}(\varrho ,\lambda E ) \le e(0)- e(T)\\&-\frac{1}{2}{\mathcal {D}}(\varrho ;0,T) \quad \text {for all } \lambda \in {\mathbb {R}}. \end{aligned}$$

Maximizing with respect to $\lambda$ leads to $(e(0){-} e(T))^2/{\mathcal {D}}\le 2(e(0){-} e(T)) - {\mathcal {D}}$ which implies $e(0){-} e(T)={\mathcal {D}}$, or more explicitly ${\mathcal {D}}(\varrho ;0,T)= {\varvec{E}}(\varrho (0)) - {\varvec{E}}(\varrho (T))$, which is the desired energy balance (4.27) for $r=0$ and $s=T$.

Moreover, we can repeat the calculation on [0, *s*] with $0< s < T$ instead of [0, *T*]. The full result (4.27) follows by subtracting the identity on [0, *r*] from that on [0, *s*].

**Step 5: Weak form of gradient flow equation** With Step 4 we rewrite (4.32) as

$$\begin{aligned} {\mathfrak {J}}_\mathrm {Lio}(\varrho ,\xi ) \le {\varvec{E}}(\varrho (0))- {\varvec{E}}(\varrho (T)) - \frac{1}{2}{\mathcal {D}}(\varrho ;0,T)=\frac{1}{2}{\mathcal {D}}(\varrho ;0,T), \end{aligned}$$

and know that the left-hand side is maximized by $\xi :(t,{\varvec{c}})\mapsto - E({\varvec{c}})$. Inserting the test functions $\xi (t,{\varvec{c}}) = \delta \varphi (t,{\varvec{c}}) - E({\varvec{c}})$ with small $\delta >0$ and $\varphi \in {\mathrm {C}}^1_{\mathrm {c}}([0,T]{\times }{\varvec{C}})$ with $\varphi (T,\cdot )=0$ we arrive, after some cancellations and after dividing by $\delta >0$, at

$$\begin{aligned} -\int _{\varvec{C}}\varphi (0,{\varvec{c}}) \varrho (0,\;\!\mathrm {d}{\varvec{c}})- \int _0^T \int _{\varvec{C}}\Big (\partial _t\varphi -\nabla \varphi \cdot {\mathbb {K}}\nabla \big ( E{-}\tfrac{\delta }{2}\varphi \big ) \Big )\varrho (t,{\mathrm {d}}{\varvec{c}}) \;\!\mathrm {d}t \le 0. \end{aligned}$$

Taking the limit $\delta \searrow 0$ and replacing $\varphi$ by $-\varphi$, we obtain the desired result (4.26).

With this, Theorem 4.7 is established. $\square$

## Approximation via Fokker–Planck Equations

In the above section we have seen that the Liouville equation is the proper limit of the CME for $V \rightarrow \infty$. However, for finite but large *V* it can still be advantageous to replace the discrete CME by a continuous PDE with *V* as a large parameter. In this range the stochastic modeling is done by the so-called *Langevin dynamics*, see [24, 34, 61], which is based on a stochastic perturbation of the reaction-rate equation (RRE), see (1.4). At the level of probability distributions the corresponding model is the associated Fokker–Planck equation (FPE). We will discuss two different gradient flow approximations: in the first we simply add a suitable “entropic term” to the driving functional, but keep the dissipation fixed (cf. Sect. 5.2), while in the second we expand ${\mathcal {E}}_V$ and ${\mathcal {K}}_V$ such that all terms of order 1/*V* are correct (cf. Sect. 5.3).

### Improved Approximation of the Relative Entropy

We interpret the sum in the definition of ${\mathcal {E}}_V$ as a Riemann sum and replace it by a corresponding integral. The main point of the improvement is that we keep the entropy term $\frac{1}{V} \sum u_{\varvec{n}}\log u_{\varvec{n}}$ in the definition of ${\mathcal {E}}_V({\varvec{u}})$, which is in contrast to the limit ${\varvec{E}}$ obtained in Theorem 4.4. Working with absolutely continuous probability measures $\varrho ({\mathrm {d}}{\varvec{c}}) = \rho ({\varvec{c}}) \;\!\mathrm {d}{\varvec{c}}$ with $\rho \in {\mathrm {L}}^1({\varvec{C}})$, we can define the *V*-dependent entropy by

5.1 $$\begin{aligned} {\varvec{E}}_V(\varrho ) = \frac{1}{V} \int _{\varvec{C}}\rho ({\varvec{c}}) \log \Big (\frac{\rho ({\varvec{c}})}{W_V({\varvec{c}})}\Big ) \;\!\mathrm {d}{\varvec{c}}, \end{aligned}$$

where the equilibrium density $W_V\in {\mathrm {L}}^1({\varvec{C}})$ has to be chosen suitably. A first simple approximation is $\widetilde{W}_V({\varvec{c}})=\frac{1}{\widetilde{Z}_V} \,{\mathrm {e}}^{-V E({\varvec{c}})}$ with $E({\varvec{c}})=\sum _{i=1}^I c^*_i\lambda _{\mathrm {B}}(c_i/c^*_i)$ as above and $\widetilde{Z}_V=\int _{\varvec{C}}{\mathrm {e}}^{-V E({\varvec{c}})} \;\!\mathrm {d}{\varvec{c}}$. However, a better and more refined $W_V$ is obtained using the next order of expansion in Stirling’s formula (4.10) as well. For this we use the approximation $k_n \approx n+1/6$, i.e., $\log (n!) = n\log n - n +\frac{1}{2} \log \big (2\pi (n{+}\frac{1}{6})\big ) + O(1/n^2)$ for $n\rightarrow \infty$. Hence, taking the limits $V, \,|{\varvec{n}}| \rightarrow \infty$ such that $\frac{{\varvec{n}}}{V} \rightarrow {\varvec{c}}$, we obtain

$$\begin{aligned} - \frac{1}{V} \log w_{\varvec{n}}^V \approx E({\varvec{c}}) + \frac{1}{V} G_V({\varvec{c}}) \end{aligned}$$

with the *V*-dependent correction $G_V({\varvec{c}}) := \frac{1}{2}\sum _{i=1}^I \log \big ( 2\pi (V c_i + \tfrac{1}{6}) \big )$ for *E*.

We now take a probability measure $\varrho = \rho {\mathrm {d}}{\varvec{c}}\in {\mathscr {P}}({\varvec{C}})$ and a discrete approximation ${\varvec{u}}\approx \varkappa _V (\varrho ) \in {\mathscr {P}}({\mathcal {N}})$, where $\varkappa _V : {\mathscr {P}}({\varvec{C}}) \rightarrow {\mathscr {P}}({\mathcal {N}})$ is the natural projection defined in (4.8). Then the Riemann-sum approximation results in

$$\begin{aligned} {\mathcal {E}}_V({\varvec{u}})&= \frac{1}{V}\sum _{{\varvec{n}}\in {\mathcal {N}}} u_{\varvec{n}}\log u_{\varvec{n}}- \frac{1}{V}\sum _{{\varvec{n}}\in {\mathcal {N}}} u_{\varvec{n}}\log w_{\varvec{n}}^V \\&\approx \frac{1}{V} \int _{{\varvec{C}}} \rho ({\varvec{c}}) \log \rho ({\varvec{c}}) \;\!\mathrm {d}{\varvec{c}}- I \frac{\log V}{V} + \int _{{\varvec{C}}}\Big ( E({\varvec{c}}) + \frac{1}{V} G_V({\varvec{c}})\Big ) \rho ({\varvec{c}}) \;\!\mathrm {d}{\varvec{c}}\\&= \frac{1}{V} \int _{\varvec{C}}\rho ({\varvec{c}}) \log \Big (\frac{\rho ({\varvec{c}})}{{\widehat{W}}(V,{\varvec{c}}, {\varvec{c}}^*)}\Big ) \;\!\mathrm {d}{\varvec{c}}, \\ \text {where }\,&{\widehat{W}}(V,{\varvec{c}}, {\varvec{c}}_*) = \prod _{i=1}^I {\widehat{{\mathsf {W}}}}(V,c_i,c_i^*) \ \text { with } {\widehat{{\mathsf {W}}}}(V,c,c^*) = \frac{V {\mathrm {e}}^{-V c^*\lambda _{\mathrm {B}}(c/c^*)}}{ \sqrt{2\pi (V c + 1/6) }}. \end{aligned}$$

The probability density $W_V$ is then defined by normalizing ${\widehat{W}}(V, \cdot , {\varvec{c}}_*)$. We thus set ${\mathsf {Z}}(V, c^*) := \int _0^\infty {\widehat{{\mathsf {W}}}}(V, c, c^*) \;\!\mathrm {d}c$ and

5.2 $$\begin{aligned} W_V ({\varvec{c}}) := \prod _{i=1}^I {\mathsf {W}}(V,c_i,c_i^*) \quad \text { with } {\mathsf {W}}(V,c,c^*) := \frac{{\widehat{{\mathsf {W}}}}(V,c,c^*)}{{\mathsf {Z}}(V, c^*)}. \end{aligned}$$

This yields the expansion

$$\begin{aligned} -\frac{1}{V} \log W_V({\varvec{c}}) = E({\varvec{c}}) + \frac{1}{V} E^V_1({\varvec{c}}) \quad \text {where} \quad E^V_1({\varvec{c}}) = \widehat{{\mathsf {z}}}(V,{\varvec{c}}_*) + \frac{1}{2}\sum _{i=1}^I \log \big (V c_i{+}\tfrac{1}{6} \big ) \end{aligned}$$

with $\widehat{{\mathsf {z}}}(V,{\varvec{c}}_*) := \sum _{i=1}^I \log \big (\sqrt{2\pi }\,{\mathsf {Z}}(V,c_i^*)/V\big )$. In summary, for ${\varvec{E}}_V$ defined via (5.1) and (5.2) we have

5.3 $$\begin{aligned} {\varvec{E}}_V(\varrho ) = {\varvec{E}}(\varrho )+ \frac{1}{V} \int _{\varvec{C}}\big (\rho \log \rho +E^V_1 \rho \big ) \;\!\mathrm {d}{\varvec{c}}, \end{aligned}$$

and $${\mathrm {D}}{\varvec{E}}_V(\varrho )({\varvec{c}})=\frac{1}{V} \log \rho ({\varvec{c}}) -\frac{1}{V} \log W_V({\varvec{c}})$$.

### Simple Fokker–Planck Approximation

Here we keep the *V*-independent Onsager operator ${\varvec{K}}(\varrho ):\xi \mapsto -{\mathrm {div}}\big (\varrho {\mathbb {K}}\nabla \xi \big )$ of the Liouville equation and obtain the *V*-dependent continuous gradient system $({\mathscr {P}}({\varvec{C}}), {\varvec{E}}_V,{\varvec{K}})$. The associated gradient-flow equation ${\dot{\varrho }} = - {\varvec{K}}(\varrho ){\mathrm {D}}{\varvec{E}}_V(\varrho )$ is the FPE

5.4 $$\begin{aligned} {\dot{\rho }} = {\mathrm {div}}\Big ( \frac{1}{V} {\mathbb {K}}({\varvec{c}}) \nabla \rho + \rho {\varvec{R}}({\varvec{c}})+ \rho {\varvec{A}}_V({\varvec{c}})\Big ), \end{aligned}$$

where we used ${\mathbb {K}}({\varvec{c}}) {\mathrm {D}}E({\varvec{c}})={\varvec{R}}({\varvec{c}})$ and set ${\varvec{A}}_V({\varvec{c}}):= \frac{1}{2}{\mathbb {K}}({\varvec{c}}) \big ( \frac{1}{Vc_i{+}1/6}\big )_{i=1,\ldots ,I}$.

We expect that this FPE is a good approximation to the CME for all sufficiently large *V*. In particular, (5.4) has the steady state $\rho = W_V$, which is close to the discrete steady state ${\varvec{w}}^V\in {\mathscr {P}}({\mathcal {N}})$ using the embedding as above. In contrast, the only steady states of the Liouville equation (4.1) are concentrated on the equilibria of ${\dot{{\varvec{c}}}}=-{\varvec{R}}({\varvec{c}})$. Of course, the FPE still respects the invariant sets ${\varvec{I}}({\varvec{q}})$, because the mobility ${\mathbb {K}}$ of the Onsager operator ${\varvec{K}}$ is the same as for the Liouville equation. In particular, $\rho = W_V$ is the unique equilibrium density if and only if ${\mathbb {K}}$ has full rank, i.e., ${\varvec{I}}({\varvec{q}})={\varvec{C}}$ for all ${\varvec{q}}\in {\mathfrak {Q}}$.

The simpler choice $\widetilde{W}_V({\varvec{c}})=\frac{1}{\widetilde{Z}(V)} {\mathrm {e}}^{-V E({\varvec{c}})}$ for the equilibrium yields the relative entropy

$$\begin{aligned} \widetilde{\varvec{E}}_V (\varrho ) = \frac{1}{V} \int _{\varvec{C}}\rho ({\varvec{c}}) \log \Big (\frac{\rho ({\varvec{c}})}{\widetilde{W}_V({\varvec{c}})}\Big ) \;\!\mathrm {d}{\varvec{c}}= \int _{\varvec{C}} \Big (\frac{1}{V} \log \rho ({\varvec{c}}) {+} E({\varvec{c}}) \Big ) \rho ({\varvec{c}}) \;\!\mathrm {d}{\varvec{c}}+ \frac{\log (\widetilde{Z}(V))}{V}. \end{aligned}$$

The flow equation ${\dot{\varrho }} = - {\varvec{K}}(\varrho ){\mathrm {D}}\widetilde{\varvec{E}}_V(\varrho )$ induced by the gradient system $({\mathscr {P}}({\varvec{C}}),\widetilde{\varvec{E}}_V,{\varvec{K}})$ is the simplified FPE

5.5 $$\begin{aligned} {\dot{\rho }} = {\mathrm {div}}\Big ( \frac{1}{V} {\mathbb {K}}({\varvec{c}}) \nabla \rho + \rho {\varvec{R}}({\varvec{c}}) \Big ), \end{aligned}$$

which is the same as (5.4) but with ${\varvec{A}}_V\equiv 0$. The simplified equation will be used below as well, since $\widetilde{W}_V$ has a simpler explicit form.

We believe that this approximation is suitable for many purposes. However, it does not produce the correct diffusion as derived in [34, Eq.  (1.7)]. This diffusion correction is used to improve the RRE ${\dot{{\varvec{c}}}} = - {\varvec{R}}({\varvec{c}})$ by replacing it by a stochastic differential equation called the chemical Langevin equations (CLE) in [24, 61], see (1.4). The associated Fokker–Planck equation takes the form

5.6 $$\begin{aligned} {\dot{\rho }} = \frac{1}{V} \sum _{i,j=1}^I \partial _{ij}^2 \big ( \rho \widehat{\mathbb {K}}_{\mathrm {CLE}}({\varvec{c}})_{ij} \big ) + {\mathrm {div}}\big ( \rho {\varvec{R}}({\varvec{c}})\big ) , \end{aligned}$$

where $\widehat{\mathbb {K}}_\mathrm {CLE}({\varvec{c}}) \in {\mathbb {R}}^{I{\times }I}$ is given in (1.6) and differs from ${\mathbb {K}}$ as the logarithmic mean $\Lambda (a,b)$ between $a={\varvec{c}}^{{\varvec{\alpha }}^r}/{\varvec{c}}^{{\varvec{\alpha }}^r}_*$ and $b={\varvec{c}}^{{\varvec{\beta }}^r}/{\varvec{c}}^{{\varvec{\beta }}^r}_*$ is replaced by the arithmetic mean $\frac{1}{2}(a{+}b)$. Obviously, (5.6) does not have a gradient structure with respect to $\widehat{\mathbb {K}}_\mathrm {CLE}$, because there is no function ${\varvec{c}}\mapsto \widehat{E}({\varvec{c}})$ such that ${\varvec{R}}({\varvec{c}})=\widehat{\mathbb {K}}_\mathrm {CLE}({\varvec{c}})\nabla \widehat{E}({\varvec{c}})$.

### Fokker–Planck Equation with Higher-Order Terms

To derive a proper expansion for the term of order 1/*V* in the evolution equation, we work with the *V*-dependent entropy ${\varvec{E}}_V$ defined in Sect. 5.1. Up to an irrelevant *V*-dependent constant, this functional approximates ${\mathcal {E}}_V$ from (3.7) up to order $1/V^2$.

Similarly, we need to derive a suitable expansion for the dissipation potential, which can be done for each reaction independently. The discrete dual dissipation potential is given by (4.5b), namely

$$\begin{aligned} \Psi _V^*({\varvec{u}},{\varvec{\xi }})= \frac{V}{2} \sum _{{\varvec{n}}\in {\mathcal {N}}} \Lambda \big ( k_{\mathrm {fw}}\!\;{\mathbb {B}}_{V}^{{\varvec{\alpha }}}\!({\varvec{n}})\!\; u_{{\varvec{n}}+{\varvec{\alpha }}}, k_{\mathrm {bw}}\!\;{\mathbb {B}}_{V}^{{\varvec{\beta }}}\!({\varvec{n}})\!\; u_{{\varvec{n}}+{\varvec{\beta }}} \big ) \big ( \mu _{{\varvec{n}}{+}{\varvec{\alpha }}}{-} \mu _{{\varvec{n}}+{\varvec{\beta }}}\big )^2. \end{aligned}$$

For a smooth function $\xi : {\varvec{C}}\rightarrow {\mathbb {R}}$ we use the second-order accurate midpoint approximation ${\varvec{\mu }}= \widehat{\varvec{\mu }}^\xi _V: {\varvec{n}}\mapsto \xi \big (\frac{1}{V}({\varvec{n}}{+}{\varvec{\delta }})\big )$ with ${\varvec{\delta }}= \frac{1}{2}(1,\ldots ,1)$ to obtain the expansion

$$\begin{aligned} V\big ( \mu _{{\varvec{n}}+{\varvec{\alpha }}} - \mu _{{\varvec{n}}+{\varvec{\beta }}}\big ) = \nabla \xi ({\mathsf {c}}_{\varvec{n}}^V)\cdot \big ({\varvec{\alpha }}{-}{\varvec{\beta }}) + O(1/V^2)_{V \rightarrow \infty } \ \text { with } {\mathsf {c}}^V_{\varvec{n}}:=\frac{1}{V} \Big ({\varvec{n}}{+}\frac{{\varvec{\alpha }}{+}{\varvec{\beta }}}{2}{+}{\varvec{\delta }}\Big ), \end{aligned}$$

where we used symmetric difference quotients to obtain second order accuracy. Moreover, for a smooth and sufficiently fast decaying $\varrho =\rho {\mathrm {d}}{\varvec{c}}\in {\mathscr {P}}({\varvec{C}})$ we define the associated discrete ${\varvec{u}}\in {\mathscr {P}}({\mathcal {N}})$ via ${\varvec{u}}= \varkappa _V(\varrho ) = \iota ^*_V\varrho$, which yields $V^Iu_{\varvec{n}}=\rho \big (\frac{1}{V}({\varvec{n}}{+}{\varvec{\delta }})\big ) + O(1/V^2)$,

$$\begin{aligned}&V^I u_{{\varvec{n}}+{\varvec{\alpha }}} = \rho ({\mathsf {c}}^V_{\varvec{n}}) + \frac{1}{2V} \nabla \rho ({\mathsf {c}}^V_{\varvec{n}}) \cdot ( {\varvec{\alpha }}{-}{\varvec{\beta }})+ O(1/V^2), \end{aligned}$$

and similarly for $V^I u_{{\varvec{n}}+{\varvec{\beta }}}$. Hence, for the arguments of $\Lambda$ we find the expansion

$$\begin{aligned}&\frac{1}{V} \!\;{\mathbb {B}}_{V}^{{\varvec{\alpha }}}\!({\varvec{n}})\!\; V^{I} u_{{\varvec{n}}+{\varvec{\alpha }}} = ({\mathsf {c}}^V_{\varvec{n}})^{\varvec{\alpha }}\rho ({\mathsf {c}}^V_{\varvec{n}}) + \frac{1}{V} F^V_{\varvec{n}}+ O(1/V^2) \\&\quad \text { with } F^V_{\varvec{n}}= - ({\mathsf {c}}_{\varvec{n}}^V)^{\varvec{\alpha }}\rho ({\mathsf {c}}_{\varvec{n}}^V) \sum _{i=1}^I \frac{\alpha _i\beta _i}{2({\mathsf {c}}_{\varvec{n}}^V)_i} + \frac{1}{2} ({\mathsf {c}}_{\varvec{n}}^V)^{\varvec{\alpha }}\nabla \rho ({\mathsf {c}}_{\varvec{n}}^V)\cdot ({\varvec{\alpha }}{-}{\varvec{\beta }}). \end{aligned}$$

For all smooth functions $f, g : {\varvec{C}}\rightarrow {\mathbb {R}}$ with compact support in int$({\varvec{C}})$, the trapezoidal rule for Riemann integrals gives

$$\begin{aligned} \sum _{{\varvec{n}}\in {\mathcal {N}}} \Big (f({\mathsf {c}}^V_{\varvec{n}}) + \frac{1}{V} g({\mathsf {c}}^V_{\varvec{n}}) \Big ) \frac{1}{V^I} = \int _{\varvec{C}}\Big (f({\varvec{c}}) + \frac{1}{V} g({\varvec{c}}) \Big ) \;\!\mathrm {d}{\varvec{c}}+ O(1/V^2). \end{aligned}$$

Hence, for smooth $\rho$ and $\xi$ we find the expansion

$$\begin{aligned}&\Psi _V^{*}\big (\varkappa _V (\varrho ),\widehat{\varvec{\mu }}^\xi _V\big ) = \Phi ^*_V(\varrho ,\xi ) + O(1/V^2) \text { for }V\rightarrow \infty \text { with}\\&\Phi _V^{*}(\varrho ,\xi ) = \frac{1}{2} \int _{\varvec{C}}\Big ( \Lambda (k_{\mathrm {fw}}{\varvec{c}}^{\varvec{\alpha }}, k_{\mathrm {bw}}{\varvec{c}}^{\varvec{\beta }}) \rho ({\varvec{c}}) + \frac{1}{V} \Upsilon \big ({\varvec{c}},\rho ({\varvec{c}}),\nabla \rho ({\varvec{c}})\big )\Big ) \big (\nabla \xi ({\varvec{c}})\cdot ({\varvec{\alpha }}{-}{\varvec{\beta }})\big )^2 \;\!\mathrm {d}{\varvec{c}}, \end{aligned}$$

where the correction term $\Upsilon$ takes the explicit form

$$\begin{aligned}&\Upsilon ({\varvec{c}},\rho ,{\varvec{p}}) = \Upsilon _0({\varvec{c}}) \rho + \Upsilon _1 ({\varvec{c}})\,{\varvec{p}}\varvec{\cdot }({\varvec{\alpha }}{-}{\varvec{\beta }}) \quad \text {with}\\&\Upsilon _0({\varvec{c}}) = - \frac{1}{2} \Lambda (k_{\mathrm {fw}}{\varvec{c}}^{\varvec{\alpha }}, k_{\mathrm {bw}}{\varvec{c}}^{\varvec{\beta }}) \, {\varvec{\alpha }}{\varvec{\cdot }} {\check{{\varvec{C}}}} {\varvec{\beta }}\ \text { with } {\check{{\varvec{C}}}} = \mathrm {diag}(c_i^{-1})_{i=1,\ldots ,I}, \\&\Upsilon _1({\varvec{c}}) = \Lambda (k_{\mathrm {fw}}{\varvec{c}}^{\varvec{\alpha }}, k_{\mathrm {bw}}{\varvec{c}}^{\varvec{\beta }}) \, \frac{ k_{\mathrm {fw}}{\varvec{c}}^{\varvec{\alpha }}{+} k_{\mathrm {bw}}{\varvec{c}}^{\varvec{\beta }}{-} 2\Lambda (k_{\mathrm {fw}}{\varvec{c}}^{\varvec{\alpha }}, k_{\mathrm {bw}}{\varvec{c}}^{\varvec{\beta }}) }{2(k_{\mathrm {fw}}{\varvec{c}}^{\varvec{\alpha }}{-} k_{\mathrm {bw}}{\varvec{c}}^{\varvec{\beta }}) }. \end{aligned}$$

Here we used the relation $\partial _a \Lambda (a,b)= \frac{\Lambda (a,b)}{a}\, \frac{a- \Lambda (a,b)}{a-b}$, giving

$$\begin{aligned}&a\partial _a \Lambda (a,b) {+} b\partial _b \Lambda (a,b)=\Lambda (a,b) \ \text { and } \ \\&\quad a\partial _a \Lambda (a,b) {-} b\partial _b \Lambda (a,b)=\Lambda (a,b) \frac{a {+} b{-} 2\Lambda (a,b)}{a-b} . \end{aligned}$$

Now we are in the position to calculate the first-order correction to the Liouville equation from the approximate entropy ${\varvec{E}}_V$ (cf. (5.3)) and the dual dissipation potential $\Psi ^{*}_V$, namely ${\dot{\varrho }} ={\mathrm {D}}_\xi \Phi ^{*}_V\big (\varrho , {-}{\mathrm {D}}{\varvec{E}}_V(\varrho ) \big )$, which yields

$$\begin{aligned} {\dot{\rho }}&= {\mathrm {div}}\left\{ \Big (\rho \,\widehat{a}({\varvec{c}}) + \frac{1}{V}\big [ \rho \, \widehat{b}_0({\varvec{c}}) + \widehat{b}_1({\varvec{c}}) \nabla \rho \cdot ({\varvec{\alpha }}{-} {\varvec{\beta }}) \big ] + O(1/V^2)\Big ) \big ({\varvec{\alpha }}{-}{\varvec{\beta }}\big ) \right\} , \end{aligned}$$

where the coefficients are given by

$$\begin{aligned}&\widehat{a}({\varvec{c}})= \Lambda (k_{\mathrm {fw}}{\varvec{c}}^{\varvec{\alpha }}, k_{\mathrm {bw}}{\varvec{c}}^{\varvec{\beta }}) \,({\varvec{\alpha }}{-}{\varvec{\beta }})\varvec{\cdot }\nabla E({\varvec{c}})\ = \ k_{\mathrm {fw}}{\varvec{c}}^{\varvec{\alpha }}{-} k_{\mathrm {bw}}{\varvec{c}}^{\varvec{\beta }}, \\&\widehat{b}_0({\varvec{c}})=\Lambda (k_{\mathrm {fw}}{\varvec{c}}^{\varvec{\alpha }}, k_{\mathrm {bw}}{\varvec{c}}^{\varvec{\beta }})\, ({\varvec{\alpha }}{-}{\varvec{\beta }})\varvec{\cdot }{\check{{\varvec{C}}}} {\varvec{\delta }}- \frac{1}{2} (k_{\mathrm {fw}}{\varvec{c}}^{\varvec{\alpha }}{-} k_{\mathrm {bw}}{\varvec{c}}^{\varvec{\beta }}) \,{\varvec{\alpha }}\varvec{\cdot }{\check{{\varvec{C}}}} {\varvec{\beta }}, \\&\widehat{b}_1({\varvec{c}})= \Lambda (k_{\mathrm {fw}}{\varvec{c}}^{\varvec{\alpha }}, k_{\mathrm {bw}}{\varvec{c}}^{\varvec{\beta }}) + \Upsilon _1({\varvec{c}}) ({\varvec{\alpha }}{-}{\varvec{\beta }}){\varvec{\cdot }}\nabla E({\varvec{c}}) \ = \ \frac{1}{2}(k_{\mathrm {fw}}{\varvec{c}}^{\varvec{\alpha }}{+}k_{\mathrm {bw}}{\varvec{c}}^{\varvec{\beta }}) . \end{aligned}$$

It is interesting to see the cancellation in the term $\widehat{b}_1$, where $\Upsilon _1$ did not have a sign, but after multiplication with $({\varvec{\alpha }}{-}{\varvec{\beta }}){\cdot }\nabla E({\varvec{c}})$ it becomes positive and increases the logarithmic mean $\Lambda (k_{\mathrm {fw}}{\varvec{c}}^{\varvec{\alpha }}, k_{\mathrm {bw}}{\varvec{c}}^{\varvec{\beta }})$ to the arithmetic mean $\frac{1}{2}(k_{\mathrm {fw}}{\varvec{c}}^{\varvec{\alpha }}{+}k_{\mathrm {bw}}{\varvec{c}}^{\varvec{\beta }})$. Moreover, the coefficient $b_0$ consists of two terms, the first of which corresponds (up to order $1/V^2$) to the correction ${\varvec{A}}_V$ in (5.4) arising from the improvement of ${\varvec{E}}_V$, while the second term arises from improving the dissipation potential $\Phi ^*_V$, namely via $\Upsilon _0$.

Putting these derivations together, summing over $r=1,\ldots ,R$ different reactions, and dropping all terms of order $1/V^2$, we find the following approximative Fokker–Planck equation:

5.7 $$\begin{aligned} {\dot{\rho }}(t,{\varvec{c}})&= {\mathrm {div}}_{{\varvec{c}}}\Big ( \frac{1}{V} \widehat{\mathbb {K}}_\mathrm {CLE}({\varvec{c}}) \nabla \rho (t,{\varvec{c}}) + \rho (t,{\varvec{c}}) {\varvec{R}}({\varvec{c}})+ \frac{1}{V} \rho (t,{\varvec{c}}){\varvec{B}}({\varvec{c}}) \Big ) \end{aligned}$$

where ${\varvec{R}}({\varvec{c}})={\mathbb {K}}({\varvec{c}}){\mathrm {D}}E({\varvec{c}})$, ${\varvec{B}}({\varvec{c}})=\sum _{r=1}^R \widehat{b}_0^r({\varvec{c}}) ({\varvec{\alpha }}^r{-}{\varvec{\beta }}^r)$, and $\widehat{\mathbb {K}}_\mathrm {CLE}$ is given in (1.6).

The big disadvantage of equation (5.7) is that it is generally no longer a gradient system. However, it may be considered as an equation with an asymptotic gradient flow structure in the sense of [6]. To find the simplest true gradient system that is compatible with the Fokker–Planck equation (5.7), we have to find a true dual dissipation potential $\widehat{\Phi }_V^*$ that is non-negative and coincides with $\Phi ^*_V$ from above to lowest order. To keep the notation light, we again explain the construction for the case of one reaction only and set $\Lambda _0({\varvec{c}})=\Lambda (k_{\mathrm {fw}}{\varvec{c}}^{\varvec{\alpha }}, k_{\mathrm {bw}}{\varvec{c}}^{\varvec{\beta }})$. Our simplest choice is

$$\begin{aligned} \widehat{\Phi }_V^*(\rho ,\xi )&=\int _{\varvec{C}}\Big (\Lambda _0({\varvec{c}}) \rho ({\varvec{c}})+ \frac{1}{V} \Upsilon _0({\varvec{c}}) \rho ({\varvec{c}}) + \frac{1}{V} \Upsilon _1({\varvec{c}})\nabla \rho ({\varvec{c}}){\cdot }({\varvec{\alpha }}{-}{\varvec{\beta }})\\&\quad + \frac{\Upsilon _2({\varvec{c}})}{V^2} \,\rho ({\varvec{c}}) + \frac{\Upsilon _3({\varvec{c}})}{V^2} \frac{\big (\nabla \rho ({\varvec{c}}){\cdot } ({\varvec{\alpha }}{-}{\varvec{\beta }})\big )^2}{ \rho ({\varvec{c}})} \Big ) \big (\nabla \xi ({\varvec{c}}){\varvec{\cdot }}({\varvec{\alpha }}{-}{\varvec{\beta }})\big )^2 \;\!\mathrm {d}{\varvec{c}}, \end{aligned}$$

where the higher-order corrections $\Upsilon _2({\varvec{c}})$ and $\Upsilon _3({\varvec{c}})$ need to be chosen such that $\widehat{\Phi }_V^*(\rho ,\xi )$ is still coercive. Choosing $\theta _1,\theta _2 \in {]0,1[}$ with $\theta _1<\theta _2$, we may require

$$\begin{aligned} \Lambda _0({\varvec{c}}) + \frac{\Upsilon _0({\varvec{c}})}{V} +\frac{\Upsilon _2({\varvec{c}})}{V^2} \ge \theta _2 \Lambda _0({\varvec{c}}) \quad \text {and}\quad 4\theta _1 \Lambda _0({\varvec{c}}) \Upsilon _3({\varvec{c}}) \ge \Upsilon _1({\varvec{c}})^2 \end{aligned}$$

for all $V>1$, so that $\widehat{\Phi }_V^*(\rho ,\xi ) \ge (\theta _2{-}\theta _1)\int _{\varvec{C}}\Lambda _0({\varvec{c}})\rho ({\varvec{c}}) \big (\nabla \xi ({\varvec{c}}){\varvec{\cdot }} ({\varvec{\alpha }}{-}{\varvec{\beta }})\big )^2 \;\!\mathrm {d}{\varvec{c}}$. The bounds for $\Upsilon _2({\varvec{c}})$ and $\Upsilon _3({\varvec{c}})$ hold for the following choices (or any bigger ones)

$$\begin{aligned} \Upsilon _2({\varvec{c}}) = \frac{\Lambda _0({\varvec{c}})}{16(1{-}\theta _2)} \big ({\varvec{\alpha }}{\varvec{\cdot }}{\check{{\varvec{C}}}} {\varvec{\beta }}\big )^2 \quad \text {and}\quad \Upsilon _3({\varvec{c}}) = \frac{1}{4\theta _1\Lambda _0({\varvec{c}})} \,\Upsilon _1({\varvec{c}})^2. \end{aligned}$$

Of course, we fix the energy functional to be the improved entropy functional ${\varvec{E}}_V$ from (5.3), and the gradient system $({\mathscr {P}}({\varvec{C}}),{\varvec{E}}_V,\widehat{\Phi }^*_V)$ has the associated gradient-flow equation ${\dot{\varrho }} = {\mathrm {D}}_\xi \widehat{\Phi }^*_V(\varrho ,{-}{\mathrm {D}}{\varvec{E}}_V(\varrho ))$. With ${\mathrm {D}}{\varvec{E}}_V(\varrho )= \frac{1}{V} (1 {+} \log \rho ) + E + \frac{1}{V} E^V_1$ we find

5.8 $$\begin{aligned} \begin{aligned} {\dot{\rho }}&={\mathrm {div}}\left( \left[ \widehat{a}_0^V({\varvec{c}})\rho + \frac{\widehat{a}_1^V({\varvec{c}})}{V} \nabla _{\varvec{\gamma }}\rho + \frac{\widehat{a}_2^V({\varvec{c}})}{V^2} \frac{(\nabla _{\varvec{\gamma }}\rho )^2}{\rho }+ \frac{\widehat{a}_3^V({\varvec{c}})}{V^3} \frac{(\nabla _{\varvec{\gamma }}\rho )^3}{\rho ^2} \right] {\varvec{\gamma }}\right) \\&\quad \text {with }\ \widehat{a}_0^V= \Lambda _\Upsilon ^V \, \bigg (\nabla _{\varvec{\gamma }}E + \frac{1}{V} \nabla _{\varvec{\gamma }}E^1_V\bigg ), \quad \widehat{a}_1^V= \Lambda _\Upsilon ^V + \Upsilon _1\, \bigg (\nabla _{\varvec{\gamma }}E + \frac{1}{V} \nabla _{\varvec{\gamma }}E^1_V\bigg ), \\ \widehat{a}_2^V&=\Upsilon _1{+}\Upsilon _3 \, \bigg (\nabla _{\varvec{\gamma }}E +\frac{1}{V} \nabla _{\varvec{\gamma }}E^1_V\bigg ), \quad \text { and }\ \widehat{a}_3^V({\varvec{c}})= \Upsilon _3, \\&\quad \text {where } \Lambda _\Upsilon ^V({\varvec{c}})=\Lambda _0({\varvec{c}}) +\frac{\Upsilon _0({\varvec{c}})}{V} + \frac{\Upsilon _2({\varvec{c}})}{V^2}, \quad {\varvec{\gamma }}={\varvec{\alpha }}{-}{\varvec{\beta }}, \quad \text {and } \nabla _{\varvec{\gamma }}f= \nabla f\varvec{\cdot }{\varvec{\gamma }}. \end{aligned} \end{aligned}$$

Because $\nabla _\gamma E^V_1$ is of order 1/*V*, we see that this equation involves terms up to order $1/V^4$, namely through $\widehat{a}{}^V_0$ and through $\widehat{a}{}^V_2/V^2$.

Clearly, our gradient-flow equation (5.8) is much more complicated than those generated by the asymptotic gradient-flow structures in the sense of [6], where higher order terms are simply dropped.

There is also the question of well-posedness for equation (5.8). To have parabolicity of the leading terms we need that the mapping $p \mapsto \frac{1}{V}\widehat{a}^V_1 p + \frac{1}{V^2}\widehat{a}^V_2 p^2 + \frac{1}{V^3}\widehat{a}^V_3 p^3$ is monotone, which amounts to asking that $\widehat{a}^V_1 + 2 \widehat{a}^V_2 q + 3\widehat{a}^V_3 q^2 \ge 0$ for all $q \in {\mathbb {R}}$. This can be always be achieved by making $\Upsilon _2$ very big while keeping $\Upsilon _3$ constant, since $\Upsilon _2$ only enters once via $\widehat{a}{}^V_1$.

### Comparison of Models

To appreciate the positive and negative aspects of the different approximations of the CME, we treat the simplest example, namely the linear RRE on ${\varvec{C}}={[0,\infty [}$:5.9Obviously, we have the explicit solution $c(t)=1+(c(0){-}1\big ) {\mathrm {e}}^{-t}$.

The associated CME for ${\varvec{u}}={\mathscr {P}}({\mathbb {N}}_0)$ is given by

5.10 $$\begin{aligned} {\dot{u}}_n = V u_{n-1} -\big (V{+}n\big ) u_n +(n{+}1) u_{n+1} \quad \text {for } n \in {\mathbb {N}}_0, \end{aligned}$$

where $u_{-1}=0$. Using the linearity in (5.9), which leads to the linearity in *n* of the coefficients in (5.10), we obtain explicit closed form relations of the evolution of the rescaled expectation $\widehat{e}(t):= \frac{1}{V}\sum _{n \in {\mathbb {N}}_0} nu_n(t)$ and variance $\widehat{v}(t):= \frac{1}{V^2} \sum _{n\in {\mathbb {N}}_0} n^2 u_n - \widehat{e}(t)^2$, namely

5.11 $$\begin{aligned} \dot{\widehat{e}}(t)= 1 - \widehat{e}(t) \quad \text { and } \quad \dot{\widehat{v}}(t) = -2 \widehat{v}(t) + \frac{1 + \widehat{e}(t)}{V}. \end{aligned}$$

Moreover, it can be easily checked that for any solution $t \mapsto c(t)$ of the RRE (5.9) the following formula provides an explicit solution of the CME (5.10):

5.12 $$\begin{aligned} u_n(t) = \frac{{\mathrm {e}}^{-c(t)V}}{n!} \,\big (c(t)V\big )^n \quad \text {for } n \in {\mathbb {N}}_0. \end{aligned}$$

Note that this is expression is compatible with the ODEs (5.11) for the moments, since for these Poisson distributions we have $\widehat{e}(t)=c(t)$ and $\widehat{v}(t)=c(t)/V$.

The Liouville equation and the simple Fokker–Planck equation read

$$\begin{aligned} \text {(Lio)}\quad {\dot{\varrho }} =\partial _c\big ( (c{-}1)\varrho \big ) \quad \ \ \text { and (FP)} \quad {\dot{\rho }} = \partial _c \big ( \Lambda (1,c) \,\frac{ \partial _c\rho }{V} + (c{-}1)\rho \big ). \end{aligned}$$

The Fokker–Planck equation for the chemical Langevin equation (cf. (5.6)) takes the form

$$\begin{aligned} (\text {FP}_\mathrm {CLE}) \quad {\dot{\rho }} = \partial _c^2 \Big ( \frac{1{+}c}{2V} \rho \Big )\, + \partial _c \big ( (c{-}1) \rho \big ). \end{aligned}$$

To compare the solutions of (FP) and (FP$_\mathrm {CLE}$) with the true solutions of the CME (5.10), we assume that the solutions can be approximated by Gaußians. In general, for multidimensional Fokker–Planck equations of the form ${\dot{\rho }} = \frac{1}{V} \sum _{ij} \partial _{ij}^2\big ( \rho {\mathbb {M}}_{ij}({\varvec{c}}) \big ) + {\mathrm {div}}\big ( \rho {\varvec{R}}_V\big )$ the ansatz $\rho (t,\cdot ) \sim {\mathrm {N}}({\varvec{a}}(t), \frac{1}{V} {\mathbb {A}}(t))$ with ${\varvec{a}}(t)\in {\mathbb {R}}^d$ and ${\mathbb {A}}(t) \in {\mathbb {R}}^{d{\times }d}_{\mathrm {spd}}$ leads to the necessary conditions

$$\begin{aligned} {\dot{{\varvec{a}}}}(t) = - {\varvec{R}}({\varvec{a}}(t)) \quad \text { and } \quad {\dot{{\mathbb {A}}}}(t)= - {\mathrm {D}}{\varvec{R}}({\varvec{a}}(t)) {\mathbb {A}}(t) - {\mathbb {A}}(t) {\mathrm {D}}{\varvec{R}}({\varvec{a}}(t))^\mathsf {T}+ 2 {\mathbb {M}}({\varvec{a}}(t)), \end{aligned}$$

see [55] for rigorous results of this type. Applying these formulas to (FP$_\mathrm {CLE}$) we obtain

5.13 $$\begin{aligned} {\dot{a}} = 1 - a \quad \text {and} \quad {\dot{A}} = -2 A + 1 + a, \end{aligned}$$

hence the ODEs for *a* and *A*/*V* coincide with those for ${\widehat{e}}$ and ${\widehat{v}}$ in (5.11).

A similar argument indicates that solutions to (FP) are well approximated by Gaußians with mean $a_V$ and variance $A_V$ satisfying

5.14 $$\begin{aligned} {\dot{a}}_V = 1 - a_V + \frac{1}{V} \partial _2 \Lambda (1, a_V) \quad \text {and} \quad {\dot{A}}_V = -2 A_V + 2\Lambda (1, a_V). \end{aligned}$$

On the one hand, this clearly indicates that (FP$_\mathrm {CLE}$) provides a better approximation to the CME for $t\in [0,T]$. By formally passing to the limit $V \rightarrow \infty$ in (5.14), we see that the ODE for $a_V$ is asymptotically correct. This is not the case for the ODE for $A_V$, since the arithmetic mean in (5.13) is replaced by the logarithmic mean in (5.14). However, the error of $\Lambda (1,c)$ compared to $\frac{1}{2}(1{+}c)$ is less than 10 % for $c\in [1/3,3]$ and it converges to 0 for $c\rightarrow 1$, i.e., in the limit $t\rightarrow \infty$. Equations (5.13) are consistent with Kurtz’ central limit theorem, which asserts that the normalized process $\frac{1}{V} {\varvec{N}}^V(t)$ has fluctuations around ${\varvec{c}}(t)$ of order $1/\sqrt{V}$, and the rescaled process $\sqrt{V}\big ( \frac{1}{V} {\varvec{N}}^V(t) -{\varvec{c}}(t)\big )$ converges to a Gaußian process $t\mapsto {\varvec{V}}(t)$ with covariance matrix ${\mathbb {A}}$ satisfying ${\dot{{\mathbb {A}}}}(t) = -{\mathrm {D}}{\varvec{R}}({\varvec{c}}(t)){\mathbb {A}}(t) - {\mathbb {A}}(t){\mathrm {D}}{\varvec{R}}({\varvec{c}}(t))^\mathsf {T}+ 2 \widehat{\mathbb {K}}_\mathrm {CLE}({\varvec{c}}(t))$, see, e.g., [34, Eq.  (1.9)].

On the other hand, (FP) makes a better prediction for the equilibrium distribution that is attained for $t\rightarrow \infty$. For (FP$_\mathrm {CLE}$) we have the unique steady state

$$\begin{aligned} \rho ^\text {eq,CLE}_V(c)= \frac{1}{Z^\mathrm {CLE}_V} \,{\mathrm {e}}^{-V \widetilde{E}(c)} \quad \text {with } \widetilde{E}(c)= \int _1^c \tfrac{2b{-}2{+} 1/V}{b+1} \;\!\mathrm {d}b =2c{-}2{-}(4{-}\tfrac{1}{V}) \log \tfrac{1{+}c}{2}. \end{aligned}$$

Thus, $\widetilde{E}$ grows only like *c*, such that $\rho ^\text {eq,CLE}_V$ decays exponentially only. In contrast, the equilibrium $\rho ^\text {eq,FP}_V= Z_V^{-1} {\mathrm {e}}^{-V E(c)}$ of (FP) produces the correct super-exponential decay of the stationary Poisson distribution equation for the CME (5.10).

### Approximation via Cosh-Type Gradient Structure

The derivation of a gradient structure (4.3) for the Liouville equation (4.1) can be repeated very similarly by starting from the cosh-type gradient structure introduced in [44], see Proposition 3.8. We do not give the details here but provide the result only.

Starting from the cosh-type dual dissipation potential ${\varvec{\Psi }}^*_{\cosh ,V}$ defined in (3.8) instead of the quadratic dual potential $\Psi ^*_V$ defined in (4.5) we obtain the counterparts to Propositions 4.5 and but now with

$$\begin{aligned} {\varvec{\Psi }}^*_{\cosh ,\mathrm {Lio}}(\varrho ,\xi ):= \int _{{\varvec{c}}\in {\varvec{C}}} \sum _{r=1}^R \kappa ^r \Big (\frac{{\varvec{c}}^{{\varvec{\alpha }}^r}}{{\varvec{c}}_*^{{\varvec{\alpha }}^r}} \, \frac{{\varvec{c}}^{{\varvec{\beta }}^r}}{{\varvec{c}}_*^{{\varvec{\beta }}^r}} \Big )^{1/2} {\mathsf {C}}^*\big ( ( {\varvec{\beta }}^r{-}{\varvec{\alpha }}^r) \varvec{\cdot }\nabla _{\varvec{c}}\xi ({\varvec{c}})\big ) {\mathrm {d}}\varrho ({\varvec{c}}) . \end{aligned}$$

Without any need to justify the approximation procedure in the sense of Sect. 4.2 we easily obtain the following result.

#### Proposition 5.1

(Cosh-type gradient structure for the Liouville equation) The Liouville equation (4.1) has the gradient structure $({\mathscr {P}}({\varvec{C}}), {\varvec{E}},{\varvec{\Psi }}^*_{\cosh ,\mathrm {Lio}})$ with ${\varvec{E}}$ from (4.2) and ${\varvec{\Psi }}^*_{\cosh ,\mathrm {Lio}}$ from above.

#### Proof

The result follows by using ${\mathrm {D}}{\mathcal {E}}(\varrho )(\cdot ) = E(\cdot )$, $\nabla _{\varvec{c}}E({\varvec{c}})=\big ( \log (c_i/c^*_i)\big )_{i=1,\ldots ,I}$, and

$$\begin{aligned}&{\mathrm {D}}_\xi {\varvec{\Psi }}^*_{\cosh ,\mathrm {Lio}}\big (\varrho , {-}{\mathrm {D}}{\mathcal {E}}(\varrho )\big ) [\eta ] \\&\quad = \int _{\varvec{C}}\sum _{r=1}^R \kappa ^r \Big (\frac{{\varvec{c}}^{{\varvec{\alpha }}^r}}{{\varvec{c}}_*^{{\varvec{\alpha }}^r}} \, \frac{{\varvec{c}}^{{\varvec{\beta }}^r}}{{\varvec{c}}_*^{{\varvec{\beta }}^r}} \Big )^{1/2} ({\mathsf {C}}^*)'\big ({-}{\varvec{\gamma }}^r\varvec{\cdot }\big ( \log \frac{c_i}{c^*_i}\big )_{i} \big )\big [ {\varvec{\gamma }}^r\varvec{\cdot }\nabla \eta \big ] {\mathrm {d}}\varrho ({\varvec{c}}), \end{aligned}$$

where ${\varvec{\gamma }}^r = {\varvec{\alpha }}^r {-} {\varvec{\beta }}^r$. Using $\sqrt{ab}\,({\mathsf {C}}^*)'\big (\log (a/b)\big )=a{-}b$ and the definition of ${\varvec{R}}$ gives ${\mathrm {D}}_\xi {\varvec{\Psi }}^*_{\cosh ,\mathrm {Lio}}\big (\varrho , {-}{\mathrm {D}}{\mathcal {E}}(\varrho )\big ) [\eta ]= -\int _{\varvec{C}}{\varvec{R}}({\varvec{c}})\varvec{\cdot }\nabla \eta \;\!\mathrm {d}\varrho ({\varvec{c}})$ which is the desired right-hand side of (4.1) when testing with $\eta$ and integrating by parts. $\square$

As in the case of quadratic gradient structure for the Liouville equation we may consider the first-order correction to obtain a Fokker–Planck equation. For this we insert the improved energy ${\varvec{E}}_V$ defined in (5.3) into the dissipation potential ${\varvec{\Psi }}^*_{\cosh ,V}$ (cf. (3.8)) to obtain a quasilinear Fokker–Planck-type equation, namely ${\dot{\varrho }} = {\mathrm {D}}_\xi {\varvec{\Psi }}^*_{\cosh ,V} \big (\varrho ,{-} {\mathrm {D}}{\varvec{E}}_V (\varrho )\big )$. Using the abbreviations $a_r:=\frac{{\varvec{c}}^{{\varvec{\alpha }}^r}}{{\varvec{c}}_*^{{\varvec{\alpha }}^r}}$ and $b_r:= \frac{{\varvec{c}}^{{\varvec{\beta }}^r}}{{\varvec{c}}_*^{{\varvec{\beta }}^r}}$ we find (note $\nabla _{\varvec{c}}E^V_1({\varvec{c}})=O(1/V)$)

$$\begin{aligned}&{\mathrm {D}}_\xi {\varvec{\Psi }}^*_{\cosh ,V} \big (\varrho ,{-} {\mathrm {D}}{\varvec{E}}_V (\varrho )\big ) \ = \ {\mathrm {D}}_\xi {\varvec{\Psi }}^*_{\cosh ,V} \Big (\varrho , -\frac{1}{V} \log \rho {-} E{-} \frac{1}{V} E^V_1 \Big ) \\&\quad = {\mathrm {div}}\bigg (\rho \sum _{r=1}^R \kappa ^r \sqrt{a_rb_r} \left[ ({\mathsf {C}}^*)'\big (\log \frac{b_r}{a_r}\big ){\varvec{\gamma }}^r + ({\mathsf {C}}^*)''\big (\log \frac{b_r}{a_r} \big ) \frac{{\varvec{\gamma }}^r{\varvec{\cdot }}\nabla \rho }{V \rho } {\varvec{\gamma }}^r\right] +O(1/V^2) \bigg ). \end{aligned}$$

Using the identities $\sqrt{ab}\,({\mathsf {C}}^*)'\big (\log (b/a)\big )=b-a$ and $\sqrt{ab}\,({\mathsf {C}}^*)''\big (\log (b/a)\big ) = (a{+}b)/2$ the FP equation has the expansion

$$\begin{aligned} {\dot{\rho }}(t,{\varvec{c}}) = {\mathrm {div}}_{{\varvec{c}}}\Big ( \rho (t,{\varvec{c}}) {\varvec{R}}({\varvec{c}})+ \frac{1}{V} \widehat{\mathbb {K}}_\mathrm {CLE}({\varvec{c}}) \nabla _{{\varvec{c}}} \rho (t,{\varvec{c}}) +O(1/V^2)_{V\rightarrow \infty } \Big ) \end{aligned}$$

where $\widehat{\mathbb {K}}_\mathrm {CLE}$ is exactly the same as obtained in (1.6) by a completely different approach.

## Hybrid Models

We show in this section how the different gradient structures for RRE, for CME, and for the FPE can be combined to obtain hybrid models, which are combinations of several models depending on the desired accuracy. The importance here is to use the proper rescalings in terms of the volume *V* to make the different descriptions compatible. We do not consider a full theory, but highlight first the general strategy of model reduction for gradient systems in Sect. 6.1 and then illustrate this by a simple example in Sect. 6.2. A nontrivial case of a rigorous coarse graining in this spirit is given in [43, 47], where a linear RRE with a small parameter $\varepsilon$ is considered. The elimination of the fast relaxations in the time scale $\varepsilon$ leads to a coarse-grained gradient system.

In Sect. 6.3 we discuss the general coupling of the FPE to a RRE and the similar coupling of the CME to a RRE, both leading to so-called mean-field equations, where a linear equation for a probability density is nonlinearly coupled to an ODE. Finally, we discuss the mixed discrete and continuous description, where the CME is used for small numbers of particles and the FPE is used for larger numbers.

### Coarse Graining for Gradient Systems

If a gradient system $({\mathsf {X}},{\mathsf {E}}_X,\Psi _X)$ is more complicated than what is needed, one is interested in approximating the system by a simpler model that still contains the most important features. We explain how this can be done while keeping the gradient structure.

We assume that the relevant states ${\mathsf {x}}\in {\mathsf {X}}$ can be described by states ${\mathsf {y}}\in {\mathsf {Y}}$ and that there is a reconstruction mapping ${\mathsf {x}}= \Phi ({\mathsf {y}})$, i.e., $\Phi ({\mathsf {Y}})$ is a subset (or submanifold) of ${\mathsf {X}}$. We now pull back the gradient structure $({\mathsf {X}},{\mathsf {E}}_X,\Psi _X)$ to an approximative gradient structure $({\mathsf {Y}},{\mathsf {E}}_Y,\Psi _Y)$. The natural approach is to restrict the energy functional and the (primal) dissipation potential as follows:

6.1 $$\begin{aligned} {\mathsf {E}}_{\mathsf {Y}}({\mathsf {y}})={\mathsf {E}}_{\mathsf {X}}(\Phi ({\mathsf {y}})) \quad \text {and} \quad \Psi _{\mathsf {Y}}({\mathsf {y}},{\dot{{\mathsf {y}}}}):=\Psi _{\mathsf {X}}(\Phi ({\mathsf {y}}),{\mathrm {D}}\Phi ({\mathsf {y}}){\dot{{\mathsf {y}}}}). \end{aligned}$$

The solutions ${\mathsf {y}}:[0,T]\rightarrow {\mathsf {Y}}$ of the coarse-grained gradient system $({\mathsf {Y}},{\mathsf {E}}_{\mathsf {Y}},\Psi _{\mathsf {Y}})$ will provide good approximations $\widehat{x}: t\mapsto \Phi ({\mathsf {y}}(t))\in {\mathsf {X}}$ of the true solutions of the full GS $({\mathsf {X}},{\mathsf {E}}_X,\Psi _X)$, if the set $\Phi ({\mathsf {Y}})$ approximates a flow-invariant subset of ${\mathsf {X}}$.

In reaction systems, the primal dissipation potential $\Psi _{\mathsf {X}}$ is usually not known explicitly. Hence, it is desirable to have a method for reducing the dual dissipation potential $\Psi _{\mathsf {X}}^*$ directly to $\Psi _{\mathsf {Y}}^*$, in the case where $A = {\mathrm {D}}\Phi ({\mathsf {y}}) : {\mathsf {Y}}\rightarrow {\mathsf {X}}$ is injective but its adjoint mapping $A^*:{\mathsf {X}}^*\rightarrow {\mathsf {Y}}^*$ has a large kernel. The following exact result will be the motivation for our modeling approximations in the subsequent subsections.

#### Proposition 6.1

Consider reflexive Banach spaces ${\mathsf {X}}$ and ${\mathsf {Y}}$ and a real-valued dissipation potential $\Psi :{\mathsf {X}}\rightarrow {[0,\infty [}$ (i.e. lower semicontinuous, convex, and $\Psi (0)=0$) that is superlinear, i.e. $\Psi ({\mathsf {v}})/ \Vert {\mathsf {v}}\Vert _{\mathsf {X}}\rightarrow \infty$ for $\Vert {\mathsf {v}}\Vert _{\mathsf {X}}\rightarrow \infty$. Assume that the bounded linear operator $A : {\mathsf {Y}}\rightarrow {\mathsf {X}}$ has closed range. Then the dissipation potential $\widetilde{\Psi }:{\mathsf {Y}}\rightarrow {[0,\infty [};\ {\mathsf {y}}\mapsto \Psi (A{\mathsf {y}})$ satisfies

6.2 $$\begin{aligned} \widetilde{\Psi }{}^*(\eta )= \inf \big \{\, \Psi ^*(\xi ) \, \big | \, A^*\xi =\eta \,\big \} \quad \text { for all } \eta \in {\mathsf {Y}}^*, \end{aligned}$$

where we use the convention $\inf \emptyset = \infty$.

#### Proof

For the proof we use the saddle-point theory in [14, Ch. VI.2].

Fix $\eta \in {\mathsf {Y}}^*$ and assume first that $\eta \notin {{\,\mathrm{Ran}\,}}(A^*)$. Since ${{\,\mathrm{Ran}\,}}(A) \subset {\mathsf {X}}$ is closed, the Closed Range Theorem yields that ${{\,\mathrm{Ran}\,}}(A^*) \subset {\mathsf {Y}}^*$ is closed as well, and ${{\,\mathrm{Ran}\,}}(A^*) = {{\,\mathrm{Ker}\,}}(A)^\perp$. Consequently, there exists ${\tilde{{\mathsf {y}}}} \in {{\,\mathrm{Ker}\,}}(A)$ such that $\langle {\eta , {\tilde{{\mathsf {y}}}}}\rangle \ne 0$, and we obtain

$$\begin{aligned} \widetilde{\Psi }{}^*(\eta ) = \sup _{{\mathsf {y}}\in {\mathsf {Y}}} \big ( \langle \eta , {\mathsf {y}}\rangle - \Psi (A{\mathsf {y}}) \big ) \ge \sup _{\lambda \in {\mathbb {R}}} \big ( \lambda \langle \eta , {\tilde{{\mathsf {y}}}} \rangle - \Psi (\lambda A {\tilde{{\mathsf {y}}}}) \big ) = \infty . \end{aligned}$$

This yields (6.2), since the right-hand side is clearly infinite as well.

Fix now $\eta \in {{\,\mathrm{Ran}\,}}(A^*)$ and define the Lagrangian function $L:{\mathsf {X}}{\times }{\mathsf {X}}^*\rightarrow {[{-}\infty ,\infty [}$ via

$$\begin{aligned} L({\mathsf {x}},\xi ) = -\langle \xi ,x\rangle + \Psi (x) - \chi ^*(\xi ) \quad \text {with }\chi ^*(\xi )=\left\{ \begin{array}{cl} 0 &{}\text {for }A^*\xi =\eta ,\\ \infty &{}\text {otherwise}. \end{array}\right. \end{aligned}$$

For notational convenience we set

$$\begin{aligned} h({\mathsf {x}})=\sup _{\xi \in {\mathsf {X}}^*} L({\mathsf {x}},\xi ), \quad g(\xi )= \inf _{{\mathsf {x}}\in {\mathsf {X}}} L({\mathsf {x}},\xi ), \quad P:=\inf _{\mathsf {X}}h, \quad D:= \sup _{{\mathsf {X}}^*} g. \end{aligned}$$

Classical duality theory yields the trivial inequality $P \ge D$. Clearly, $L(\cdot ,\xi )$ is convex and lower semicontinuous, whereas $L({\mathsf {x}},\cdot )$ is concave and upper semicontinuous, since the boundedness of $A^*$ implies that $\big \{\, \xi \in {\mathsf {X}}^* \, \big | \, A^*\xi =\eta \,\big \}$ is closed.

Using $\eta \in {{\,\mathrm{Ran}\,}}(A^*)$, we find $\xi _\eta \in {\mathsf {X}}^*$ with $A^* \xi _\eta = \eta$, so that our assumptions guarantee the coercivity of ${\mathsf {x}}\mapsto L({\mathsf {x}}, \xi _\eta ) \in {\mathbb {R}}$. Hence, we can apply [14, Chap. VI, Prop. 2.3], which shows that there is no duality gap:

6.3 $$\begin{aligned}&P=\inf _{{\mathsf {x}}\in {\mathsf {X}}}h({\mathsf {x}}) = \min _{{\mathsf {x}}\in {\mathsf {X}}} \Big (\sup _{\xi \in {\mathsf {X}}^*} L({\mathsf {x}},\xi )\Big ) \ = \ \sup _{\xi \in {\mathsf {X}}^*} \Big ( \inf _{{\mathsf {x}}\in {\mathsf {X}}} L({\mathsf {x}},\xi )\Big ) = \sup _{\xi \in {\mathsf {X}}^*} g(\xi ) = D. \end{aligned}$$

We relate *P* and *D* with the two sides in our desired formula (6.2). On the one hand,

$$\begin{aligned} h({\mathsf {x}})&= \sup _{\xi \in {\mathsf {X}}^*} L({\mathsf {x}},\xi ) = \Psi ({\mathsf {x}}) + \sup _{\xi \in {\mathsf {X}}^*} \big ( \langle \xi ,{-}{\mathsf {x}}\rangle - \chi ^*(\xi )\big ) \\&= \Psi ({\mathsf {x}}) -\langle \xi _\eta ,{\mathsf {x}}\rangle + \mu ({-}{\mathsf {x}}) \ \text {with } \mu ({\mathsf {x}}):= \sup _{\zeta \in {\mathsf {X}}^*} \big ( \langle \zeta ,{\mathsf {x}}\rangle - \delta _0(A^*\zeta )\big ), \end{aligned}$$

where in the last step we have substituted $\xi = \xi _\eta +\zeta$ with $A^*\xi _\eta = \eta$ and introduced $\delta _0(\widetilde{\eta })=0$ for $\widetilde{\eta }=0$ and $\infty$ otherwise. Thus, we conclude

$$\begin{aligned} h({\mathsf {x}}) = \Psi (A{\mathsf {y}}) - \langle \eta ,{\mathsf {y}}\rangle \ \text { for } {\mathsf {x}}=A{\mathsf {y}}\quad \text {and} \quad h({\mathsf {x}})=\infty \ \text { for } {\mathsf {x}}\not \in {{\,\mathrm{Ran}\,}}(A). \end{aligned}$$

Thus, taking the minimum over all of ${\mathsf {X}}$ is the same as taking it over ${{\,\mathrm{Ran}\,}}(A)$, namely

$$\begin{aligned} P = \inf _{{\mathsf {x}}\in {\mathsf {X}}} h({\mathsf {x}}) = \inf _{{\mathsf {y}}\in {\mathsf {Y}}} \big ( \Psi (A{\mathsf {y}})-\langle \eta , {\mathsf {y}}\rangle \big ) = - \widetilde{\Psi }{}^*(\eta ). \end{aligned}$$

On the other hand, the definition of $g(\xi )=\inf _{{\mathsf {x}}\in {\mathsf {X}}} L({\mathsf {x}}, \xi )$ immediately gives $g(\xi )=-\Psi ^*(\xi )-\chi ^*(\xi )$. Hence, we arrive at

$$\begin{aligned} D = \sup _{\xi \in {\mathsf {X}}^*} g(\xi ) = - \inf _{\xi \in {\mathsf {X}}^*} \big (\Psi ^*(\xi )+\chi ^*(\xi )\big )= - \inf \big \{\, \Psi ^*(\xi ) \, \big | \, A^*\xi =\eta \,\big \} . \end{aligned}$$

As a result, formula (6.2) follows from $P=D$. $\square$

In our applications below (as well as in most others) the explicit minimization in (6.2) is too complicated to be executed. However, as the coarse-graining mapping through $\Phi$ is usually only an approximation, it may suffice to approximate the minimizers suitably. In general, one has to find an approximation $\xi ={\mathsf {M}}({\mathsf {y}},\eta ) \in {\mathsf {X}}^*$ and sets

6.4 $$\begin{aligned}&\Psi ^*_{\mathsf {Y}}({\mathsf {y}},\eta )= \Psi ^*_{\mathsf {X}}(\Phi ({\mathsf {y}}),{\mathsf {M}}({\mathsf {y}},\eta )) \quad \text {or} \quad \tfrac{1}{2} \langle \eta , \widetilde{\mathsf {K}}_{\mathsf {Y}}({\mathsf {y}}) \eta \rangle = \langle {\mathsf {L}}({\mathsf {y}}) \eta , {\mathsf {K}}_{\mathsf {X}}(\Phi ({\mathsf {y}})) {\mathsf {L}}({\mathsf {y}}) \eta \rangle , \end{aligned}$$

where ${\mathsf {L}}({\mathsf {y}}):{\mathsf {Y}}^*\rightarrow {\mathsf {X}}^*$ is the linear version of ${\mathsf {M}}$. Of course, when constructing ${\mathsf {M}}$ or ${\mathsf {L}}$ one should keep (6.2) in mind to preserve all interesting properties inherited by the coarse-graining process.

#### Remark 6.2

(*Coarse-graining for fast-slow reaction systems*) Particular cases of rigorous coarse graining are discussed in [43] and [47], where linear and nonlinear fast-slow RRE are considered, respectively. However, the approach is somehow opposite: if $\varepsilon >0$ is the time scale of the fast reactions, then in the limit $\varepsilon \rightarrow 0$ a linear constraint is imposed on ${\mathrm {D}}E({\varvec{c}})$ defining a slow manifold ${\varvec{c}}=\Phi ({\mathsf {y}})$, and ${\varvec{c}}$ is constraint to lie on this linear or nonlinear manifold. The reduced or coarse-grained gradient system is then given by ${\mathsf {E}}_{\mathsf {Y}}({\mathsf {y}})=E(\Phi (c))$ and $\Psi ^*_{\mathsf {Y}}({\mathsf {y}},\eta ) = \Psi ^*_\text {slow}(\Phi ({\mathsf {y}}),Q^*\eta )$, which means $\Psi _{\mathsf {Y}}({\mathsf {y}},{\mathsf {v}}) = \inf \big \{\, \Psi _\text {slow}(\Phi ({\mathsf {y}}),{\varvec{v}}) \, \big | \, Q{\varvec{v}}={\mathsf {v}} \,\big \}$, see [43, Thm. 5.6] and [47, Prop. 4.2].

### A Simple Example: from CME to RRE

We apply the above idea with $({\mathsf {X}},{\mathsf {E}}_{\mathsf {X}},\Psi ^*_{\mathsf {X}})$ being $({\mathscr {P}}({\mathcal {N}}),{\mathcal {E}}_V,{\mathcal {K}}_V)$ and with $({\mathsf {Y}},{\mathsf {E}}_{\mathsf {Y}},\Psi _{\mathsf {Y}})$ being $({\varvec{C}},E,{\mathbb {K}})$. The embedding mapping $\Phi _V : {\varvec{C}}\rightarrow {\mathscr {P}}({\mathcal {N}})$ is given by the Poisson distributions

$$\begin{aligned} \Phi _V({\varvec{c}}):= \Big ( {\mathrm {e}}^{-V|{\varvec{c}}|_1} \,\frac{(V{\varvec{c}})^{\varvec{n}}}{{\varvec{n}}!}\Big )_{{\varvec{n}}\in {\mathcal {N}}} ,\ \text { where }|{\varvec{c}}|_1=\sum _{1}^I c_i. \end{aligned}$$

In the simple example ${\dot{c}}=1-c$ treated in Sect. 5.4 the image of $\Phi _V$ defines an exactly invariant submanifold, but this is no longer true for nonlinear equations or systems. Nevertheless our construction provides the surprising identity

$$\begin{aligned} {\mathsf {E}}_{\mathsf {Y}}({\varvec{c}})= {\mathcal {E}}_V(\Phi _V({\varvec{c}})) = E({\varvec{c}}), \end{aligned}$$

with the old *E* defined in (2.9) which is independent of *V*.

To reduce the dual dissipation potential $\Psi _{\mathsf {X}}$ defined via ${\mathcal {K}}_V$ we use the derivative

$$\begin{aligned} {\mathrm {D}}\Phi _V({\varvec{c}}) {\varvec{w}}= \left( {\mathrm {e}}^{-V|{\varvec{c}}|_1} \,\frac{(V{\varvec{c}})^{\varvec{n}}}{{\varvec{n}}!} \sum _{i=1}^I \Big (\frac{n_i}{c_i}-V \Big )w_i \right) _{{\varvec{n}}\in {\mathcal {N}}} . \end{aligned}$$

Thus, the adjoint operator ${\mathrm {D}}\Phi _V({\varvec{c}})^*$ maps ${\varvec{\mu }}=(\mu _{\varvec{n}})$ to ${\varvec{\zeta }}=(\zeta _i)_{i=1,\ldots ,I}$ via

$$\begin{aligned} {\varvec{\mu }}\mapsto {\varvec{\zeta }}= {\mathrm {D}}\Phi _V({\varvec{c}})^*{\varvec{\mu }}= \left( \sum _{{\varvec{n}}\in {\mathcal {N}}} {\mathrm {e}}^{-V|{\varvec{c}}|_1} \,\frac{(V{\varvec{c}})^{\varvec{n}}}{{\varvec{n}}!} \Big (\frac{n_i}{c_i}-V \Big ) \mu _{\varvec{n}}\right) _{i=1,\ldots ,I} . \end{aligned}$$

In general, one is not able to solve the minimization problem (6.2) that produces $\Psi ^*_{\mathsf {Y}}$ from $\Psi _{\mathsf {X}}^*$, so instead we construct a linear mapping ${\varvec{\zeta }}\mapsto \widetilde{\varvec{\mu }}={\mathsf {M}}_V({\varvec{c}}){\varvec{\zeta }}$ that approximates the minimizer for $V\rightarrow \infty$ and satisfies ${\varvec{\zeta }}= {\mathrm {D}}\Phi _V({\varvec{c}})^*{\mathsf {M}}_V({\varvec{c}}){\varvec{\zeta }}$. Indeed, we search for $\widetilde{\varvec{\mu }}$ in the linear form $\widetilde{\varvec{\mu }}^{\varvec{a}}_{\varvec{n}}= {\varvec{a}}\varvec{\cdot }{\varvec{n}}$ for ${\varvec{n}}\in {\mathcal {N}}$ and obtain

$$\begin{aligned} {\mathrm {D}}\Phi _V({\varvec{c}})^*\widetilde{\varvec{\mu }}^{\varvec{a}}&={\mathrm {D}}\Phi _V({\varvec{c}})^*( {\varvec{a}}{\varvec{\cdot }} {\varvec{n}})_{{\varvec{n}}\in {\mathcal {N}}} = \left( \sum _{{\varvec{n}}\in {\mathcal {N}}} {\mathrm {e}}^{-V|{\varvec{c}}|_1} \,\frac{(V{\varvec{c}})^{\varvec{n}}}{{\varvec{n}}!} \Big (\frac{n_i}{c_i}-V \Big ) \sum _{j=1}^I a_j n_j \right) _{i=1,\ldots ,I}\\&=\left( \sum _{j=1}^I \sum _{{\varvec{n}}\in {\mathcal {N}}} {\mathrm {e}}^{-V|{\varvec{c}}|_1} \,\frac{(V{\varvec{c}})^{\varvec{n}}}{{\varvec{n}}!} \Big (\frac{n_ia_jn_j}{c_i}-Va_jn_j \Big )\right) _{i=1,\ldots ,I} \\&=\left( \sum _{j=1}^I \Big (V^2\frac{c_i a_jc_j}{c_i} +\delta _{ij}Va_i - V^2a_jc_j \Big ) \right) _{i=1,\ldots ,I} \ = \ V{\varvec{a}}, \end{aligned}$$

where we used the identities $\sum _{{\varvec{n}}\in {\mathcal {N}}} {\mathrm {e}}^{-V|{\varvec{c}}|_1} \,\frac{(V{\varvec{c}})^{\varvec{n}}}{{\varvec{n}}!} n_i = Vc_i$ and $\sum _{{\varvec{n}}\in {\mathcal {N}}} {\mathrm {e}}^{-V|{\varvec{c}}|_1} \,\frac{(V{\varvec{c}})^{\varvec{n}}}{{\varvec{n}}!} n_i n_j= V^2c_ic_j + \delta _{ij} Vc_i$. Thus, we choose the simple operator ${\mathsf {M}}_V$ of the form

$$\begin{aligned} {\varvec{\zeta }}\ \mapsto \ {\varvec{\mu }}={\mathsf {M}}_V({\varvec{c}}) {\varvec{\zeta }}= \big (\frac{1}{V} {\varvec{\zeta }}\varvec{\cdot }{\varvec{n}}\big )_{{\varvec{n}}\in {\mathcal {N}}}. \end{aligned}$$

For inserting $\mu ={\mathsf {M}}_V({\varvec{c}}){\varvec{\zeta }}$ and ${\varvec{u}}=\Phi _V({\varvec{c}})$ into the full dual dissipation potential $\Psi ^*_{\mathsf {X}}$, we use the form (4.5b) and the relations $({\mathsf {M}}_V({\varvec{c}}) {\varvec{\zeta }})_{{\varvec{n}}{+}{\varvec{\alpha }}} -({\mathsf {M}}_V({\varvec{c}}) {\varvec{\zeta }})_{{\varvec{n}}{+}{\varvec{\beta }}} = \frac{1}{V} {\varvec{\zeta }}\varvec{\cdot }({\varvec{\alpha }}{-}{\varvec{\beta }})$ and

$$\begin{aligned} \!\;{\mathbb {B}}_{V}^{{\varvec{\beta }}}\!({\varvec{n}})\!\; \big (\Phi _V({\varvec{c}})\big )_{{\varvec{n}}+{\varvec{\beta }}} =\frac{V ({\varvec{n}}{+}{\varvec{\beta }})!}{V^{|{\varvec{\beta }}|}{\varvec{n}}!}\, {\mathrm {e}}^{-V|{\varvec{c}}|_1}\frac{(V{\varvec{c}})^{{\varvec{n}}+{\varvec{\beta }}} }{({\varvec{n}}{+}{\varvec{\beta }})!} = V\, {\mathrm {e}}^{-V|{\varvec{c}}|_1}\frac{(V{\varvec{c}})^{\varvec{n}}}{{\varvec{n}}!}\;{\varvec{c}}^{{\varvec{\beta }}}. \end{aligned}$$

With this, we find an approximation of the reduced dual dissipation potential $\Psi ^*_{\mathsf {Y}}$, namely

$$\begin{aligned} \Psi ^*_{\mathsf {Y}}({\varvec{c}},{\varvec{\zeta }})&:= \Psi ^*_{\mathsf {X}}\big (\Phi _V({\varvec{c}}), \mathsf M_V({\varvec{c}}) {\varvec{\zeta }}\big ) \\&= \frac{V}{2}\sum _{r=1}^R \sum _{{\varvec{n}}\in {\mathcal {N}}} V \, {\mathrm {e}}^{-V|{\varvec{c}}|_1}\frac{(V{\varvec{c}})^{\varvec{n}}}{{\varvec{n}}!} \, \Lambda \big (k^r_{\mathrm {fw}}{\varvec{c}}^{{\varvec{\alpha }}^r}, k^r_{\mathrm {bw}}{\varvec{c}}^{{\varvec{\beta }}^r}\big ) \bigg ( \frac{({\varvec{\beta }}^r{-}{\varvec{\alpha }}^r){\varvec{\cdot }}{\varvec{\zeta }}}{V}\bigg )^2 \\&= \frac{1}{2} \sum _{r=1}^R \Lambda \big (k^r_{\mathrm {fw}}{\varvec{c}}^{{\varvec{\alpha }}^r}, k^r_{\mathrm {bw}}{\varvec{c}}^{{\varvec{\beta }}^r}\big ) \big ( ({\varvec{\beta }}^r{-}{\varvec{\alpha }}^r){\varvec{\cdot }}{\varvec{\zeta }}\big )^2 \ = \ \frac{1}{2} {\varvec{\zeta }}\varvec{\cdot }{\mathbb {K}}({\varvec{c}}) {\varvec{\zeta }}. \end{aligned}$$

Thus, the gradient system $({\mathsf {Y}},{\mathsf {E}}_{\mathsf {Y}},\Psi ^*_{\mathsf {Y}})$ obtained by the abstract reduction procedure is exactly given by $({\varvec{C}},E,{\mathbb {K}})$, which is the gradient system for the RRE (2.2) studied in Theorem 2.2.

### Coupling a RRE to a Fokker–Planck Equation

In many applications one is interested in the microscopic description of some variables $c_j$, while other variables $c_i$ can be described more macroscopically. We first start from the simplified FPE (5.5) as the gradient system $({\mathscr {P}}({\varvec{C}}), \widetilde{\varvec{E}}_V, {\varvec{K}})$ and partition the components of ${\varvec{c}}$ into *stochastic* and *macroscopic* parts, ${\varvec{c}}_{\mathrm {s}}$ and ${\varvec{c}}_{\mathrm {m}}$ respectively, via

$$\begin{aligned}&{\varvec{c}}=(c_1,\ldots ,c_J,c_{J+1},\ldots ,c_I) = ({\varvec{c}}_{\mathrm {s}},{\varvec{c}}_{\mathrm {m}}) \quad \text {with }\\&{\varvec{c}}_{\mathrm {s}}:=(c_1,\ldots ,c_J)\in {\varvec{C}}_{\mathrm {s}}:={[0,\infty [}^J \text { and }{\varvec{c}}_{\mathrm {m}}:=(c_{J+1},\ldots ,c_I)\in {\varvec{C}}_{\mathrm {m}}:={[0,\infty [}^{I-J}, \end{aligned}$$

In the notation of Sect. 6.1 we let ${\mathsf {X}}={\mathscr {P}}({\varvec{C}}_{\mathrm {s}}{\times }{\varvec{C}}_{\mathrm {m}})$ and ${\mathsf {Y}}={\mathscr {P}}({\varvec{C}}_{\mathrm {s}}){\times }{\varvec{C}}_{\mathrm {m}}$.

For the mapping $\Phi :{\mathsf {Y}}\rightarrow {\mathsf {X}}$ we choose the product ansatz

$$\begin{aligned} \Phi _V(\varrho _{\mathrm {s}},\widehat{\varvec{c}}_{\mathrm {m}})(\mathrm{d}c_1,\ldots ,\mathrm{d}c_I):= \varrho _{\mathrm {s}}(\mathrm{d}{\varvec{c}}_{\mathrm {s}}) \prod _{j=J+1}^I {\mathsf {W}}(c_j;\widehat{c}_j,V) \;\!\mathrm {d}c_j, \end{aligned}$$

where the probability densities ${\mathsf {W}}(\cdot ;\widehat{a},V)$ are given as follows:

$$\begin{aligned} {\mathsf {W}}(a;\widehat{a},V)&:=\frac{1}{\widehat{a}\,Z(V\widehat{a})} \exp \big ({-}V \widehat{a}\, \lambda _{\mathrm {B}}(a/\widehat{a})\big ) \ \text { with } Z(v):=\int _0^\infty \exp \big ({-}v\, \lambda _{\mathrm {B}}(z)\big ) \;\!\mathrm {d}z . \end{aligned}$$

According to Sect. 6.1 the functional $\widetilde{\mathsf {E}}^V_{\mathsf {Y}}= \widetilde{\varvec{E}}_V \circ \Phi _V$ on ${\mathsf {Y}}$ is then given by

$$\begin{aligned} \widetilde{\mathsf {E}}^V_{\mathsf {Y}}( \varrho _s,\widehat{\varvec{c}}_{\mathrm {m}})&= \int _{{\varvec{C}}_{\mathrm {s}}} \Big (\frac{1}{V} \rho _s({\varvec{c}}_s)\log \rho _s({\varvec{c}}_{\mathrm {s}}) + \rho _s({\varvec{c}}_{\mathrm {s}}) E_{\mathrm {s}}({\varvec{c}}_{\mathrm {s}}) \Big )\;\!\mathrm {d}{\varvec{c}}_{\mathrm {s}}+ \frac{\widetilde{Z}(V)}{V} +\sum _{j=J+1}^I \widehat{e}_V(\widehat{c}_j,c^*_j) \\&\quad \text {where } \widehat{e}_V(\widehat{a},a^*) := A(V\widehat{a})\widehat{a} \log \big (\frac{\widehat{a}}{a^*}\big ) -\widehat{a} + a^*- \frac{\log \big (\widehat{a} Z(V\widehat{a})\big )}{V} \end{aligned}$$

with $$E_{\mathrm {s}}({\varvec{c}}_{\mathrm {s}}) = \sum _{i=1}^J c_i^* \lambda _{\mathrm {B}}(c_i/c_i^*)$$ and $$A(v)= \int _0^\infty z \exp \big (-v \lambda _{\mathrm {B}}(z)\big ) \;\!\mathrm {d}z/Z(v)$$.

It can be shown that $A(v)\ge 1$ and $e_V(\widehat{a}, a^*)\ge a^*\lambda _{\mathrm {B}}(\widehat{a}/a^*)$ for all *V*, and for $V\rightarrow \infty$ we obtain $e_V(\widehat{a}, a^*)\rightarrow a^*\lambda _{\mathrm {B}}(\widehat{a}/a^*)$. To simplify the model we are therefore allowed to replace the last term in $\widetilde{\mathsf {E}}^V_{\mathsf {Y}}$ by the relative entropy $E_{\mathrm {m}}(\widehat{\varvec{c}}_{\mathrm {m}}) =\sum _{j=J+1}^I c_j^* \lambda _{\mathrm {B}}(\widehat{c}_j/c_j^*)$ for the RRE. Neglecting the irrelevant constant term ${\widetilde{Z}(V)}/{V}$, we obtain the hybrid energy again as a relative entropy, namely

$$\begin{aligned} {\mathfrak {E}}^\text {FP-RR}_V(\varrho _s,\widehat{\varvec{c}}_{\mathrm {m}}) = \int _{{\varvec{C}}_{\mathrm {s}}}\Big ( \frac{1}{V} \rho _s({\varvec{c}}_{\mathrm {s}})\log \rho _s({\varvec{c}}_{\mathrm {s}}) + \rho _s({\varvec{c}}_{\mathrm {s}}) E_{\mathrm {s}}({\varvec{c}}_{\mathrm {s}})\Big ) \;\!\mathrm {d}{\varvec{c}}_{\mathrm {s}}+ E_{\mathrm {m}}(\widehat{\varvec{c}}_{\mathrm {m}}) . \end{aligned}$$

For the Onsager operator we also use a cruder reduction than the minimization advocated in Sect. 6.1. We simply postulate the Onsager operator ${\mathsf {K}}_V$ via the dual dissipation potential

$$\begin{aligned} \Psi ^*_{V,\text {FP-RR}}(\varrho _s,\widehat{\varvec{c}}_{\mathrm {m}}; \xi ,\zeta ) = \frac{1}{2}\int _{{\varvec{C}}_{\mathrm {s}}} \rho _s({\varvec{c}}_{\mathrm {s}}) \left( {\begin{array}{c}\nabla _s \xi ({\varvec{c}}_{\mathrm {s}})\\ \zeta \end{array}}\right) \cdot {\mathbb {K}}({\varvec{c}}_{\mathrm {s}},\widehat{\varvec{c}}_{\mathrm {m}}) \left( {\begin{array}{c}\nabla _s \xi ({\varvec{c}}_{\mathrm {s}})\\ \zeta \end{array}}\right) \;\!\mathrm {d}{\varvec{c}}_{\mathrm {s}}, \end{aligned}$$

where $\xi \in {\mathrm {C}}^1({\varvec{C}}_{\mathrm {s}})$ and $\zeta \in {\mathbb {R}}^{I-J}$. Indeed, in the sense of the general reduction method explained in Sect. 6.1 we see that ${\mathfrak {K}}^\text {FP-RR}_V$ is obtained from ${\varvec{K}}$ by inserting $\varrho = \varrho _s({\mathrm {d}}{\varvec{c}}_{\mathrm {s}}){\otimes }\delta _{\widehat{\varvec{c}}_{\mathrm {m}}}$ and $\Xi ={\mathsf {M}}(\xi ,\zeta ):({\varvec{c}}_{\mathrm {s}},{\varvec{c}}_{\mathrm {m}})\mapsto \xi ({\varvec{c}}_{\mathrm {s}})+ \zeta {\cdot }{\varvec{c}}_{\mathrm {m}}$.

Thus, the hybrid model induced by the gradient system $({\mathscr {P}}({\varvec{C}}_{\mathrm {s}}){\times }{\varvec{C}}_{\mathrm {m}}, {\mathfrak {E}}^\text {FP-RR}_V,{\mathfrak {K}}^\text {FP-RR}_V)$ is given by the coupled system for $\rho \in {\mathscr {P}}({\varvec{C}}_{\mathrm {s}})$ and $\widehat{\varvec{c}}_{\mathrm {m}}\in {\varvec{C}}_{\mathrm {m}}$:

$$\begin{aligned} {\dot{\rho }}({\varvec{c}}_{\mathrm {s}})&= {\mathrm {div}}_{\mathrm {s}}\Big ( {\mathbb {K}}_{\mathrm{s{\mathrm {s}}}}({\varvec{c}}_{\mathrm {s}},\widehat{\varvec{c}}_{\mathrm {m}})\big (\tfrac{1}{V}\nabla _{\mathrm {s}}\rho ({\varvec{c}}_{\mathrm {s}}) {+} \rho ({\varvec{c}}_{\mathrm {s}})\nabla _{\mathrm {s}}E_{\mathrm {s}}({\varvec{c}}_{\mathrm {s}})\big ) + \rho ({\varvec{c}}_{\mathrm {s}}) {\mathbb {K}}_{\mathrm{s{\mathrm {m}}}}({\varvec{c}}_{\mathrm {s}},\widehat{\varvec{c}}_{\mathrm {m}})\nabla _{\mathrm {m}}E_{\mathrm {m}}(\widehat{\varvec{c}}_{\mathrm {m}}) \Big ) ,\\ \dot{\widehat{\varvec{c}}}_{\mathrm {m}}&= - \int _{{\varvec{C}}_{\mathrm {s}}}\Big ( {\mathbb {K}}_{\mathrm{s{\mathrm {m}}}}^\mathsf {T}({\varvec{c}}_{\mathrm {s}},\widehat{\varvec{c}}_{\mathrm {m}}) \big (\tfrac{1}{V} \nabla _{\mathrm {s}}\rho ({\varvec{c}}_{\mathrm {s}}){+} \rho ({\varvec{c}}_{\mathrm {s}})\nabla _{\mathrm {s}}E_{\mathrm {s}}({\varvec{c}}_{\mathrm {s}})\big ) \\&\quad + \rho ({\varvec{c}}_{\mathrm {s}}){\mathbb {K}}_{\mathrm{m {\mathrm {m}}}}({\varvec{c}}_{\mathrm {s}},\widehat{\varvec{c}}_{\mathrm {m}}) \nabla _{\mathrm{m}} E_{\mathrm {m}}(\widehat{\varvec{c}}_{\mathrm {m}}) \Big ) \;\!\mathrm {d}{\varvec{c}}_{\mathrm {s}}. \end{aligned}$$

It is interesting to see that the last terms can be rewritten in terms of the RRE ${\dot{{\varvec{c}}}} = - {\mathbb {K}}({\varvec{c}}) {\mathrm {D}}E({\varvec{c}})= -{\varvec{R}}({\varvec{c}})= -({\varvec{R}}_{\mathrm {s}}({\varvec{c}}_{\mathrm {s}},\widehat{\varvec{c}}_{\mathrm {m}}),{\varvec{R}}_{\mathrm {m}}({\varvec{c}}_{\mathrm {s}},\widehat{\varvec{c}}_{\mathrm {m}}))$, viz.

$$\begin{aligned} {\dot{\rho }}({\varvec{c}}_{\mathrm {s}})&= {\mathrm {div}}_{\mathrm {s}}\Big (\tfrac{1}{V} {\mathbb {K}}_{\mathrm{s{\mathrm {s}}}}({\varvec{c}}_{\mathrm {s}},\widehat{\varvec{c}}_{\mathrm {m}})\nabla _{\mathrm {s}}\rho ({\varvec{c}}_{\mathrm {s}}) + \rho ({\varvec{c}}_{\mathrm {s}}) {\varvec{R}}_{\mathrm {s}}({\varvec{c}}_{\mathrm {s}},\widehat{\varvec{c}}_{\mathrm {m}}) \Big ) ,\\ \dot{\widehat{\varvec{c}}}_{\mathrm {m}}&= - \int _{{\varvec{C}}_{\mathrm {s}}} \Big (\tfrac{1}{V} {\mathbb {K}}_{\mathrm{s{\mathrm {m}}}}^\mathsf {T}({\varvec{c}}_{\mathrm {s}},\widehat{\varvec{c}}_{\mathrm {m}}) \nabla _{\mathrm {s}}\rho ({\varvec{c}}_{\mathrm {s}}) + \rho ({\varvec{c}}_{\mathrm {s}}) {\varvec{R}}_{\mathrm {m}}( {\varvec{c}}_{\mathrm {s}}, \widehat{\varvec{c}}_{\mathrm {m}}) \Big ) \;\!\mathrm {d}{\varvec{c}}_{\mathrm {s}}. \end{aligned}$$

This reveals that the system is a classical mean-field model, which is linear in the density $\rho$ for the component ${\varvec{c}}_{\mathrm {s}}$ while it is nonlinearly coupled to the ODE for the component $\widehat{\varvec{c}}_{\mathrm {m}}$.

### Coupling a RRE to a CME

In analogy to the coupling of an RRE for some macroscopic ${\varvec{c}}_{\mathrm {m}}$ to a Fokker–Planck equation we can directly couple the CME to an RRE, which leads to hybrid system defined on ${\mathscr {P}}({\mathbb {N}}_0^J){\times }{[0,\infty [}^{I-J}$. Instead of given the general derivation as in Sect. 6.3, we just give an explicit example.

For $\beta \in {\mathbb {N}}_0$ we consider the simple reaction  with stoichiometric vectors ${\varvec{\alpha }}=(1,0)$, ${\varvec{\beta }}=(0,\beta )$, and ${\varvec{\gamma }}=(1,-\beta )$. The associated system of RREs is given by

6.5 $$\begin{aligned} {\dot{c}}_1 = c_2^\beta - c_1,\quad {\dot{c}}_2 = \beta \,(c_1{-}c_2^\beta ). \end{aligned}$$

We have the conservation relation ${\mathbb {Q}}{\varvec{c}}= \beta c_1 + c_2 = q$ and the detailed-balance steady state ${\varvec{c}}_*=(1,1)^\top$. The associated CME on ${\mathcal {N}}={\mathbb {N}}_0^2$ takes the form

6.6 $$\begin{aligned} {\dot{u}}_{\varvec{n}}= (n_1{+}1) u_{{\varvec{n}}+(1,-\beta )} - \Bigg ( n_1 + \frac{n_2!}{V^{\beta -1} (n_2{-}\beta )!}c\Bigg )u_{\varvec{n}}+ \frac{(n_2{+}\beta )!}{V^{\beta -1}n_2!} \,u_{{\varvec{n}}+(-1,\beta )} \text { for } {\varvec{n}}\in {\mathcal {N}}. \end{aligned}$$

The detailed-balance steady state by ${\varvec{w}}^V_{\varvec{n}}= (w_{n_1}^V w_{n_2}^V)_{{\varvec{n}}\in {\mathcal {N}}}$ with $w^V_n = {\mathrm {e}}^{-V}V^n/n!$. As in (4.5b) the full Onsager operator ${\mathcal {K}}_V$ is defined via

$$\begin{aligned} \langle {{\varvec{\mu }},{\mathcal {K}}_V({\varvec{u}}){\varvec{\mu }}}\rangle = \sum _{{\varvec{n}}\in {\mathcal {N}}} \Lambda \bigg (\tfrac{n_1{+}1}{V}u_{{\varvec{n}}{+}(1,0)}, \tfrac{(n_2{+}\beta )!}{V^\beta n_2!} u_{{\varvec{n}}+(0,\beta )} \bigg ) \big (V(\mu _{{\varvec{n}}+(1,0)} {-} \mu _{{\varvec{n}}+(0,\beta )})\big )^2. \end{aligned}$$

We partition ${\varvec{c}}=(c_1,c_2)=(c_{\mathrm {s}},c_{\mathrm {m}})$, i.e., we keep $c_1\in {[0,\infty [}$ in stochastic description via the distribution ${\varvec{v}}=(v_m)_{m \in {\mathbb {N}}_0}\in {\mathscr {P}}({\mathbb {N}}_0)$, while $c_2\in {[0,\infty [}$ will be treated macroscopically. Thus, we define the gradient system $({\mathscr {P}}({\mathbb {N}}_0){\times }{[0,\infty [}),{\mathfrak {E}}_V^\text {CM-RR},{\mathfrak {K}}_V^\text {CM-RR})$ with relative entropy and Onsager operator defined via

$$\begin{aligned} {\mathfrak {E}}^\text {CM-RR}_V({\varvec{v}},c_2)&= {\mathsf {E}}(c_2)+ \frac{1}{V} \sum _{m\in {\mathbb {N}}_0} v_m\log (v_m/w^V_m) , \quad \text {where } {\mathsf {E}}(z)=\lambda _{\mathrm {B}}(z),\\ \langle {\genfrac(){0.0pt}1{{\varvec{\xi }}}{\zeta }, {\mathfrak {K}}^\text {CM-RR}_V({\varvec{v}},c_2)\genfrac(){0.0pt}1{{\varvec{\xi }}}{\zeta }}\rangle&= V\sum _{m\in {\mathbb {N}}_0} \Lambda \big ( \tfrac{m{+}1}{V}v_{m+1} , v_m c_2^\beta \big ) \big ( \xi _m{+}\tfrac{\beta }{V} \zeta - \xi _{m+1}\big )^2, \end{aligned}$$

for ${\varvec{\xi }}: {\mathbb {N}}_0 \rightarrow {\mathbb {R}}$ and $\zeta \in {\mathbb {R}}$. Again, ${\mathfrak {K}}^\text {CM-RR}_V$ is obtained from ${\mathcal {K}}_V$ by inserting ${\varvec{u}}_{m,n_2}= v_m \delta _{\lfloor Vc_2 \rfloor }(n_2)$ and $\Xi = {\mathsf {M}}({\varvec{\xi }},\zeta ): (m,n_2) \mapsto \xi _m +\frac{1}{V} n_2\zeta$ and performing an approximation for large *V*. The associated evolution equation is the hybrid system

$$\begin{aligned}&{\dot{v}}_m= Vc_2^\beta v_{m-1} -\big (m+ Vc_2^\beta \big )v_m + (m{+}1)v_{m+1} \quad \text {for }m\in {\mathbb {N}}_0 \ \text { (with }v_{-1}=0\text {)},\\&{\dot{c}}_2= \beta \Bigg ( \frac{1}{V} \sum _{m\in {\mathbb {N}}} m v_m - c_2^\beta \Bigg ). \end{aligned}$$

Clearly, this system is consistent with the conservation law ${\mathbb {Q}}{\varvec{c}}= \beta c_1+c_2=\text {const.}$, in the sense that $c_1 := \frac{1}{V}\sum _{m\in {\mathbb {N}}} m v_m$ satisfies ${\dot{c}}_1 = c_2^\beta - c_1 = - {\dot{c}}_2 / \beta$.

### Combining CME and Fokker–Planck Descriptions

We consider the simplest nontrivial model, namely the scalar RRE ${\dot{c}} = a - b c$ with $a, b > 0$, which is induced by the reaction . This corresponds to $\alpha = 0$, $\beta = 1$, $k_{\mathrm {fw}}= a$, and $k_{\mathrm {bw}}= b$. We have the following three derived gradient systems:

1. The RRE ${\dot{c}} = a-bc$ is generated by the gradient system $({\mathbb {R}}_+, {\mathbb {K}},E)$ with steady state $c_*=a/b$, ${\mathbb {K}}(c) = \Lambda (a, bc)$, and $E(c) = \frac{a}{b} \lambda _{\mathrm {B}}(bc/a)$.
2. The associated chemical master equation ${\dot{{\varvec{u}}}} ={\mathcal {B}}_V {\varvec{u}}$ is generated by the gradient system $({\mathscr {P}}({\mathbb {N}}_0),{\mathcal {E}}_V,{\mathcal {K}}_V)$ and reads
  6.7 $$\begin{aligned} {\dot{u}}_n = Va u_{n-1} - \big ( Va {+}bn\big ) u_n + b(n{+}1)u_{n+1} \quad \text {for }n\in {\mathbb {N}}_0 \quad (\text {with }u_{-1}=0) \end{aligned}$$
  and has the steady state ${\varvec{w}}_V=({\mathrm {e}}^{-Va/b} (Va/b)^n/n!)_{n\in {\mathbb {N}}_0}$. The entropy and Onsager operator are
  $$\begin{aligned}&{\mathcal {E}}_V({\varvec{u}})= \frac{1}{V}\sum _{n\in {\mathbb {N}}_0}u_n \log \big (u_n/w^V_n\big ) \quad \text {and} \\&{\mathcal {K}}_V({\varvec{u}})= V^2a\sum _{n\in {\mathbb {N}}_0} w^V_n \Lambda \bigg (\tfrac{u_n}{w^V_n},\tfrac{u_{n+1}}{w^V_{n+1} }\bigg ) ({\varvec{e}}_{n}{-}{\varvec{e}}_{n+1})\otimes ({\varvec{e}}_n{-}{\varvec{e}}_{n+1}). \end{aligned}$$
3. The associated Fokker–Planck equation (5.4) takes the form
  6.8 $$\begin{aligned} {\dot{\rho }} = \partial _c\Bigg (\frac{\Lambda (a,bc)}{V} \partial _c\rho + \bigg ( bc{-}a +\frac{\Lambda (a,bc)}{2Vc{+}1/3} \bigg )\rho \Bigg ) \text { for }t,c>0\text { and } \rho (t,0)=0. \end{aligned}$$
  This equation has the equilibrium solution $W_V:c \mapsto {\mathsf {W}}(c;a/b,V)$ (cf. (5.2)) and is generated by the gradient system $({\mathscr {P}}({]0,\infty [}),{\varvec{E}}_V,{\varvec{K}})$ with
  $$\begin{aligned}&{\varvec{E}}_V(\rho ) = \frac{1}{V}\int _0^\infty \rho (c) \log \Big (\frac{\rho (c)}{W_V(c)}\Big )\;\!\mathrm {d}c\quad \text {and} \quad {\varvec{K}}(\rho )\xi = - \big (\rho \Lambda (a,bc) \xi '\big )'. \end{aligned}$$

To combine the description via the CME and the Fokker–Planck equation we consider the mixed state space ${\mathfrak {N}}:=\{0,1,\ldots ,N{-}1\}\cup {[N/V,\infty [}$. Hence, $n\in \{0,\ldots ,N{-}1\}$ counts the number of atoms, while for $n\ge N$ we use the concentration $c=n/V \ge N/V$ as a continuous variable to describe the state. A typical choice could be $1 \ll V \ll N$ to be sure to capture all small discrete effects.

The hybrid gradient system $({\mathscr {P}}({\mathfrak {N}}),{\mathfrak {E}}_{V,N},{\mathfrak {K}}_{V,N})$ is described by measures

$$\begin{aligned} {\mathfrak {u}}= \sum _{n=0}^{N-1} u_n\delta _{n} + U(c) \;\!\mathrm {d}c|_{[N/V,\infty [} \ \in {\mathscr {P}}({\mathfrak {N}}). \end{aligned}$$

The idea is now to choose ${\mathfrak {E}}_{V,N}$ and ${\mathfrak {K}}_{V,N}$ rather than to model the evolution equation.

We first choose the equilibrium state in the form

$$\begin{aligned} {\mathfrak {w}}^{V,N} = \sum _{n=0}^{N-1} w^V_n\delta _n + W^V(c) \;\!\mathrm {d}c:= \sum _{n=0}^{N-1} {\mathrm {e}}^{-Va/b}\frac{(Va/b)^n}{n!} \delta _{n} + \frac{1}{Z_{V,N}} W_V(c)\,\;\!\mathrm {d}c , \end{aligned}$$

where $$Z_{V,N} $$ is uniquely determined by asking $$\int _{\mathfrak {N}}\;\!\mathrm {d}{\mathfrak {w}}^{V,N} =1$$. The entropy functional is defined via the obvious relative entropy per volume, namely

$$\begin{aligned} {\mathfrak {E}}_{V,N}({\mathfrak {u}})&= \frac{1}{V} \int _{\mathfrak {N}}\log \Big (\frac{{\mathrm {d}}{\mathfrak {u}}}{{\mathrm {d}}{\mathfrak {w}}^{V,N}}\Big ) \;\!\mathrm {d}{\mathfrak {u}}\\&= \frac{1}{V} \sum _{n=0}^{N-1} \lambda _{\mathrm {B}}\bigg (\frac{u_n}{w_n^V} \bigg )w_n^V + \frac{1}{V}\int _{N/V}^\infty \lambda _{\mathrm {B}}\bigg ( \frac{U(c)}{W_V(c)}\bigg ) W_V(c)\;\!\mathrm {d}c , \end{aligned}$$

where $\frac{{\mathrm {d}}{\mathfrak {u}}}{{\mathrm {d}}{\mathfrak {w}}}$ denotes the Radon–Nikodym derivative.

The difficult part is the modeling of the Onsager operator ${\mathfrak {K}}_{V,N}({\varvec{u}})$ as it includes the crucial transfer between the discrete and the continuous parts of the hybrid model. We define ${\mathfrak {K}}$ in terms of its associated quadratic form acting on smooth functions $\xi :{\mathfrak {N}}\rightarrow {\mathbb {R}}$, where we write $\xi _n$ for $\xi (n)$ and *W*(*c*) for $W_V(c)$:

$$\begin{aligned} \langle \xi , {\mathfrak {K}}_{V,N}({\varvec{u}})\xi \rangle&= V^2 a \sum _{n=1}^{N-1} w_{n-1}\Lambda \big (\tfrac{u_{n-1}}{w_{n-1}} , \tfrac{u_n}{w_n}\big ) (\xi _{n-1}{-}\xi _n)^2 \\&\quad + V^2 \widehat{a} w_{N-1}\,\Lambda \big (\tfrac{u_{N-1}}{w_{N-1}}, \tfrac{U(N/V)}{W(N/V)} \big ) \big ( \xi _{N-1}{-}\xi (N/V)\big )^2 \\&\quad + \int _{N/V}^\infty \Lambda (a,bc) \xi '(c)^2 U(c) \;\!\mathrm {d}c . \end{aligned}$$

While the first and the third terms on the right-hand side give the purely discrete and the continuous parts of the state space, respectively, we see that the second term is the new term that couples the discrete and the continuous parts. The parameter $\widehat{a}$ is still to be chosen, the natural parameter being *a*.

The evolution equation for ${\mathfrak {u}}$ is again a linear equation of the form ${\dot{{\mathfrak {u}}}} = {\mathfrak {B}}_{V,N} {\mathfrak {u}}$, i.e., it corresponds to a continuous-time Markov process. It consists of a discrete part, as in (6.7) but only for $n=0,\ldots ,N-2$, and a continuous part, as in (6.8) but only for $c>N/V$. The new structure is the coupling between the two subsystems which gives rise to the following conditions:

$$\begin{aligned} {\dot{u}}_{N-1}&= V a\, u_{N-2} - \big (V\widehat{a} +b\, (N{-}1) \big ) u_{N-1} + V\widehat{a} \,\frac{w_{N-1}}{W(N/V)}\,U(N/V), \\ 0&= V\widehat{a}\, \Big (\frac{w_{N-1}}{W(N/V)}\,U(N/V) - u_{N-1} \Big ) + \frac{1}{V} W(N/V) \Lambda (a, b N/V)\Big ( \frac{U}{W}\Big )'(N/V). \end{aligned}$$

By our definition of ${\mathfrak {w}}^{V,N}$ we have $\frac{w_{N-1}}{W(N/V)} \approx Nb/(aV^2)$ and see that for $\widehat{a} =a$ these conditions take the approximate form

$$\begin{aligned} {\dot{u}}_{N-1}&\approx V a\, u_{N-2} - \big (V a +b\, (N{-}1) \big ) u_{N-1} + b \tfrac{N}{V}\,U( \tfrac{N}{V}), \\ 0&\approx \tfrac{1}{V} \Lambda \big (a, b \tfrac{N}{V}\big ) U' \big (\tfrac{N}{V} \big ) + b\tfrac{N}{V} U\big (\tfrac{N}{V}\big ) - aV\,u_{N-1}, \end{aligned}$$

where the second relation clearly shows the corresponding Robin boundary condition connecting the parabolic Fokker–Planck equation to the discrete system on $\{0,\ldots ,N{-}1\}$. Note that $u_n$ and *U* are scaled such that $V u_{N-1}$ is comparable to *U*(*N*/*V*).
